# FDA-approved kinase inhibitors in PROTAC design, development and synthesis

**DOI:** 10.1080/14756366.2025.2542357

**Published:** 2025-08-12

**Authors:** Kacper Kossakowski, Alina Cherniienko, Lucjusz Zaprutko, Anna Pawełczyk

**Affiliations:** ^a^ Department and Chair of Organic Chemistry, Poznan University of Medical Sciences, Poznan, Poland; ^b^ Doctoral School of Poznan University of Medical Sciences, Poznan University of Medical Sciences, Poznan, Poland

**Keywords:** Proteolysis-targeting chimaera (PROTAC), targeted protein degradation (TPD), kinase inhibitor, FDA-approved drug, cancer

## Abstract

FDA-approved kinase inhibitors represent a rapidly growing class of targeted therapies with proven clinical success in oncology. However, their occupancy-driven mode of action is often associated with resistance, off-target effects, and incomplete inhibition. Proteolysis-Targeting Chimaeras (PROTACs) offer a compelling alternative by promoting complete degradation of oncogenic kinases, thereby enhancing selectivity and resistance reduction. In this review, we provide a comprehensive overview of the rational design, development, and synthetic approaches for PROTACs incorporating FDA-approved kinase inhibitors. We discuss key aspects influencing degrader efficiency, including kinase selectivity, linker design, E3 ligase recruitment, and synthetic strategies. Additionally, we highlight recent advances, emerging trends, and future directions, such as expanding the repertoire of degradable kinases, optimising linker chemistry, and broadening diversity of E3 ligases. A better understanding of these factors will facilitate the continued evolution of PROTAC technology into effective next-generation therapies for kinase-driven diseases.

## Graphical Abstract

**Figure:**
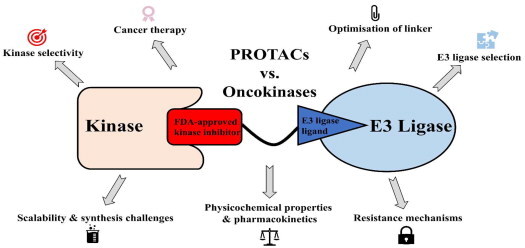

## Introduction

Kinase inhibitors have revolutionised targeted cancer therapy, delivering significant progress in treating diseases driven by dysregulated kinase activity^1^. However, their occupancy-based mechanism of action (MOA) still faces challenges, particularly regarding the long-term effectiveness and wider therapeutic application^2^. Occupancy-based therapeutics exert activity on the target exclusively in the bound state. This translates into a requirement for dosing which is sufficiently high and frequent to continually saturate the majority of target copies. Over time, adaptive resistance mechanisms such as target overexpression, gene amplification, drug efflux, or point mutations at the drug’s binding site reduce their effectiveness and increase the risk of off-target toxicity^3^^,^^4^.

**Figure 1.**
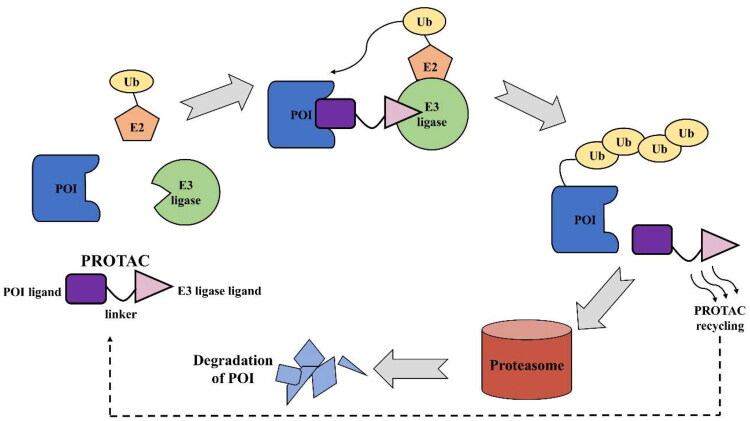
The mechanism of PROTAC-mediated targeted protein degradation (the figure was drawn by the authors using PowerPoint).

Proteolysis-targeting chimaeras (PROTACs) offer an innovative alternative by utilising targeted protein degradation (TPD) to eliminate disease-relevant kinases^5–7^. PROTACs are heterobifunctional compounds consisting of a ligand for a target protein of interest (POI) and a ligand for an E3 ubiquitin ligase linked by a linker^8–10^. These molecules engage the ubiquitin-proteasome system (UPS) to degrade target proteins by bringing them into proximity with an E3 ligase, thereby facilitating the tagging of the POI with a polyubiquitin chain. Ubiquitin-tagged POIs are then recognised by the proteasome, where they undergo degradation through the proteolytic process (Figure 1). Unlike occupancy-based inhibitors, PROTACs operate through an event-driven mechanism, wherein the formation of a transient ternary complex initiates degradation, allowing a single PROTAC molecule to degrade multiple target protein equivalents. This approach reduces the need for continuous target saturation and minimises off-target effects, enabling the degradation of previously undruggable targets and broadening the druggable kinome^11^.

A key factor in PROTAC design is the selection of an appropriate E3 ubiquitin ligase. Although the human genome encodes over 600 E3 ligases, few have been successfully exploited for PROTAC-mediated degradation^7^^,^^12^^,^^13^. The vast majority of PROTACs developed to date utilise either cereblon (CRBN) or von Hippel-Lindau (VHL) as E3 ligase recruiters, owing to their well-characterised binding ligands and robust ubiquitination efficiency. Other ligases, such as MDM2, cIAP1, and RNF114, have also been explored, but their use remains less common. Expanding the repertoire of E3 ligases for PROTAC development is an area of ongoing research, with efforts focused on identifying novel ligands and broadening the applicability of targeted protein degradation strategies^12^.

The application of PROTAC technology into drug development is consistent with the broader trend of advanced therapeutics, including other TPD approaches such as molecular glue, LYTAC, abTAC, GlueTAC, and analogous strategies^14–16^. The integration of FDA-approved kinase inhibitors as structural components of PROTAC molecules offers a promising way to combine the strengths of established drugs with the potential for targeted protein degradation. Kinase inhibitors utilise their well-characterised pharmacological profiles, including known binding affinities, safety data, and off-target effects, to accelerate development and predict therapeutic outcomes. This strategy may be aligned with the concept of drug repurposing, wherein existing drugs are adapted for new therapeutic applications^17^^,^^18^. By combining the specificity of kinase inhibitors with the advantages of PROTAC technology, this approach expands treatment possibilities and addresses limitations associated with traditional inhibitors^2^.

Despite the growing interest in PROTAC technology, there remains a significant gap in the literature concerning the integration of FDA-approved kinase inhibitors as structural components into PROTAC molecules. Given the pivotal role of kinase inhibitors in targeted cancer therapy and their proven efficacy in clinical practice, their rational incorporation into PROTACs offers great potential for overcoming resistance mechanisms and expanding the therapeutic scope. In this review, we aim to bridge this gap by providing a comprehensive overview of their rational design, development, and synthesis. We highlight all the FDA-approved kinase inhibitors successfully incorporated into PROTACs. We summarise current progress and outline key experimental aspects, providing the foundation for future research efforts towards the development of next-generation PROTACs and the strategic repurposing of kinase inhibitors for targeted protein degradation in kinase-driven diseases.

## Methodology

**Figure 2.**
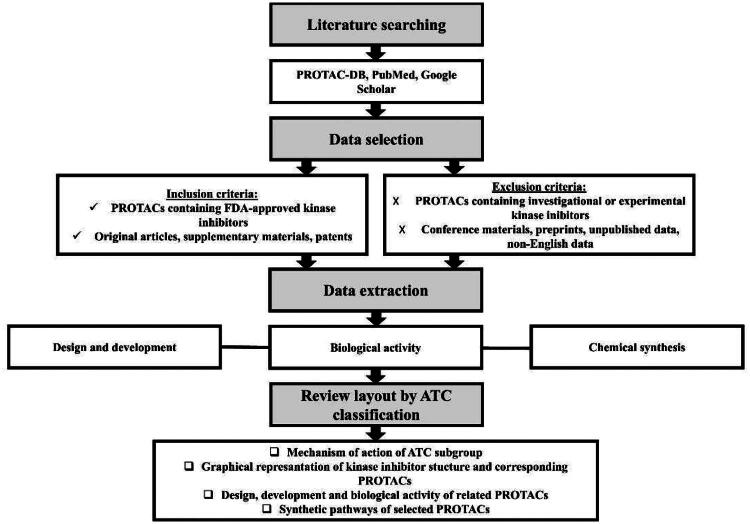
Flowchart of methodology (the figure was drawn by the authors using PowerPoint).

The search for studies related to PROTACs containing FDA-approved kinase inhibitors was conducted using the PROTAC-DB 3.0 database^19^, updated in September 2024, where the methodology for data collection was based on a manual search of the PubMed database, utilising the keywords “degrader”, “PROTAC” OR “proteolysis targeting chimera”. To identify the relevant PROTACs, we manually entered all FDA-approved kinase inhibitors in the “warheads” section of PROTAC-DB, based on their classification according to the WHO Anatomical Therapeutic Chemical (ATC) system^20^, specifically group L01E and subgroups L04AF and L04AH (Figure 2). In addition, to ensure the most up-to-date dataset and include all available publications beyond PROTAC-DB, we performed an independent search using PubMed and Google Scholar. This search was conducted in December 2024 with the keywords “[name of kinase inhibitor]” AND “PROTAC” OR “proteolysis targeting chimera”. Kinase inhibitors classified as “investigational” or “experimental” were excluded from this review (Figure 2).

For each kinase inhibitor, we considered all related available original articles, their supplementary materials, and patents, focusing on identifying the most potent PROTACs incorporating the kinase inhibitor as the POI ligand. We interpreted a chemical structure as a kinase inhibitor if the structure was unchanged, or the only structural modification was the functionalisation of a single functional group or moiety to attach the linker, or if a subtle structural change occurred in a part of the molecule not directly connected to the linker.

The selected studies were analysed based on the design and development methods of the PROTACs, their chemical synthesis pathways, and biological activity. The synthesis pathways presented are concise, focusing only on the synthesis of the POI ligand, if available, its functionalisation, and conjugation with the E3 ligase ligand via a linker. The synthesis methods of the E3 ligase ligands and their functionalisation were not included (unless its were not described in the mentioned papers below), as comprehensive works on this topic have already been published^13^^,^^21–23^. Whenever possible, quantitative data describing PROTAC and kinase inhibitors activity - such as IC_50_ (half maximal inhibitory concentration), DC_50_ (half maximal degradation concentration), D_max_ (maximum degradation achieved) values - are provided to allow for more detailed comparisons of biological efficacy. However, due to the lack of consistency among reviewed publications, such values were not always available. In several cases, biological activity was presented only in the form of qualitative data (e.g. Western blot images or degradation curves) without clearly reported numerical values. This heterogeneity of reporting complicates the objective and standardised assessment of PROTAC performance across studies.

The following sections of the review are organised by kinase inhibitor groups in accordance with the ATC classification. If none of the kinase inhibitors of a given ATC group were utilised in the PROTAC technology, the group would not be considered for further part of the review. Each subsection includes a graphical representation of the kinase inhibitor structure, along with its trade name and corresponding PROTACs based on that structure. In these graphics, the POI ligand (in red), linker (in black), and E3 ligase ligand (in blue) are clearly marked. For each drug subgroup, a brief overview of its mechanism of action on the specific kinase is provided, followed by a detailed description of the design, development, and biological activity of the related PROTACs. Additionally, a summary of the synthetic pathways for all PROTACs associated with the selected kinase inhibitor is included in each section (with some exceptions, which are explained in the subsections on the synthesis of selected PROTACs).

## Kinase inhibitors

Kinase inhibitors are a class of targeted therapy drugs used to modulate the activity of specific protein kinases, enzymes that regulate numerous cellular processes, including cell growth, differentiation, and survival^1^^,^^24^^,^^25^. By targeting these kinases, inhibitors interrupt aberrant signalling pathways that drive diseases such as cancer and autoimmune disorders. Kinases themselves are often involved in phosphorylating other proteins, a process crucial for activating or deactivating key regulatory proteins. Dysregulation of kinase activity, whether through mutation or overexpression, leads to many pathologies, particularly malignancies.

Kinase inhibitors are classified in the ATC system into various groups, including L01E (kinase inhibitors used as antineoplastic agents) and L04AF/L04AH (kinase inhibitors used as immunosuppressants). The differences between these groups primarily lie in their therapeutic applications. Inhibitors from the L01E group are designed to target kinases involved in the pathological proliferation of cancer cells, while inhibitors from the L04AF and L04AH groups aim to modulate immune responses and inhibit signalling pathways responsible for inflammation.

Some of these kinase inhibitors have been incorporated into PROTAC technology, as illustrated in Table 1. This innovative approach offers the potential to overcome limitations such as resistance or incomplete inhibition commonly associated with traditional kinase inhibitors. However, challenges remain in adapting certain kinase inhibitors into PROTAC molecules, particularly due to structural or mechanistic constraints that may limit the development of effective PROTACs.

**Table 1.** FDA-approved kinase inhibitors incorporated or not incorporated into PROTAC technology in accordance with the established methodology.

ATC group	Incorporated into PROTAC technology	Not incorporated into PROTAC technology
L01EA (BCR-ABL tyrosine kinase inhibitors)	Dasatinib^26–36^	Nilotinib
	Asciminib^33^^,^^37^	
	Ponatinib^33^	
	Bosutinib^26^	
	Imatinib*^26^^,^^27^^,^^33^	
L01EB (EGFR tyrosine kinase inhibitors)	Dacomitinib^38^	Erlotinib Lazertinib Mobocertinib
	Rociletinib^39^	
	Osimertinib^40^	
	Afatinib^41^	
	Gefitinib^41–43^	
L01EC (BRAF serine-threonine kinase inhibitors)	Dabrafenib^44^^,^^45^	—
	Vemurafenib^46–48^	
	Encorafenib^49^	
L01ED (ALK inhibitors)	Brigatinib^50–52^	Lorlatinib
	Alectinib^53^^,^^54^	
	Crizotinib^55^	
	Ceritinib^56–62^	
L01EE (MEK inhibitors)	—	Trametinib
		Cobimetinib
		Binimetinib
		Selumetinib
L01EF (CDK inhibitors)	Palbociclib^63–71^	—
	Ribociclib^63^^,^^64^^,^^66^	
	Abemaciclib^64^^,^^66^	
L01EG/L04AH (mTOR kinase inhibitors)	—	Temsirolimus
		Everolimus
		Sirolimus
L01EH (HER2 tyrosine kinase inhibitors)	—	Lapatinib
		Neratinib
		Tucatinib
L01EJ/L04AF (JAK inhibitors)	Ruxolitinib^72^ Baricitinib^72^^,^^73^	Fedratinib
		Pacritinib
		Momelotinib
L01EK (VEGFR tyrosine kinase inhibitors)	—	Axitinib
		Tivozanib
		Fruquintinib
L01EL (BTK tyrosine kinase inhibitors)	Ibrutinib^74–78^	Acalabrutinib
	Zanubrutinib^79^	Pirtobrutinib
L01EM (Pi3K tyrosine kinase inhibitors)	—	Idelalisib
		Copanlisib
		Alpelisib
		Duvelisib
L01EN (EGFR tyrosine kinase inhibitors)	—	Erdafitinib
		Pemigatinib
		Infigratinib
		Futibatinib
L01EX (other protein kinase inhibitors)	Sorafenib^80^^,^^81^Tepotinib^82^Quizartinib^83^^,^^84^Entrectinib^85^Gilteritinib^86^^,^^87^	Sunitinib
		Pazopanib
		Vandetanib
		Regorafenibu
		Cabozantinib
		Lenvatinib
		Nintedanib
		Midostaurin
		Larotrectinib
		Pexidartinib
		Capmatinib
		Avapritinib
		Ripretinib
		Selpercatinib
		Pralsertinib
		Umbralisib
		Capivasertib
		Repotrectinib

* Incorporated but without degradation activity

### ATC L01EA – BCR-ABL tyrosine kinase inhibitors

**Figure 3.**
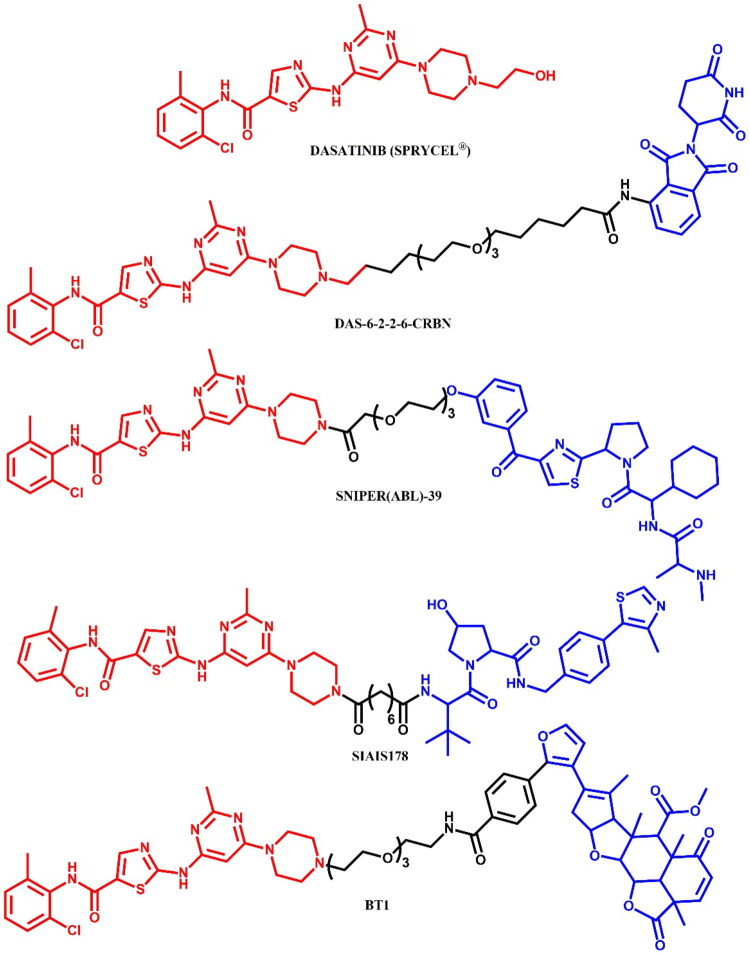
Structure of dasatinib and dasatinib-based PROTACs. Part I (the figure was drawn by the authors using Chemdraw software).

**Figure 4.**
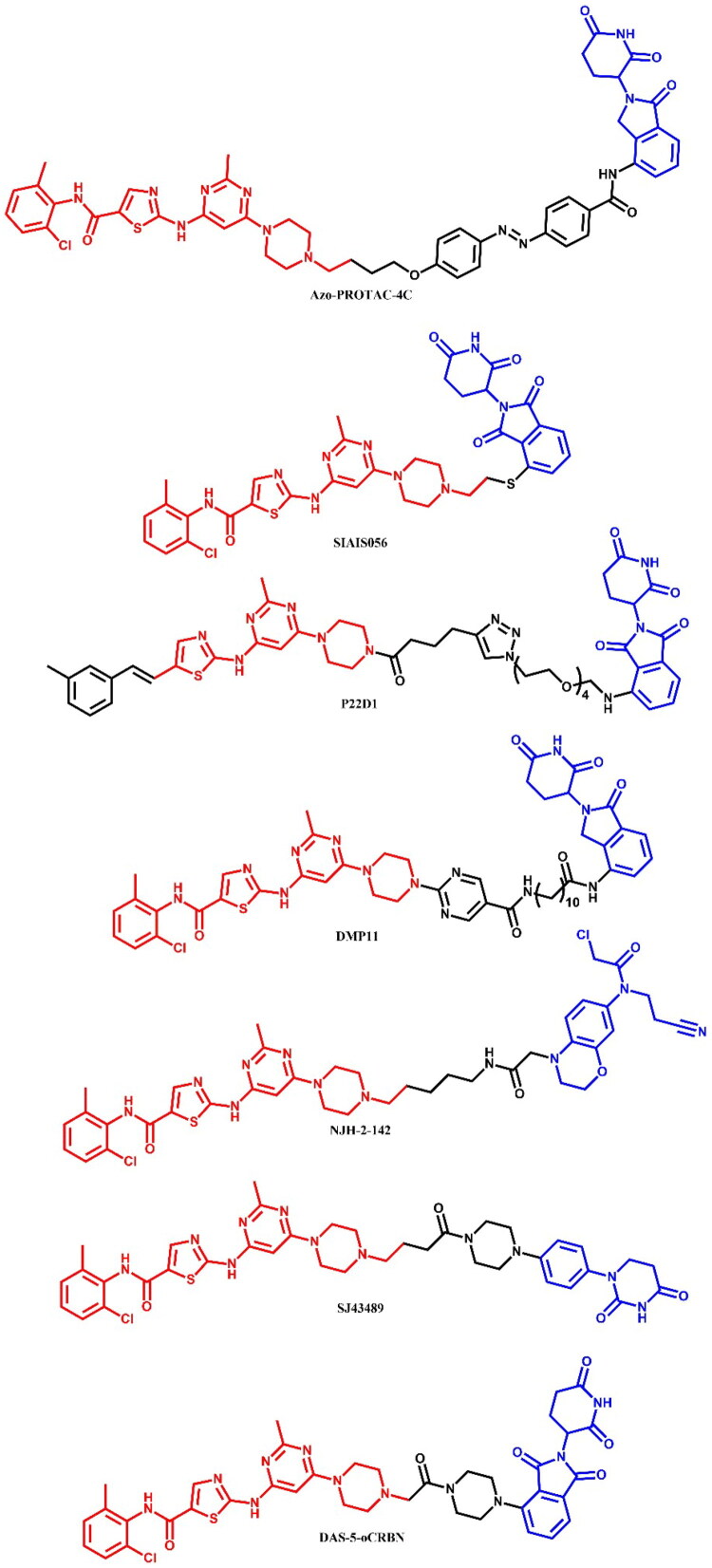
Structure of dasatinib and dasatinib-based PROTACs. Part II (the figure was drawn by the authors using Chemdraw software).

BCR-ABL tyrosine kinase inhibitors are a class of drugs targeting the abnormal BCR-ABL kinase, an oncogenic protein resulting from a chromosomal translocation that forms the Philadelphia chromosome (Ph+)^88^. This genetic rearrangement fuses the *BCR* (breakpoint cluster region) gene on chromosome 22 with the *ABL1* gene (Abelson tyrosine kinase) on chromosome 9, producing the BCR-ABL fusion protein. This fusion protein disrupts normal cellular signalling, leading to cell proliferation and, consequently, haematological malignancies, particularly chronic myeloid leukaemia (CML) and acute lymphoblastic leukaemia (ALL). One of the critical challenges in BCR-ABL-targeted therapy is the emergence of mutations, which confer resistance to tyrosine kinase inhibitors (TKIs). The most clinically significant mutation is T315I, often referred to as the “gatekeeper mutation”^89^. This mutation involves the substitution of threonine (T) with isoleucine (I) at position 315, resulting in steric clashes that block access of most TKIs to the binding pocket. Other common resistance mutations include G250E, E255V, V299L, F317L, and F317V. BCR-ABL inhibitors are essential therapy for Ph + leukaemia which helps to control or eradicate leukemic cells. In accordance with the established criteria, these inhibitors can be classified into three generations or four types, depending on the criteria^88^^,^^90^. The first-generation inhibitor imatinib was the initial treatment breakthrough. Second-generation inhibitors, such as asciminib, bosutinib, dasatinib, and nilotinib, were developed to address imatinib-resistant mutations and enhance activity. A third-generation inhibitor, ponatinib, was designed to especially overcome T315I mutation. Whereas, the types of inhibitors depend of binding sites available in BCR-ABL protein. All these drugs, except nilotinib, have been adapted into PROTAC technology to degrade BCR-ABL fusion protein (Figures 3, 4, 8, 10, and 12).

#### Dasatinib-based PROTACs

##### Rational design and development of dasatinib-based PROTACs

In 2016, Lai et al.^26^ developed the first PROTACs targeting BCR-ABL by linking TKIs (imatinib, bosutinib, dasatinib) to VHL or CRBN ligands. Using crystal structures (bosutinib – PDB: 3UE4; dasatinib – PDB: 2GQG), the piperazine moiety was chosen as the optimal linker attachment site. Four PEG-alkyl linkers were tested to optimise physicochemical properties. While all PROTACs retained low nanomolar binding to non-phosphorylated c-ABL, imatinib- and bosutinib-based PROTACs failed to induce degradation in K562 cells. In contrast, dasatinib-CRBN PROTACs degraded c-ABL (>85%) and BCR-ABL (>60%) at 1 μM. Replacing VHL with CRBN in bosutinib PROTACs yielded similar degradation efficiencies (>90% c-ABL, >80% BCR-ABL at 2.5 μM). The most potent compound, **DAS-6–2-2–6-CRBN** (Figure 3), showed >1000-fold selectivity for BCR-ABL-positive cells compared to non-BCR-ABL HEK293T and SK-BR-3 cell lines.

Shibata et al.^27^ investigated various combinations of ABL inhibitors and IAP ligands, leading to the development of **SNIPER(ABL)-39** (Figure 3), in which dasatinib was conjugated to an IAP ligand LCL161 derivative via a PEG3 linker. **SNIPER(ABL)-39** effectively degraded the BCR-ABL protein, inhibited the phosphorylation of STAT5 and CRKL, and suppressed the growth of BCR-ABL-positive CML cells. **SNIPER(ABL)-39** selectively degraded BCR-ABL (DC_50_ = 10.0 nM) without impacting off-targets proteins like cyclin B1 or MCL-1. Degradation of BCR-ABL required the ubiquitin-proteasome system, as confirmed by treatment with the ubiquitin-activating enzyme inhibitor MLN7243, which inhibited this process. shRNA-mediated gene silencing experiments indicated that the degradation of BCR-ABL required both XIAP and cIAP1, as simultaneous silencing of these proteins significantly suppressed BCR-ABL degradation, while targeting each individually had no effect. **SNIPER(ABL)-39** showed growth-inhibitory and protein knockdown effects in BCR-ABL-positive CML cell lines, such as KCL-22 (IC_50_ = 8.06 nM) and KU-812 (IC_50_ = 6.72 nM), but did not impact the growth of BCR-ABL-negative leukaemia lines like HL-60, MOLT-4, and Jurkat. Furthermore, **SNIPER(ABL)-39** was ineffective against SK-9 cells expressing the T315I mutant BCR-ABL protein, highlighting its specificity for wild-type BCR-ABL.

Zhao et al.^29^ evaluated PROTACs targeting BCR-ABL by connecting dasatinib with a VHL E3 ubiquitin ligase ligand, focusing on optimising linker length and composition. Through this optimisation, they developed **SIAIS178** (Figure 3), leading to effective BCR-ABL degradation (DC_50_ = 8.5 nM) with significant growth inhibition in BCR-ABL-positive leukemic cells (IC_50_ = 24.0 nM; for dasatinib: IC_50_ = 0.9 nM; for **DAS-6–2-2–6-CRBN**: 3.4 nM) *in vitro* and tumour regression in K562 xenograft models *in vivo*. Notably, **SIAIS178** also degraded clinically relevant BCR-ABL mutations associated with resistance (G250E, V299L, F317L, and F317V) in a dose-dependent manner, provided more durable suppression of CML cell growth than ABL kinase inhibitors. **SIAIS178** demonstrated selective antiproliferative activity against BCR-ABL CML cells, but not in BCR-ABL negative leukaemia lines such as U937, HL-60, or HEK293 cells, highlighting its selectivity. Kinome scan profiling confirmed that **SIAIS178** had fewer off-target interactions compared to dasatinib, and pharmacokinetic (PK) studies demonstrated favourable properties, with maximum plasma concentrations sufficient to match its *in vitro* activity.

Tong et al.^30^ discovered the natural product nimbolide, a covalent recruiter for the E3 ligase RNF114, which linked to dasatinib enabled selective degradation of oncogenic BCR-ABL fusion protein over normal c-ABL in leukaemia cells. The researchers synthesised two nimbolide-based PROTACs - **BT1** (Figure 3) with a longer PEG linker and a more substantial degradation effect than BT2 with a shorter alkyl linker. The improved performance of **BT1** was attributed to the enhanced formation of a ternary complex or increased accessibility within the cells. Interestingly, nimbolide led to the accumulation of RNF114 substrates, such as tumour suppressors p21 and p57, which could demonstrate additional anti-tumour effects. Similarly, **BT1** treatment also raised p21 levels in K562 cells, unlike dasatinib or reported CRBN-dasatinib and VHL-dasatinib PROTACs, indicating that nimbolide-based BCR-ABL degraders might have additional therapeutic benefits beyond those provided by dasatinib alone.

Jin et al.^31^ developed light-responsive Azo-PROTACs, which use azobenzene moieties as photoswitchable elements between the E3 ligase ligand and POI ligand. The photoswitchable moiety allowed light-controlled modulation of the PROTAC conformation, effectively switching the active state and reversibly controlling cell protein degradation. The authors found that the trans-configuration allowed dasatinib conjugation without steric hindrance, facilitating effective binding and degradation, whereas the cis-configuration showed significant hindrance and thus reduced degradation capacity. **Azo-PROTAC-4C** (Figure 4) proved to be the most effective in degrading the BCR-ABL fusion protein (IC_50_ = 68.0 nM). Its photoisomerisation properties were analysed using UV-visible absorption spectroscopy. In cell studies, the 4 C-trans conformation effectively degraded BCR-ABL and c-ABL proteins in BCR-ABL-positive K562 cells, with minimal impact on non-BCR-ABL cell lines. In contrast, the 4 C-cis form did not induce significant degradation under the same conditions, emphasising the critical functional difference between these two conformations. To test the reversibility of protein knockdown, the researchers exposed K562 cells treated with 4 C-trans to cycles of UV-C light and white light. In cells subjected to UV-C light, BCR-ABL and c-ABL protein levels gradually increased, indicating a loss of degradation activity as **Azo-PROTAC-4C** converted back to the cis form. Conversely, cells exposed to white light retained low BCR-ABL levels, maintaining degradation. This study demonstrated the reversible control of the protein degradation activity of **Azo-PROTAC-4C** via light-induced conformational changes.

Liu et al.^32^ explored BCR-ABL degradation using CRBN-recruiting dasatinib-based PROTAC, **SIAIS056** (Figure 4), with a sulphur-substituted carbon chain linker as the most potent *in vitro* degrader, with favourable pharmacokinetic properties *in vivo*. **SIAIS056** effectively degraded various clinically significant resistance BCR-ABL (DC_50_ = 0.18 nM; for **DAS-6–2-2–6-CRBN**: DC_50_ = 30.5 nM) mutations and demonstrated strong tumour regression in K562 xenograft models (IC_50_ = 0.49 nM; for dasatinib: IC_50_ = 0.9 nM; for **DAS-6–2-2–6-CRBN**: 4.1 nM). Structure-activity relationship (SAR) analysis revealed that linker length was not a critical factor in BCR-ABL degradation. Instead, alkylated linkers connecting dasatinib with CRBN ligands (such as pomalidomide or lenalidomide) showed superior degradation activity over acetylated linkers. In addition, sulphur-substitution at the C-4 position of pomalidomide enhanced both proliferation inhibition and BCR-ABL degrading activity. **SIAIS056** reduced BCR-ABL and c-ABL protein levels and inhibited the phosphorylation of BCR-ABL and its downstream effectors STAT5 and CRKL. **SIAIS056**, similar to **SIAIS178**^29^, also induced degradation of various BCR-ABL mutations (e.g. G250E, E255V, V299L, F317L, F317V, and T315A) at low nanomolar concentrations. However, as expected, the T315I mutation, which sterically hinders direct binding with imatinib and dasatinib, was resistant to degradation by **SIAIS056**. The study highlighted the strong *in vivo* efficacy and safety of **SIAIS056**, with continuous daily treatment resulting in sustained BCR-ABL protein degradation and phosphorylation suppression. Comparing **SIAIS056** to the VHL-based **SIAIS178**^29^ containing alkyl chain linkers showed that alkylated linkers could be optimal for future dasatinib-based PROTAC designs. Moreover, **SIAIS056** exhibited stronger *in vitro* anti-proliferative effects and broader BCR-ABL degradation than **SIAIS178**, suggesting that CRBN-recruiting PROTACs may be more effective across a wider range of BCR-ABL targets.

Yang et al.^33^ developed a novel series of dasatinib-, ponatinib-, and asciminib-based PROTACs that targeted all three binding sites on BCR-ABL. The main goal was to provide a PROTAC toolbox for the degradation of both wild-type and mutant BCR-ABL^T315I^ from each binding site. The dasatinib-based PROTAC P22D demonstrated degradation potency comparable to previously reported dasatinib-based PROTACs, **DA-6–2-2–6-CRBN**^26^ and **SIAIS178**^29^, though it was ineffective against the T315I mutation. To overcome this problem, the researchers designed **P22D1** (Figure 4) with a hydrophobic methylbenzene moiety and an alkyne group instead of a benzene moiety with an amide bond within the dasatinib structure, which enhanced hydrophobic interactions and reduced steric hindrance. This modification allowed **P22D1** to target the T315I mutation effectively, with a 0.57 μM IC_50_ value in BaF3-BCR-ABL (T315I) cells. While P22D and **DA-6–2-2–6-CRBN** failed to degrade mutant BCR-ABL^T315I^, **P22D1** significantly reduced the T315I protein levels, highlighting its specificity for this mutation with minimal off-target effects.

Zhang et al.^34^ designed and synthesised four BCR-ABL PROTACs featuring novel linkers containing a pyrimidine ring. Among them, **DMP11** (Figure 4), with a 10-carbon linker, was identified as the lead compound. **DMP11** significantly inhibited the activity of K562 cells (IC_50_ = 0.261 nM; for dasatinib: IC_50_ = 0.027 nM) and imatinib-resistant KA cells (IC_50_ = 0.837 nM; for dasatinib: IC_50_ = 6.36 nM). In both wild-type and imatinib-resistant CML cell lines, **DMP11** degraded the target protein BCR-ABL as well as SRC family proteins (Yes/Fyn/Fgr) in a time- and dose-dependent manner.

Henning et al.^35^ developed a cysteine-reactive covalent ligand, EN106, which targeted FEM1B, an E3 ligase that has been identified as a critical component in the cellular response to reductive stress. The PROTAC **NJH-2–142** (Figure 4), which was based on EN106 and linked via an alkyl linker to dasatinib, demonstrated BCR-ABL degradation in K562 CML cell lines. Nevertheless, further assessments of **NJH-2–142** were not reported, as the study’s primary objective was the discovery and development of FEM1B ligands.

Jarusiewicz et al.^36^ reported an alternative cereblon binder, phenyl dihydrouracil (PD), with improved chemical stability and enhanced protein degradation efficacy compared to thalidomide analogues. The authors demonstrated these improvements by linking PD to dasatinib to target LCK degradation, a common vulnerability in T-cell ALL. The most potent compound, **SJ43489** (Figure 4), exhibited a DC_50_ value of 0.8 nM, surpassing the corresponding phenyl glutarimide (PG) analogue (DC_50_ = 8 nM). Additionally, **SJ43489** showed the highest amplitude in the LCK-CRBN AlphaLISA assay, suggesting robust ternary complex stabilisation. This phenomenon rationalised its superior LCK degradation despite its relatively lower CRBN binding affinity (IC_50_ = 217.3 nM). Furthermore, **SJ43489** did not influence the levels of conventional IMiD neosubstrate proteins such as GSPT1, IKZF1, and CK1α. However, it exhibited a typical PROTAC profile, characterised by low cell permeability and high metabolic clearance.

Mao et al.^28^ identified a potent and selective dual CSK/c-SRC dasatinib-based PROTAC, **DAS-5-oCRBN** (Figure 4). This compound achieved 78% degradation of c-SRC at 100 nM in a ubiquitin- and proteasome-dependent manner in CAL-148 cells, while exhibiting no degradation of BCR-ABL in KCL-22 cell lines. The reverse-phase protein array (RPPA) revealed that **DAS-5-oCRBN** selectively (>50% degradation) targeted only CSK, a known off-target of dasatinib, and c-SRC among 394 cancer-related proteins. Additionally, the authors demonstrated that the geometry between the E3 ligase ligand and dasatinib, modified via 4- and 5-amino thalidomide derivatives, significantly impacted selectivity for c-SRC over BCR-ABL.

##### Synthesis of dasatinib-based PROTACs

**Figure 5.**
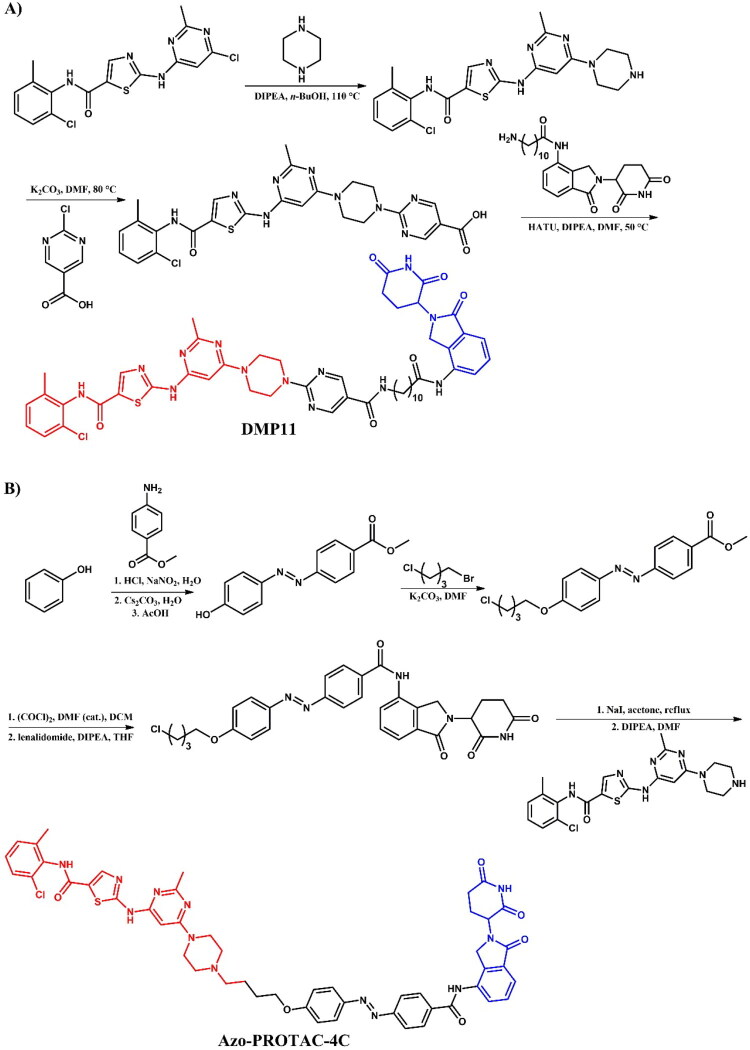
(a, b) Routes of synthesis of dasatinib-based PROTACs. Part I (the figure was drawn by the authors using Chemdraw software).

**Figure 6.**
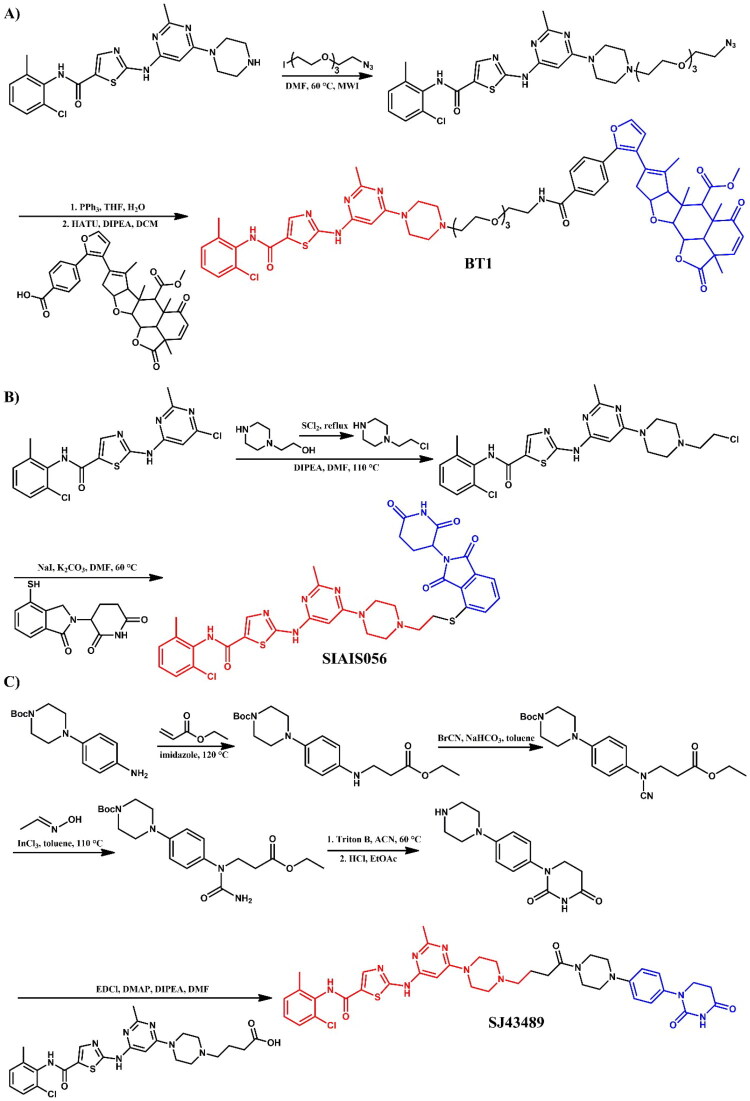
(a-c) Routes of synthesis of dasatinib-based PROTACs. Part II (the figure was drawn by the authors using Chemdraw software).

**Figure 7.**
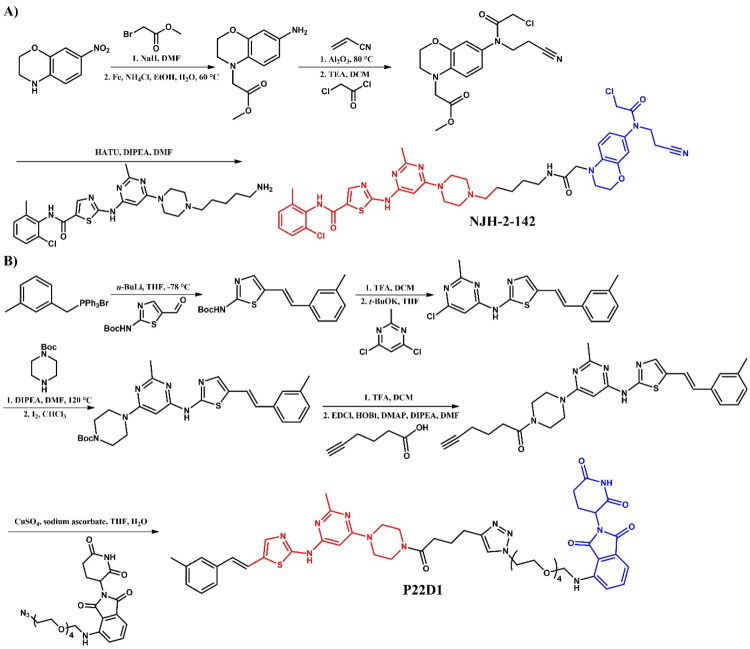
(a, b) Routes of synthesis of dasatinib-based PROTACs. Part III (the figure was drawn by the authors using Chemdraw software).

Each dasatinib-based PROTAC required a distinct synthetic pathway, necessitating tailored synthetic strategies to accommodate the structural differences between compounds (Figures 5–7). Depending on the design approach, these variations involved different methods for dasatinib functionalisation, linker selection, and conjugation with E3 ligase ligands.

While some syntheses employed a chlorinated dasatinib precursor undergoing nucleophilic aromatic substitution (S_N_Ar) with piperazine or related amines, in most cases a pre-functionalised dasatinib derivative bearing a free piperazine moiety was used. These intermediates were subsequently coupled with various linker–E3 ligand molecules via diverse chemical transformations. For instance, **DMP11** was obtained through sequential S_N_Ar and amide coupling steps (Figure 5a), whereas **P22D1** involved Cu(I)-catalysed azide–alkyne cycloaddition (“click chemistry”) (Figure 7b). In **SIAIS056**, a thioether linkage was introduced by nucleophilic substitution between the dasatinib intermediate and a thiol-functionalised cereblon ligand (Figure 6b). Notably, **Azo-PROTAC-4C** required a dedicated synthetic route for constructing the azobenzene linker fragment (Figure 5b). This involved the generation of a diazonium salt and its electrophilic coupling with phenol, followed by O-alkylation and stepwise assembly prior to final conjugation with dasatinib and the cereblon ligand. In contrast, **SJ43489** (Figure 6c)**, BT1** (Figure 6a) and **NJH-2–142** (Figure 7a) employed a non-canonical E3 ligase ligands, necessitating the multi-step synthesis or, in case of nimbolide (**BT1**), isolation from natural products, which were then coupled with a modified dasatinib derivative. Four additional PROTACs - **DAS-6–2-2–6-CRBN**, **SNIPER(ABL)-39**, **SIAIS178**, and **DAS-5-oCRBN** - were omitted from this synthetic overview. Their syntheses were either described only in the final step or closely resembled other synthetic pathways presented in this review and thus were not included to avoid duplication.

#### Asciminib-based PROTACs

##### Rational design and development of asciminib-based PROTACs

**Figure 8.**
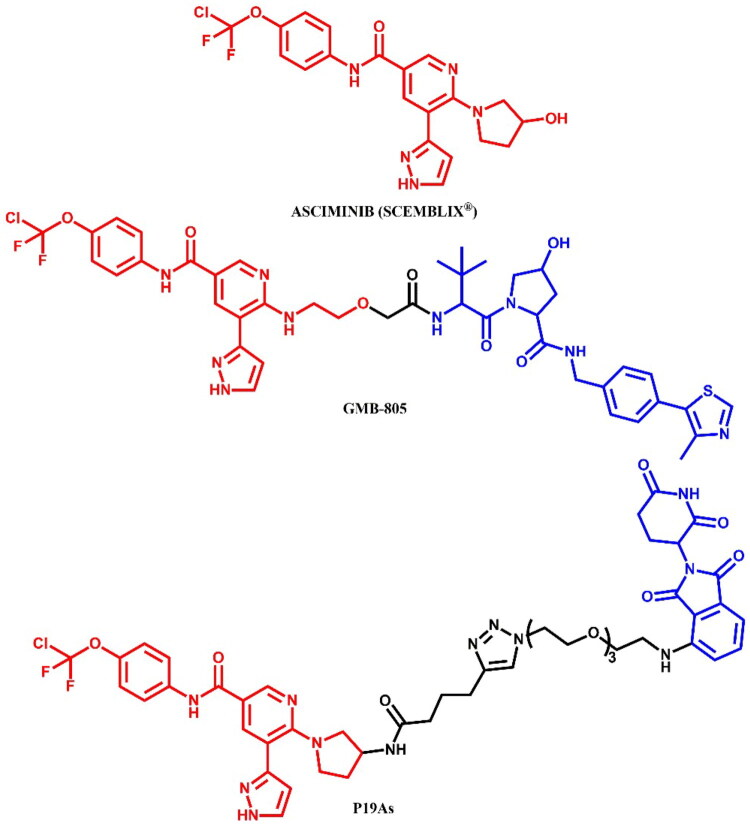
Structure of asciminib and asciminib-based PROTACs (the figure was drawn by the authors using Chemdraw software).

Burslem et al.^37^ developed **GMB-805** (Figure 8), an asciminib-based PROTAC designed using a scaffold-hopping approach to target BCR-ABL degradation. By replacing the GNF-5-derived moiety in their earlier compound GMB-475 with asciminib, the authors enhanced the degradation potency of this compound. **GMB-805** demonstrated over a 10-fold improvement in BCR-ABL degradation efficacy compared to GMB-475, achieving a DC_50_ of 30 nM in K562 cells. **GMB-805** exhibited potent antiproliferative activity with an IC_50_ of 169 nM in BCR-ABL-driven K562 cells. In contrast, no significant effects were observed in the control compound up to 1 μM. The *in vivo* efficacy of **GMB-805** was demonstrated in a K562 xenograft model, where daily intraperitoneal administration at 200 mg/kg effectively suppressed tumour growth over 6 days without observable toxicity.

Yang et al.^33^ described asciminib-based PROTACs targeting BCR-ABL. Among them, **P19As** (Figure 8) was identified as the most potent compound with moderate degrading activity, exhibiting a DC_50_ value of 200 nM in K562 cells. In comparison to dasatinib- and ponatinib-based PROTACs, **P19As** had reduced activity; however, it demonstrated enhanced activity relative to imatinib-based. The authors proposed that this phenomenon resulted from potential protein-protein crashes, particularly with the F-actin binding (FAMD) and SH2/3 domains of BCR-ABL, given the significant interaction of asciminib with these domains. Nevertheless, **P19As** decreased the protein level of BCR-ABL^T315I^ in BaF3 cells, though with a higher DC_50_ value than that observed in BCR-ABL^WT^. This was attributed to a reduction in binding affinity with the mutants.

##### Synthesis of asciminib-based PROTACs

**Figure 9.**
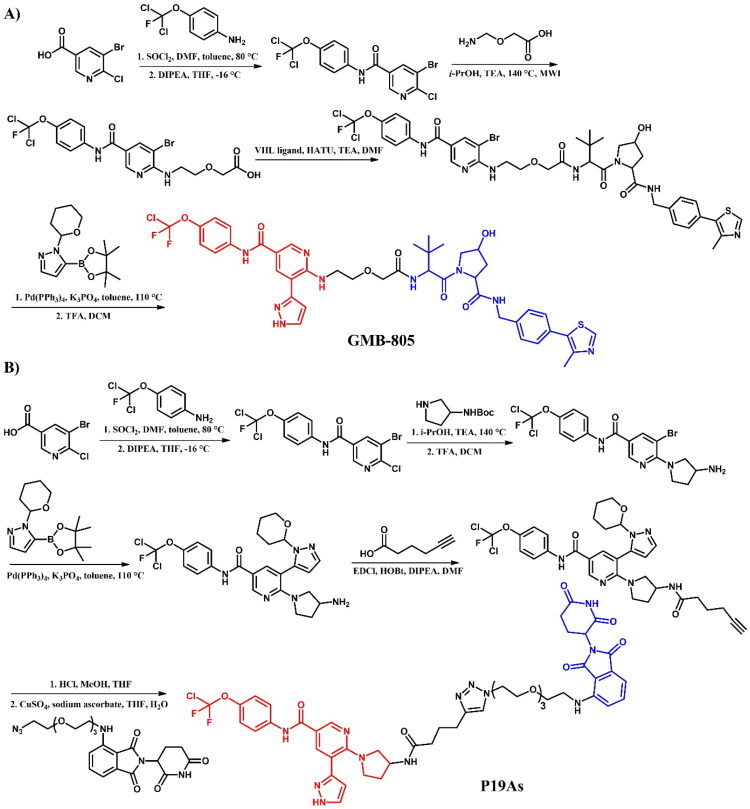
(a, b) Routes of synthesis of asciminib-based PROTACs (the figure was drawn by the authors using Chemdraw software).

5-bromo-6-chloronicotinic acid was used as the starting material in the initial stage of the preparation of both **GMB-805** (Figure 9a)^37^ and **P19As** (Figure 9b)^33^. This carboxylic acid was first converted to its corresponding acyl chloride using SOCl_2_, and then coupled with 4-(dichlorofluoromethoxy)aniline. The subsequent steps varied depending on the desired PROTAC. For **GMB-805**, the obtained compound underwent substitution with 2-(aminomethoxy)acetic acid using microwave-assisted irradiation synthesis (MWI) and was subsequently linked with a VHL ligand. The final step involved a Suzuki-Miyaura reaction with a THP-protected boronic acid pinacol ester derivative of substituted 1H-pyrazole. The THP protective group was then removed under acidic conditions. In contrast, for **P19As**, the third step involved substitution with Boc-protected pyrrolidin-3-amine, followed by Boc deprotection. Similar to **GMB-805**, the THP-protected boronic acid pinacol ester derivative of substituted 1H-pyrazole was then coupled with the resulting intermediate. Subsequently, the product was linked to hex-5-ynoic acid, enabling it to participate in a click reaction with a PEG3-azide-modified pomalidomide derivative in the presence of sodium ascorbate and copper(II) sulphate. This step resulted in the formation of a triazole ring and final **P19As**.

#### Ponatinib-based PROTAC

##### Rational design and development of ponatinib-based PROTAC

**Figure 10.**
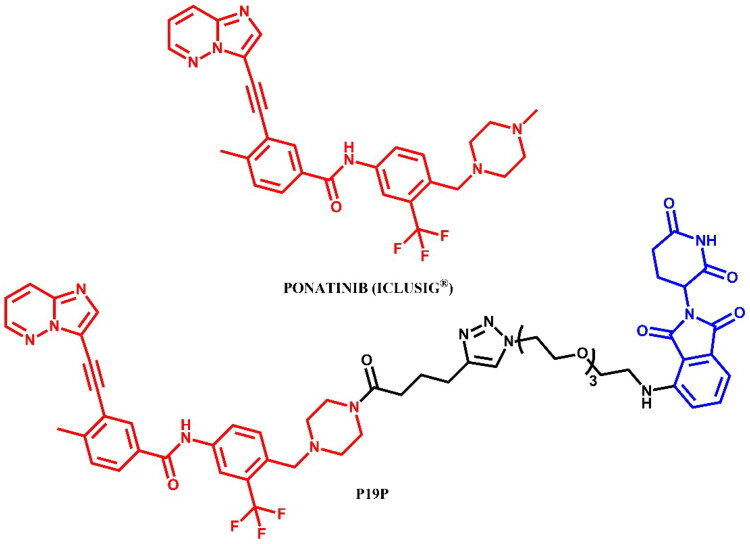
Structure of ponatinib and ponatinib-based PROTAC (the figure was drawn by the authors using Chemdraw software).

As previously stated (the Rational Design and Development of Dasatinib-Based PROTACs and the Synthesis of Dasatinib-Based PROTACs sections), among the ponatinib-based PROTACs described by Yang et al.^33^, **P19P** (Figure 10) was identified as the most potent compound. This PROTAC demonstrated high efficacy in the degradation of both wild-type and drug-resistant BCR-ABL mutants, including the challenging T315I mutant. The degradation was achieved with a DC_50_ of 20 nM in wild-type K562 cells and showed antiproliferative activity against T315I-mutant BCR-ABL-transformed BaF3 cells (IC_50_ = 13.1 nM; for ponatinib: IC_50_ = 1.0 nM). Furthermore, **P19P** effectively degraded other clinically significant mutations, such as E255K, H396R, and V468F, without significant off-target effects. In comparison to ponatinib, **P19P** exhibited reduced cytotoxicity in normal cells and mitigated adverse effects associated with VEGFR2-mediated vascular toxicity.

##### Synthesis of ponatinib-based PROTAC

**Figure 11.**
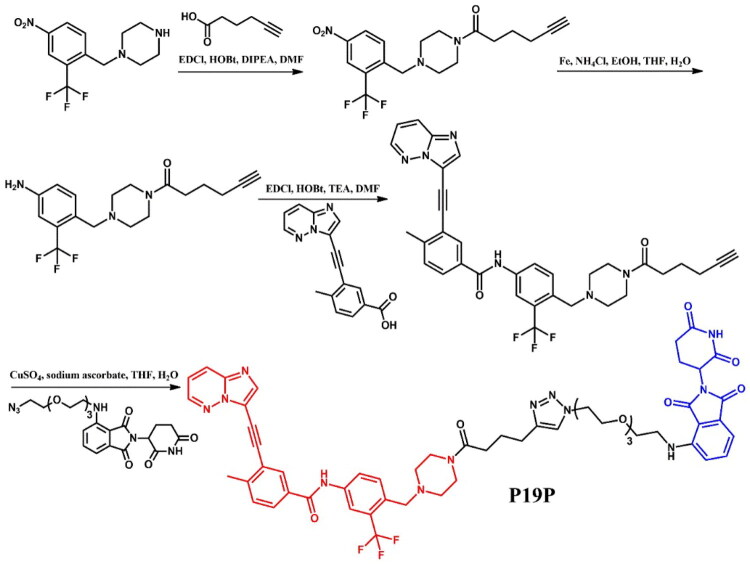
Routes of synthesis of ponatinib-based PROTACs (the figure was drawn by the authors using Chemdraw software).

The coupling reaction of the piperazine derivative with a nitro and a benzyl trifluoromethyl moiety, and hex-5-ynoic acid was the first step in obtaining the compound **P19P** (Figure 11)^33^. The nitro group in the resulting product was then reduced to an amine, enabling a subsequent coupling reaction with representative carboxylic acid, leading to a ponatinib derivative with a nitrile group. In the final step, this nitrile derivative underwent a click reaction with the same PEG3-azide-modified pomalidomide derivative used in the synthesis of **P19As**, resulting in the formation of a triazole ring, yielding P19P.

#### Bosutinib-based PROTAC

##### Rational design and development of bosutinib-based PROTAC

**Figure 12.**
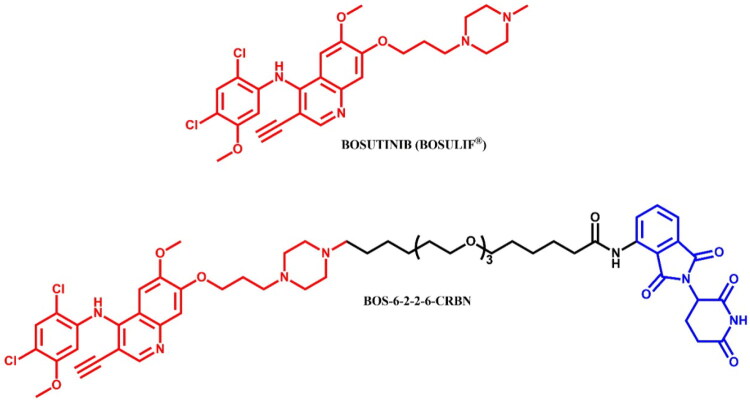
Structure of bosutinib and bosutinib-based PROTACs (the figure was drawn by the authors using Chemdraw software).

The bosutinib-based PROTACs described by Lai et al.^26^ were partially discussed in the Rational Design and Development of Dasatinib-Based PROTACs section. To summarise the information, despite target engagement, the bosutinib-VHL PROTAC was unable to degrade either c-ABL or BCR-ABL in K562 CML cells, potentially due to the insufficient orientation of the VHL ligase for efficient ubiquitination of BCR-ABL. Conversely, when bosutinib was conjugated to a CRBN ligand, the resulting bosutinib-CRBN PROTAC effectively degraded both BCR-ABL and c-ABL. In this regard, the most potent bosutinib-based PROTAC was **BOS-6–2-2–6-CRBN** (Figure 12) which induced over 90% degradation of c-ABL and 80% degradation of BCR-ABL at 2.5 μM.

The synthesis of bosutinib-based PROTAC was described only in its final step analogous to **DAS-6–2-2–6-CRBN** already detailed in the paper^26^. Consequently, we have chosen to omit this synthesis route from the graphical representation.

#### Imatinib-based PROTACs

##### Rational design and development of imatinib-based PROTACs

The imatinib-based PROTACs described by Yang et al.^33^, Lai et al.^26^, and Shibata et al.^27^ exhibited no degradation of BCR-ABL at high concentrations, despite the confirmation of target binding through the reduction of phosphorylation of downstream mediators, CRKL and STAT5. The authors collectively concluded that imatinib’s performance in the context of PROTACs was attributed to insufficient stabilisation of the ternary complex necessary for effective ubiquitination and degradation.

**Figure 13.**
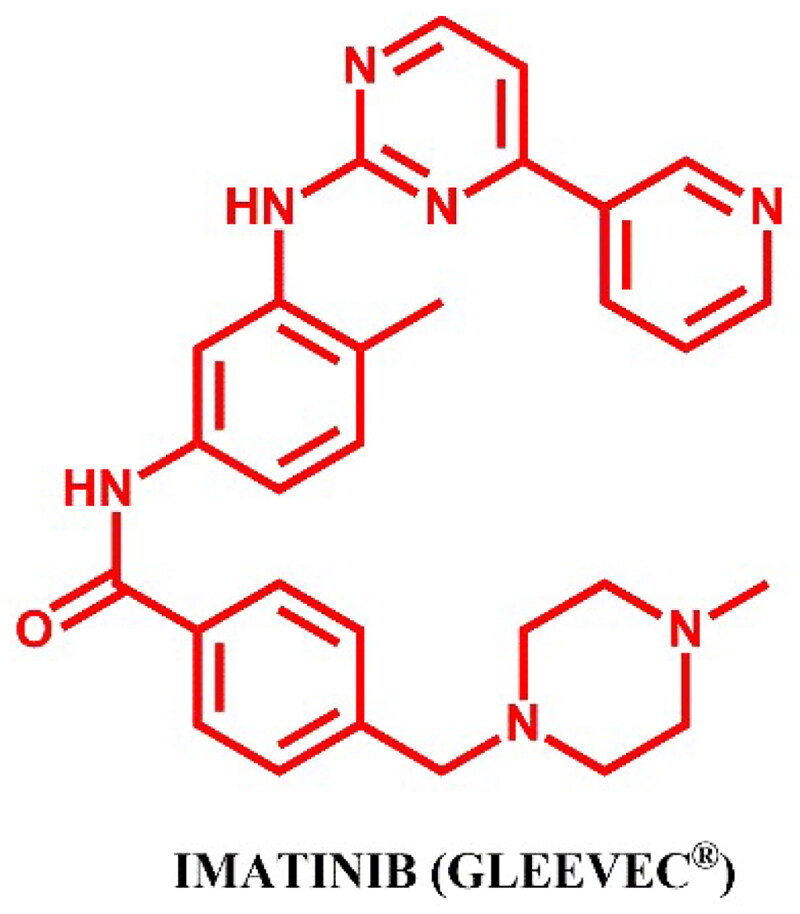
Structure of imatinib (the figure was drawn by the authors using Chemdraw software).

Therefore, Figure 13 presents only the structure of imatinib. The structures of imatinib-based PROTACs and their synthesis were omitted due to the absence of degradation activity against BCR-ABL.

### ATC L01EB – epidermal growth factor receptor (EGFR) tyrosine kinase inhibitors

**Figure 14.**
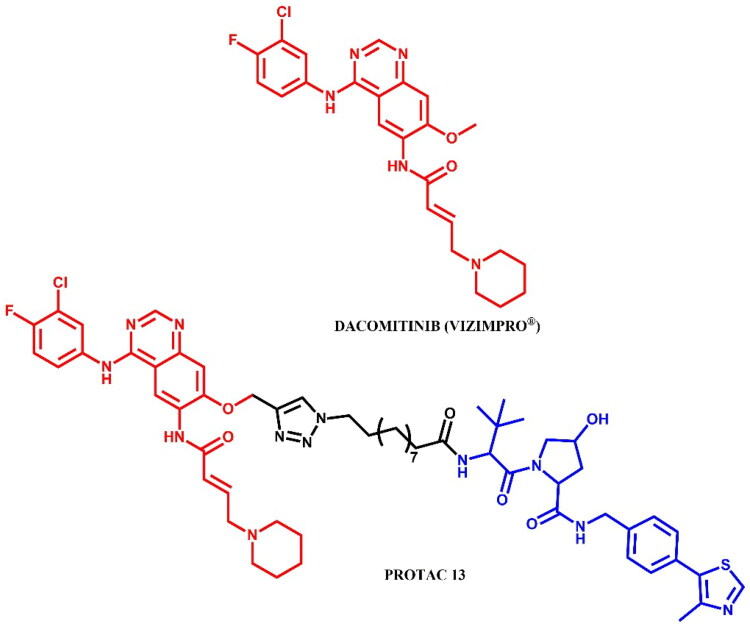
Structure of dacomitinib and dacomitinib-based PROTAC (the figure was drawn by the authors using Chemdraw software).

The epidermal growth factor receptor (EGFR), also known as HER-1 or ERBB-1, is a key receptor tyrosine kinase (RTK) that modulates various intracellular signalling pathways, especially in epithelial cells^91^. Upon epidermal growth factor (EGF) binding to its extracellular domain, EGFR dimerises and activates the intracellular tyrosine kinase domain. This activation, when constitutive, drives hyperactivity in downstream pathways such as RAS/RAF/MEK/ERK, PI3K/AKT/m-TOR, and STAT proteins, which collectively inhibit apoptosis while promoting cell survival, proliferation, and invasion. Overexpression of EGFRs is associated with poor prognosis in many cancers, including ovarian, bladder, gastric, head and neck, brain, breast, colon, and notably non-small-cell lung cancer^91^. The majority of EGFR mutations are found in exon 19 deletion (EGFR^del19^) or point mutations such as L858R (EGFR^L858R^) and T790M (EGFR^T790M^)^92^. First-generation EGFR inhibitors, like gefitinib, are widely used in clinical treatment of NSCLC^92^. However, resistance has led to the development of irreversible second-generation inhibitors, including dacomitinib and afatinib, which show greater selectivity and sensitivity for EGFR^T790M^ mutations. Despite these advances, their strong activity against wild-type EGFR (EGFR^WT^), often leads to side effects, driving the need for third-generation inhibitors, like osimertinib and rociletinib, which have greater precision in targeting. Each generation of EGFR inhibitors has been utilised in PROTAC technology (Figures 14, 16, 18, 20, and 21).

#### Dacomitinib-based PROTAC

##### Design and development of dacomitinib-based PROTAC

Shi et al.^38^ developed dacomitinib-based EGFR degraders, with **PROTAC 13** (Figure 14) showing potent degradation of EGFR^del19^ (DC_50_ = 3.57 nM; IC_50_ = 6 nM; for dacomitinib: IC_50_ = 7 nM) in HCC-827 cells, while displaying minimal activity against other EGFR mutants (e.g. L858R/T790M) and EGFR^WT^-expressing lines (A549, A431), as well as HER2/HER4 ((IC_50_ > 20 μM EGFR mutants and wild-type; for dacomitinib: IC_50_ = 700 nM; 1.79 μM; 1.96 μM; respectively). In HCC-827 xenograft models, **PROTAC 13** showed strong antitumor efficacy without toxicity, alongside favourable PK and microsomal stability. Docking studies (PDB ID: 4I24) identified a solvent-exposed methoxy group on dacomitinib suitable for linker attachment. Among 16 analogues, alkyl-linked VHL-based PROTACs showed superior antiproliferative activity**. PROTAC 13** reduced pAKT/pERK levels, induced cell cycle arrest and apoptosis (66% at 50 nM), and degraded EGFR^del19^ in a time- and UPS-dependent manner.

##### Synthesis of dacomitinib-based PROTAC

**Figure 15.**
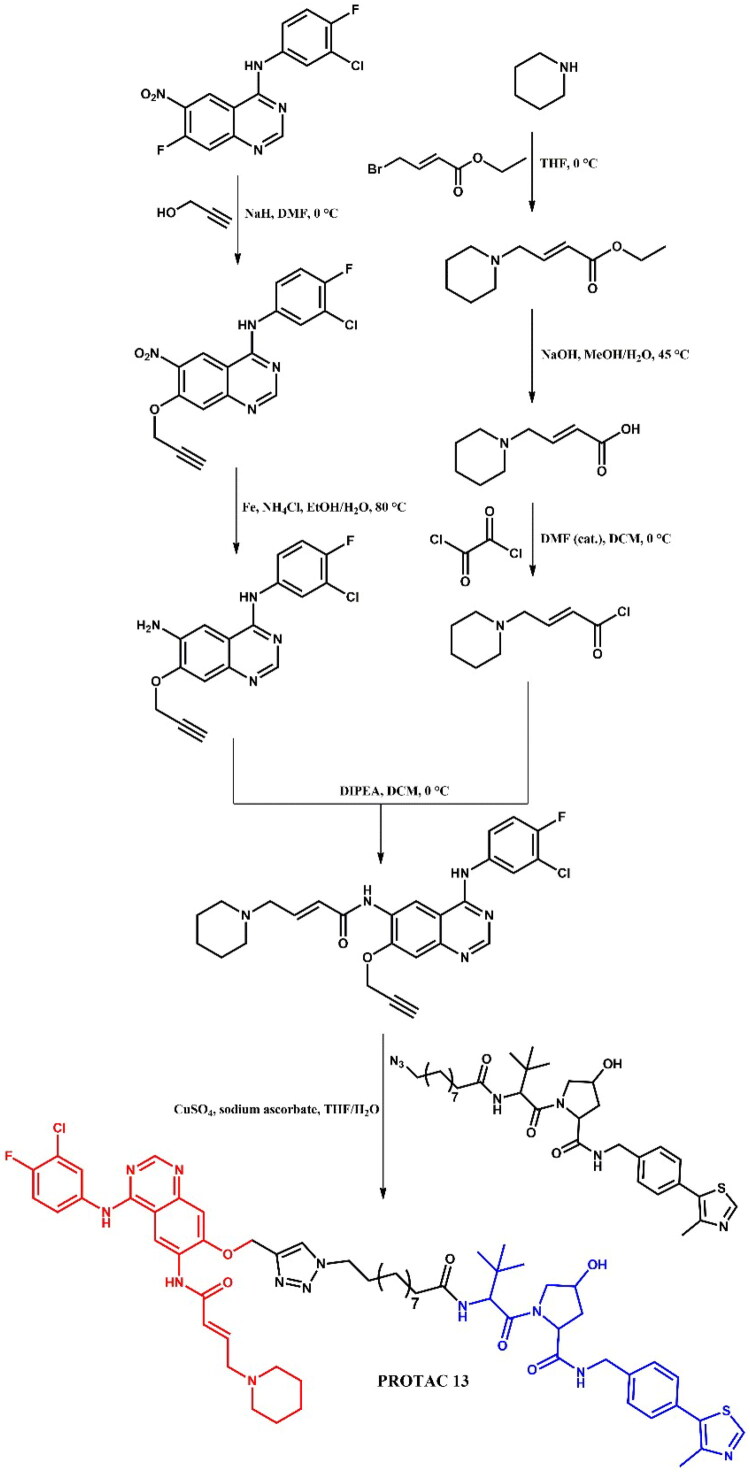
Routes of synthesis of dacomitinib-based PROTAC (the figure was drawn by the authors using Chemdraw software).

First, the alkyne derivative of dacomitinib was prepared to form a triazole linker with a suitable azide derivative of the VHL ligand in click chemistry (Figure 15). The core structure of dacomitinib, N-(3-chloro-4-fluorophenyl)-7-fluoro-6-nitroquinazolin-4-amine, underwent an S_N_Ar substitution with propargyl alcohol in the presence of sodium hydride. The nitro group of the resulting product was then reduced with iron powder and ammonium chloride to yield an amine group, resulting in a pyrimidine-derivative intermediate. Separately, piperidine reacted with ethyl 4-bromocrotonate in a substitution reaction, followed by hydrolysis under basic conditions and reaction with oxalyl chloride to produce the second intermediate - the reactive acyl chloride. Finally, these two intermediates were combined in an amidation reaction, and the obtained product was then coupled with the respective azide derivative of the VHL ligand in a copper-catalysed azide-alkyne cycloaddition to complete the synthesis.

#### Rociletinib-based PROTAC

##### Rational design and development of rociletinib-based PROTAC

**Figure 16.**
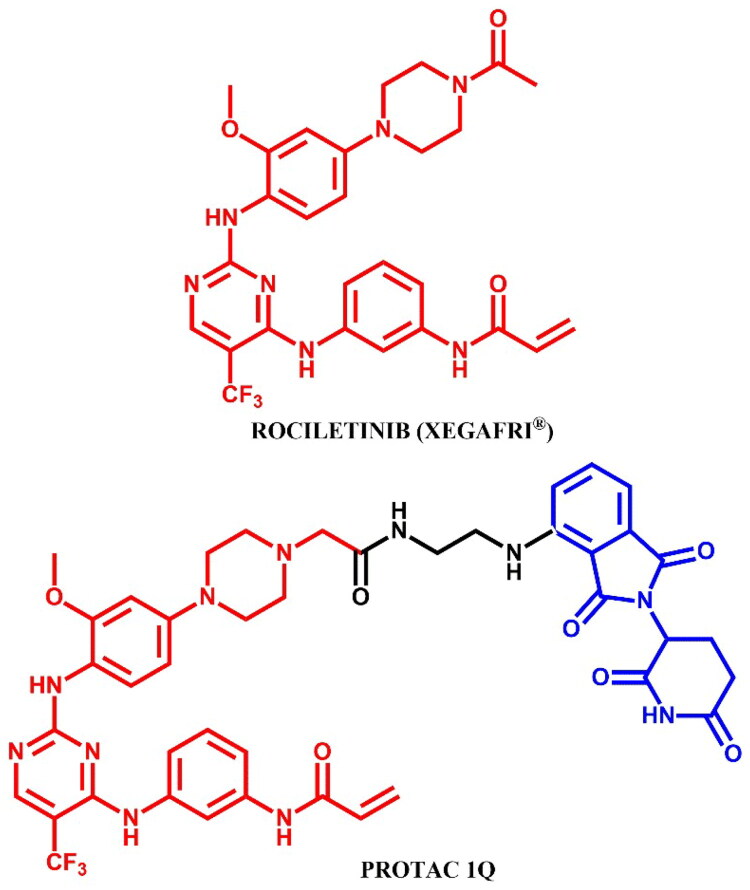
Structure of rociletinib and rociletinib-based PROTAC (the figure was drawn by the authors using Chemdraw software).

Li et al.^39^ reported the discovery of EGFR^L858R/T790M^ degraders based on rociletinib, emphasising **PROTAC 1Q** (Figure 16) as a potent and selective agent targeting EGFR^L858R/T790M^ in H1975 (IC_50_ = 0.75 μM; for CO-1686 (positive control): IC_50_ = 0.27 μM) and PC-9 (EGFR^del19^) cells (IC_50_ = 0.24 μM; for CO-1686: IC_50_ = 0.13 μM), while sparing EGFR^WT^ in A549 cells. **PROTAC 1Q** showed degradation activity in H1975 cells (DC_50_ = 0.36 μM), demonstrating both time- and concentration-dependent degradation of EGFR^L858R/T790M^. Pre-treatment with MG132 (proteasome inhibitor) and MLN4924 (ubiquitination inhibitor) fully blocked degradation, confirming the ubiquitin-proteasome system as the mediating pathway. To investigate the binding interactions, molecular docking on the co-crystal structure of rociletinib with EGFR^T790M^ (PDB ID: 5XDK) revealed that the *N*-acetylpiperazine moiety, exposed to solvent provided an optimal attachment point for linking an E3 ligase recruiting ligand via linkers such as PEGs, alkyl diamines, alkyls, and dicarboxylic acids with different lengths. Accordingly, **PROTAC 1Q** was designed by removing the N-acetyl group and attaching pomalidomide via a linker to the outer nitrogen of the piperazine group. This structural modification improved the selectivity of **PROTAC 1Q** for EGFR^L858R/T790M^ over EGFR^WT^ and led to effective apoptosis induction and arrest of H1975 cells in the G0/G1 phase.

##### Synthesis of rociletinib-based PROTACs

**Figure 17.**
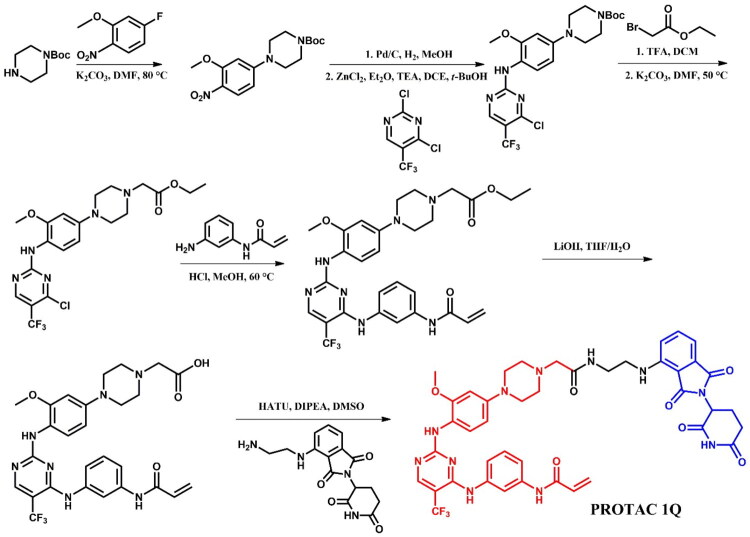
Routes of synthesis of rociletinib-based PROTAC (the figure was drawn by the authors using Chemdraw software).

The synthesis of **PROTAC 1Q** was started from S_N_Ar reaction of singly Boc-protected piperazine with 2-nitro-5-fluoroanisole (Figure 17). The nitro group was then reduced via catalytic hydrogenation, enabling another S_N_Ar reaction with 1,4-dichloro-5-trifluoromethylpyrimidine, facilitated by zinc chloride as a Lewis acid. Next, the Boc-protected nitrogen in the piperazine moiety was deprotected, exposing a reactive amine modified with an ethyl acetate group to function as a linker. Subsequently, the obtained product reacted with N-(3-aminophenyl)acrylamide in next S_N_Ar where the chloride on the pyrimidine was displaced, adding further functional components. This intermediate was hydrolysed under strong basic conditions to adjust its functional groups for the final coupling. In the last step, it was coupled with an ethylamine pomalidomide derivative, providing **PROTAC 1Q.**

#### Osimertinib-based PROTAC

##### Rational design and development of osimertinib-based PROTAC

**Figure 18.**
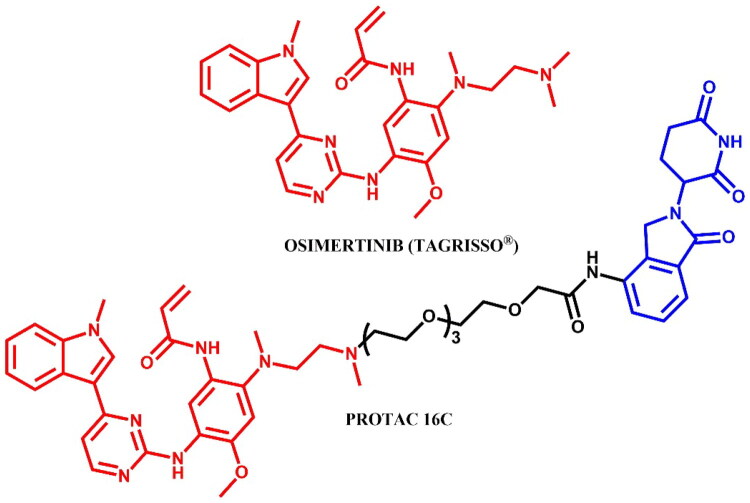
Structure of osimertinib and osimertinib-based PROTAC (the figure was drawn by the authors using Chemdraw software).

He et al.^40^ designed osimertinib-based PROTACs conjugated to lenalidomide, targeting EGFR. Among them, **PROTAC 16 C** (Figure 18) demonstrated strong antiproliferative activity in PC9 (IC_50_ = 0.413 μM), HCC827 (IC_50_ = 1.34 μM), and H1975 cells (IC_50_ = 0.657 μM), degrading EGFR by up to 68% in PC9 cells, and inducing apoptosis and G0/G1 arrest. Structural analysis (PDB ID: 3IKA) revealed a solvent-exposed dimethylamine on osimertinib as a suitable linker site. Mechanistic studies using MG-132 and lenalidomide confirmed that EGFR degradation by **PROTAC 16 C** was mediated via UPS.

##### Synthesis of osimertinib-based PROTACs

**Figure 19.**
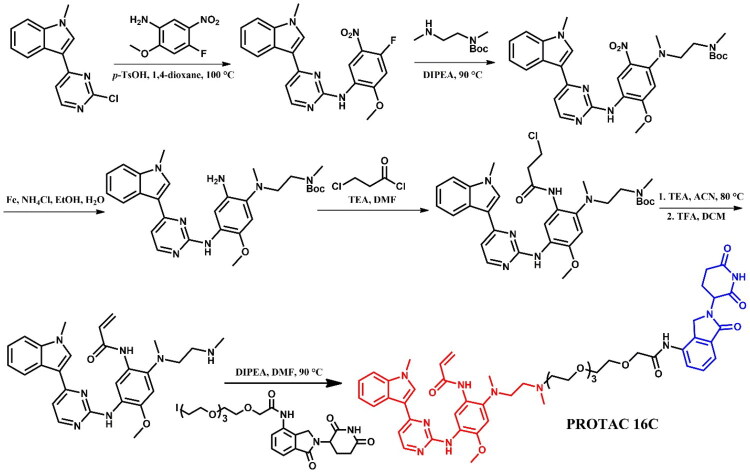
Routes of synthesis of osimertinib-based PROTAC (the figure was drawn by the authors using Chemdraw software).

The synthetic route of **PROTAC 16 C** was started from 3–(2-chloropyrimidin-4-yl)-1-methyl-1*H*-indole which underwent S_N_Ar reaction with 4-fluoro-2-methoxy-5-nitroaniline under acidic conditions in the presence of *p*-toluenesulphonic acid (Figure 19). Subsequently, resulting material underwent reaction with single Boc-protected *N*,*N*-dimethyl(ethylendiamine), followed by the reduction of nitro group to an amine group with treatment of iron powder and ammonium chloride. This was followed by amidation with 3-chloropropionyl chloride and then elimination of the HCl to produce an acrylamide derivative in the presence of triethylamine under reflux. Finally, the removal of the Boc group allowed for an alkylation reaction with the iodide derivative of lenalidomide.

#### Afatinib-based PROTAC

##### Rational design and development of afatinib-based PROTAC

**Figure 20.**
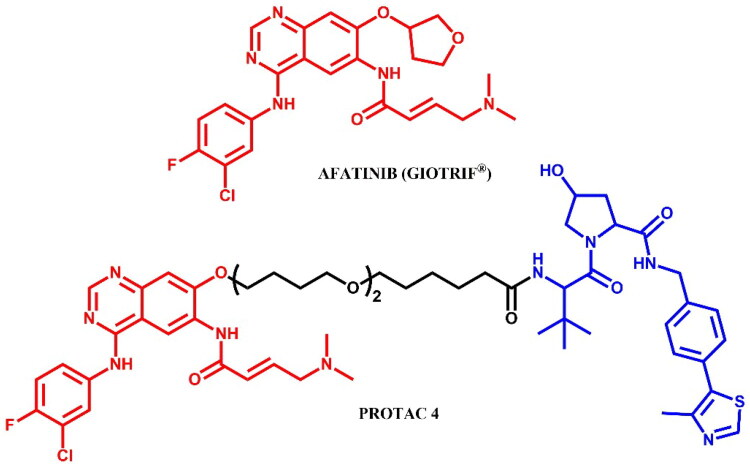
Structure of afatinib and afatinib-based PROTAC (the figure was drawn by the authors using Chemdraw software).

Burslem et al.^41^ highlighted the advantages of targeted protein degradation over traditional inhibition, referring to several EGFR kinase inhibitors, such as lapatinib, afatinib, and gefitinib. The authors developed afatinib-based **PROTAC 4** (Figure 20) with modified afatinib scaffold by removing the oxolane group, capable of degrading the gefitinib-resistant EGFR double mutant (EGFR^L858R/T790M^) in the H1975 cell line, with a maximum degradation of 79.1%. Further evaluations of **PROTAC 4** were not reported due to particular emphasis on certain compounds described by the authors as “lapatinib-based PROTACs”. However, according to our methodology, these compounds do not qualify as lapatinib-based PROTACs due to significant structural modifications to the lapatinib scaffold, which alter their classification within our framework.

The synthesis of afatinib-based PROTAC was described only in its final step - a coupling reaction - making it analogous to other methods already detailed in the paper. Consequently, we have chosen to omit this synthesis route from the graphical representation.

#### Gefitinib-based PROTACs

##### Rational design and development of gefitinib-based PROTACs

**Figure 21.**
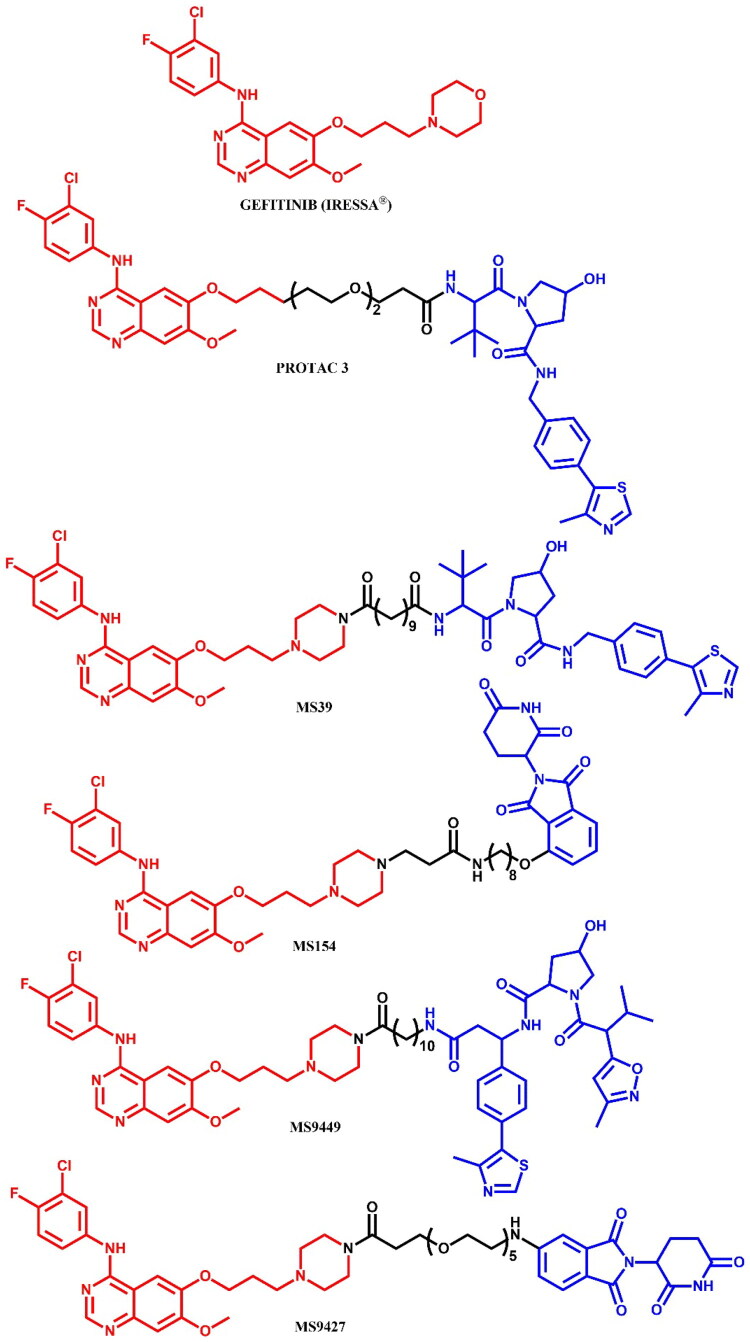
Structure of gefitinib and gefitinib-based PROTACs (the figure was drawn by the authors using Chemdraw software).

Burslem et al.^41^ also developed gefitinib-based **PROTAC 3** (Figure 21), which effectively degraded EGFR^del19^ in HCC827 cells and EGFR^L858R^ in H3255 cells, while sparing EGFR^WT^. **PROTAC 3** demonstrated nanomolar potency with maximum degradation rates of 98.9% and 96.6% in HCC827 and H3255 cells, respectively. Structural modifications involved the removal of the morpholine moiety from gefitinib, enabling linker attachment to the VHL ligand. **PROTAC 3** has not been studied further.

Cheng et al.^42^ reported the development of two gefitinib-based degraders, **MS39** (VHL-recruiting) and **MS154** (CRBN-recruiting) (Figure 21), that selectively target mutant EGFR while sparing EGFR^WT^. Mechanistic studies confirmed EGFR degradation via the respective E3 ligases, and proteomic analysis showed high specificity with over 75% EGFR degradation. Structural studies (PDB ID: 4I22) guided the transformation of gefitinib’s morpholine group into piperazine, enabling attachment to the E3 ligase ligand. **MS39** showed roughly 10-fold weaker binding to EGFR^WT^ and L858R mutant (K_d_ = 11 nM and 12 nM, respectively) than gefitinib (K_d_ = 1.1 nM and 0.8 nM, respectively), while **MS154** retained a high affinity for EGFR^WT^ (K_d_ = 1.8 nM) with fivefold weaker binding to the L858R mutant (K_d_ = 3.8 nM). Both **MS39** and **MS154** exhibited nanomolar DC_50_ values in HCC-827 (DC_50_ = 5.0 nM and DC_50_ = 11 nM, respectively) and H3255 cells (DC_50_ = 3.3 nM and DC_50_ = 25 nM, respectively), with a slight “hook effect”, referring to the phenomenon where higher concentrations of the PROTAC resulted in reduced potency due to the tendency to form binary complexes “PROTAC-POI” and “PROTAC-E3” rather than the ternary complex “POI-PROTAC-E3”. Both compounds effectively inhibited EGFR autophosphorylation and downstream AKT phosphorylation, but **MS39** showed slightly more potent inhibition in HCC-827 and H3255 cells. Comparison with the VHL-recruiting PROTAC 3^41^ revealed similar efficacy, but PROTAC 3 exhibited a more pronounced hook effect at high concentrations. **MS39** and **MS154** did not reduce EGFR^WT^ levels in OVCAR-8 and H1299 cells. Pharmacokinetic studies revealed high plasma levels and a longer half-life for **MS39**, making it suitable for *in vivo* EGFR degradation. **MS154** was also bioavailable, although at lower plasma levels than **MS39**. Both compounds showed reduced cell growth inhibition compared to gefitinib, likely due to lower cell permeability.

Yu et al.^43^ conducted detailed SAR studies that led to the discovery of two gefitinib-based degraders, **MS9449** (VHL-recruiting) and **MS9427** (CRBN-recruiting) (Figure 21), targeting mutant EGFR while sparing EGFR^WT^. The authors created negative control analogues to evaluate degrader specificity - a diastereomer of **MS9449** with reversed stereochemistry to disrupt VHL interactions and an IMiD-modified analogue to disrupt CRBN binding. Binding assays showed that **MS9449** and **MS9427** exhibited weaker affinities for EGFR^WT^ (K_d_ = 29 nM and 6.4 nM, respectively) and EGFR^L858R^ (K_d_ = 13 nM and 2.9 nM, respectively) compared to gefitinib (K_d_ = 1.1 nM and 0.8 nM, respectively). Both compounds effectively induced concentration-dependent degradation of the EGFR^Del19^ mutant and inhibited p-EGFR in HCC-827 cells, with **MS9449** showing approximately 10-fold greater potency than **MS9427**. Mechanistic studies showed that EGFR degradation by **MS9449** and **MS9427** was blocked by the NEDD8-activating enzyme inhibitor MLN-4924, indicating involvement of an active cullin-RING ligase (CRL) complex in ubiquitination process. Pre-treatment with MG-132, gefitinib, or negative controls reduced EGFR degradation. Surprisingly, autophagy-lysosome pathway involvement in EGFR degradation was demonstrated, as pre-treatment with autophagy/lysosome inhibitor BoA1 rescued degradation effects. However, the role of this pathway in the mechanism of action of these EGFR degraders remains unclear. **MS9449** and **MS9427** did not significantly affect EGFR^WT^ levels in OVCAR-8 cells expressing EGFR^WT^, despite their high binding affinity to this form, indicating that high binding affinity alone does not determine degradation selectivity. Combination treatment with the PI3K inhibitor pictilisib enhanced the anti-proliferative effects of **MS9449** and **MS9427** in both cell lines, highlighting the potential of combination therapies in cancer cells expressing EGFR^WT^.

##### Synthesis of gefitinib-based PROTACs

**Figure 22.**
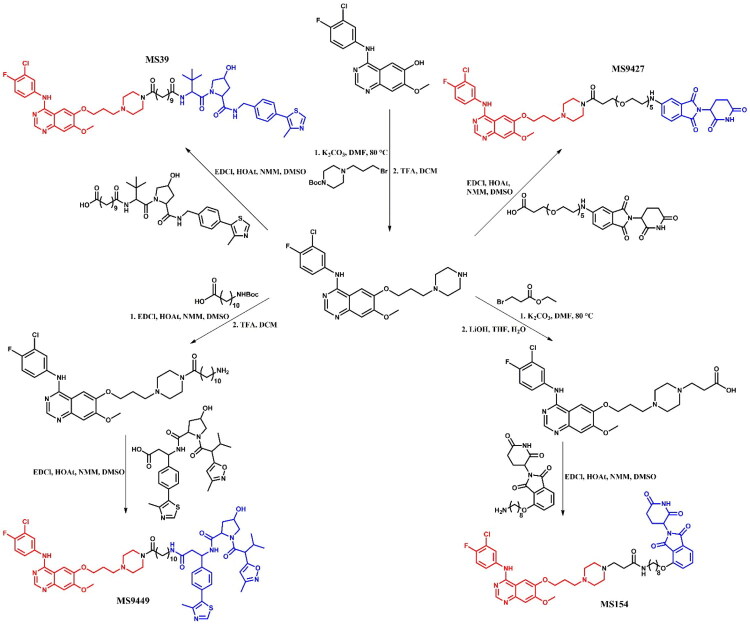
Routes of synthesis of gefitinib-based PROTACs (the figure was drawn by the authors using Chemdraw software).

All the gefitinib-based PROTACs mentioned were synthesised from a primary intermediate in which the morpholine group was changed into piperazine moiety (Figure 22). The PROTACs **MS39** and **MS9427** were obtained by coupling this intermediate with modified carboxylic acids of the VHL and the CRBN ligands, respectively. Meanwhile, **MS9449** was synthesised by an initial coupling reaction with a long-chain amino acid, followed by coupling with the VHL ligand. In contrast, **MS154** was synthesised by first substituting the piperazine moiety with an ethyl acetate group, followed by ester hydrolysis in the presence of a strong base and, finally, coupling with a CRBN ligand containing a long alkyl chain and an amine group. Each coupling reaction was carried out under the same conditions using 1-ethyl-3–(3-dimethylaminopropyl)carbodiimide hydrochloride (EDCl), 1-hydroxy-7-azabenzotriazole (HOAt), and *N*-methylmorpholine (NMM) in DMSO.

### ATC L01EC - BRAF kinase inhibitors (BRAFi)

**Figure 23.**
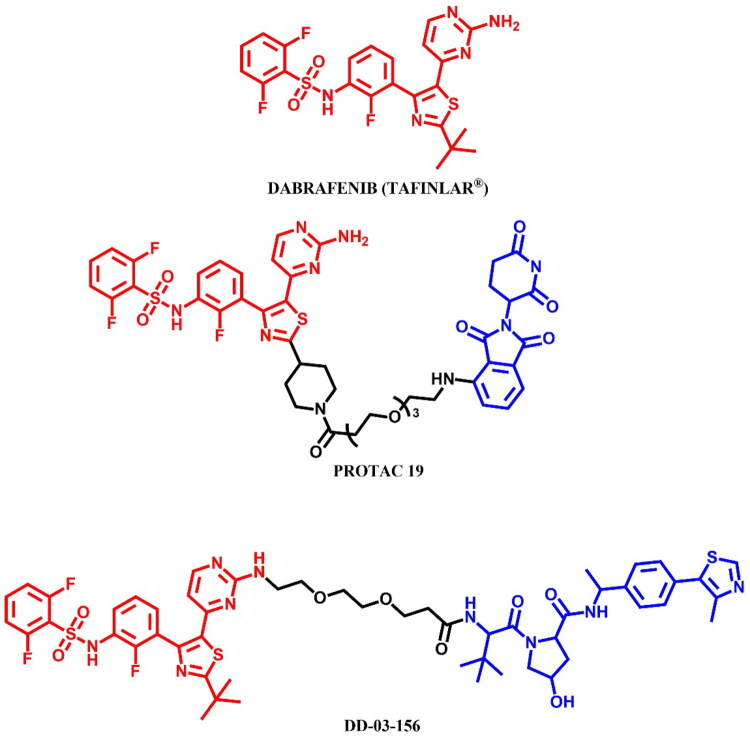
Structure of dabrafenib and dabrafenib-based PROTACs (the figure was drawn by the authors using Chemdraw software).

The RAF (Rapidly Accelerated Fibrosarcoma) family kinases function in the MAPK pathway is transmitting signals from activated RAS to the downstream kinases MEK and ERK^93^. This pathway regulates cell proliferation, differentiation, and survival, enabling mutations in RAS and RAF to act as potent drivers of human cancers. BRAF kinase is a member of the RAF kinase family and its oncogenic mutations lead to constitutive activation of the MAPK pathway. This mutations are frequently observed in specific cancers, including approximately 50% of melanoma^94^. Three classes of BRAF mutations have been described in the literature^93^^,^^95^. The first class includes V600E and V600K mutations which are hyperactivated and can transduce a signal as monomers in the absence of activated RAS. The second class consists of the RAS-independent dimers K601E and G469A. Lastly, the third class of BRAF mutants includes G466V and D594N which bind tightly to RAS and recruit CRAF into hyperactivated heterodimers. The most common BRAF mutation is V600E, which accounts for approximately 90% of BRAF mutations found in melanoma^94^. The FDA-approved BRAF inhibitors dabrafenib, vemurafenib, encorafenib, and binimetinib are used in the treatment of patients with BRAF-mutant melanoma by selectively targeting BRAF kinase and interfering MAPK signalling pathway. The first three drugs mentioned were used in PROTAC technology (Figures 23, 25, and 27).

#### Dabrafenib-based PROTACs

##### Rational design and development of dabrafenib-based PROTACs

Posternak et al.^44^ developed 16 different PROTACs, combining three BRAF ligands (two dabrafenib-based molecules and the preclinical inhibitor BI 882370) combined with two thalidomide derivatives and a VHL ligand. The authors employed flexible linkers (PEGs and alkyl) of variable length, which were attached to the dabrafenib in two different sites of the molecule. This approach was possible by functionalisation of dabrafenib by changing tert-butyl group into piperidine (first dabrafenib derivative) and attaching the linker directly to the free amine group (second derivative). Most candidate PROTACs behaved as potent inhibitors of BRAF^V600E^, indicating a successful design strategy for linker attachment, which did not perturb target engagement. The authors also synthesised negative E3-binding controls as N-methylated CRBN ligands at the nitrogen position of the glutamine ring for the most potent molecules. As the result, BI 882370-based PROTACs showed more effectiveness than dabrafenib-based. Nevertheless, the most potent dabrafenib-based molecule was **PROTAC 19** (Figure 23) (IC_50_ = 26.0 nM, D_max_ = 50%, DC_50_ = 500 nM in HEK293 cell line; IC_50_ = 105.0 nM in A375 cell line; for dabrafenib: IC_50_ = 13.0 nM in HEK293 cell line; IC_50_ = 619.0 nM in A375 cell line), a hybrid of the piperidine derivative dabrafenib linked to pomalidomide through PEG3.

Donovan et al.^45^, in their study, mapped the degradable kinome for accelerated PROTAC development. For this purpose, the authors combined a library of degraders to determine the degradability of more than 200 kinases in 7 different cell lines. As a result, the study found active degradation molecules for more than 16 understudied kinases, including CDK17 (cyclin-dependent kinase 17) for which the kinase ligand was dabrafenib as a part of PROTAC DD-03–156 (Figure 23) molecule consisting of a non-changed structure of dabrafenib, PEG2 as a linker and VHL ligand. This case illustrated how the additional constraints required for degradation can lead to altered selectivity in the degrader relative to the initial inhibitor. It also demonstrated how significant the benefit that informed scaffold selection can have on the identification of starting chemistry and PROTAC design. In addition, **DD-03–156** was identified as a potent and selective degrader of LIMK2 (LIM domain kinase 2).

##### Synthesis of dabrafenib-based PROTACs

**Figure 24.**
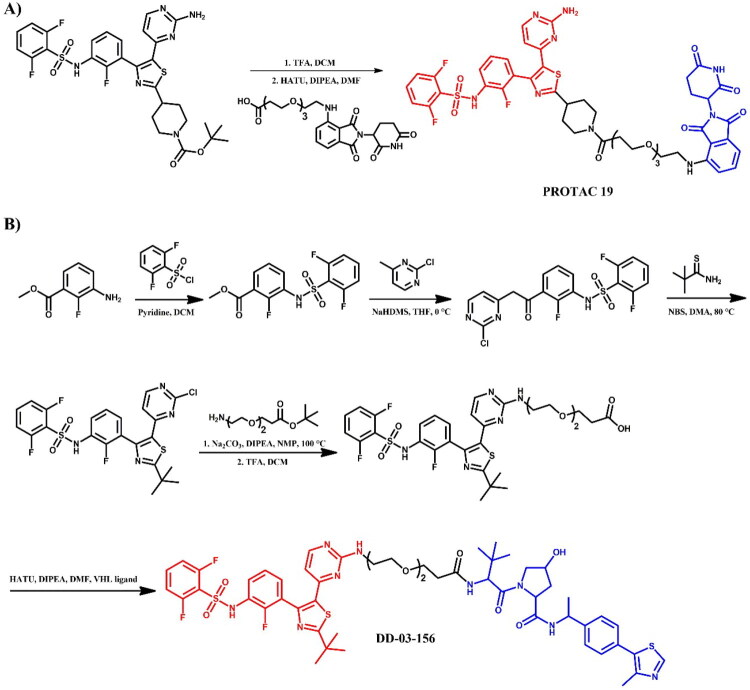
(a, b) Routes of synthesis of dabrafenib-based PROTACs (the figure was drawn by the authors using Chemdraw software).

Two dabrafenib-based PROTACs, **PROTAC 19** (Figure 24a) and **DD-03–156** (Figure 24b), were prepared with different starting materials due to the distinct linker attachments to the dabrafenib molecule. In **PROTAC 19**, the modification of the dabrafenib structure involved replacing the tert-butyl group attached to the thiazole ring with an *N*-protected piperidine. This modification allowed for linkage to the CRBN ligand via a modified PEG3-carboxylic acid in a coupling reaction. In contrast, the synthesis of **DD-03–156** employed a more complex approach to obtain the dabrafenib derivative, focusing on modifications that enabled subsequent linker attachment to the pyrimidine moiety. This sophisticated route began with a sulphonamide formation, followed by nucleophilic substitution to form a ketone, and finally, a cyclisation reaction to assemble the thiazole ring. The resulting dabrafenib derivative was then linked with linker via S_N_Ar and coupled to the VHL ligand.

#### Vemurafenib-based PROTACs

##### Rational design and development of vemurafenib-based PROTACs

**Figure 25.**
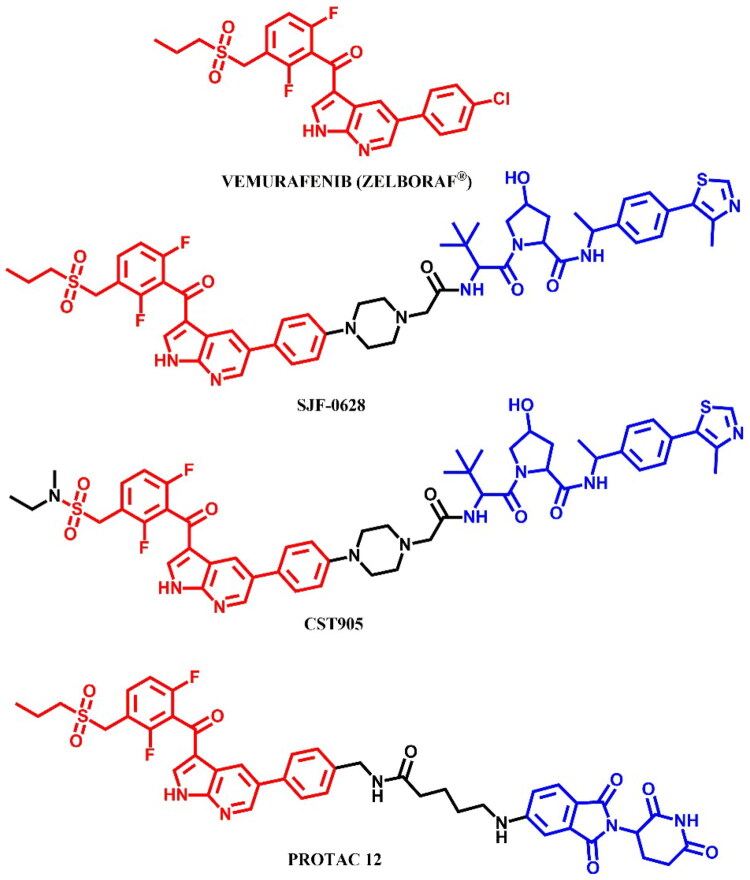
Structure of vemurafenib and vemurafenib-based PROTACs (the figure was drawn by the authors using Chemdraw software).

Miller et al.^46^ designed a series of VHL ligand-based PROTACs targeting the mutated BRAF^V600E^ kinase using vemurafenib-derived ligands. The design of these molecules was based on visual analysis of the crystal structures of BRAF^V600E^ in complex with vemurafenib (PDB ID: 4XV1) and revealed a solvent-exposed phenyl ring on the azaindole as a potential site for suitable linker attachment. PROTAC **CST905** (Figure 25) was developed as a potent BRAF^V600E^ degrader (IC_50_ = 61 nM, DC_50_ = 1 μM; for **SJF-0628**: IC_50_ = 33 nM, DC_50_ = 1 μM), which did not induce paradoxical MAPK activation in RAS-mutant cell lines, which is common during BRAF inhibition in tumours with additional mutations in RAS or its upstream signalling receptors. These mechanism of action distinguished **CST905** from other vemurafenib-based PROTACs (e.g. **SJF-0628**^47^) ensuring significantly more durable efficacy and safer profile.

Han et al.^48^ developed vemurafenib-based **PROTAC 12** (Figure 25), which selectively induced degradation of BRAF^V600E^, but not wild-type BRAF, despite its ability to bind to both proteins with similar affinities (K_d_ = 14.4 nM for BRAF^V600E^ and K_d_ = 9.5 nM for BRAF^WT^; for vemurafenib: K_d_ = 58.2 nM for BRAF^V600E^ and K_d_ = 50.7 nM for BRAF^WT^). This study showed evidence that PROTAC molecules differentially degrade closely related target proteins, suggesting that forming a ternary complex of POI and E3 ligase is necessary but insufficient to induce the degradation process. The most potent molecule was **PROTAC 12**, the low nanomolar degrader, which provoked *in vitro* antineoplastic activities in melanoma cells A375 (IC_50_ = 500 nM; for vemurafenib: IC_50_ = 116 nM) and colon cancer cells HT-29 (IC_50_ = 124 nM; for vemurafenib: IC_50_ = 164 nM). Its structure consist of short alkyl linker attached by amide bond to dabrafenib, in the same position as **CST905**^46^, and CRBN ligand. The authors also indicated a relationship between linker length and potency for the degradation of BRAF^V600E^ pointing out that a shorter linker was associated with higher potency, but only for vemurafenib as the warhead, whereas degraders based on the RAF kinase inhibitor BI882370 required a longer linker.

Alabi et al.^47^ used vemurafenib-based PROTACs to achieve low nanomolar degradation of all classes of BRAF mutants but spare degradation of wild-type RAF family members. The most potent PROTAC **SJF-0628** (Figure 25) caused dose-dependent decrease in the level of all three classes and outperformed vemurafenib in inhibiting cancer cells growth (SK-MEL-28/Class 1: EC_50_ = 37 nM; for vemurafenib: EC_50_ = 215 nM; SK-MEL-246/Class 2: EC_50_ = 45 nM; for vemurafenib: EC_50_ > 1 μM; H1666/Class 3: EC_50_ = 218 nM), and showed *in vivo* efficacy in second class BRAF xenograft model. The degradation-incompetent control, SJF-0661, with inverted stereocentre of the critical hydroxyproline group in the VHL ligand, showed no reduction in BRAF protein levels. Investigation of the selectivity of the SJF-0628 revealed that BRAF^WT^ was unable to form a PROTAC-induced ternary complex *in cellulo*. Another finding was that measuring ternary complexes in cell lysates or cells was more predictive of degradation than *in vitro* studies with purified proteins, which was also observed by Posternak et al.^44^.

##### Synthesis of vemurafenib-based PROTACs

**Figure 26.**
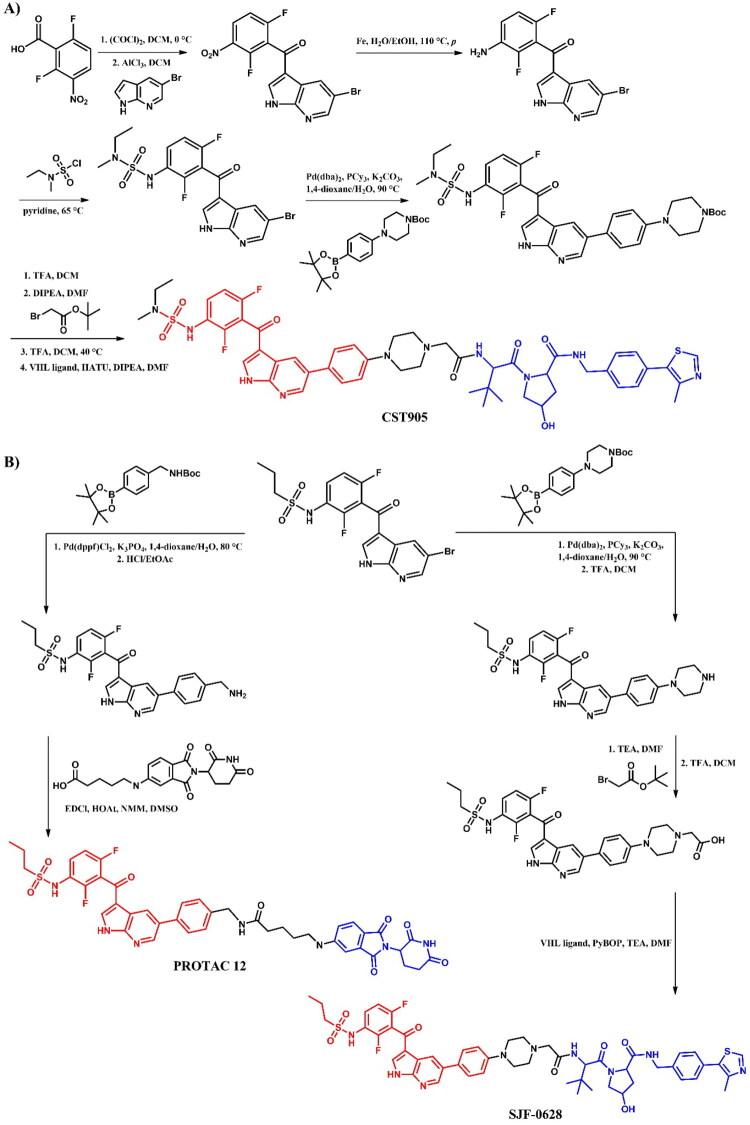
(a, b) Routes of synthesis of vemurafenib-based PROTACs (the figure was drawn by the authors using Chemdraw software).

Due to structural differences in the sulphonamide part of vemurafenib, the synthesis of **CST905** (Figure 26a) involved a different starting material compared to **PROTAC 12** or **SJF-0628** (Figure 26b), which shared the same precursor. The synthesis of **CST905** began from a 2,6-difluoro-3-nitrobenzoic acid. Its preparation involved a multi-step process including a selective acylation, nitro group reduction, and subsequent sulphonamide formation, followed by a Suzuki-Miyaura reaction and final coupling to the VHL ligand. In contrast, the synthesis of **PROTAC 12** and **SJF-0628** started from the same vemurafenib precursor. For both, the first step was the Suzuki-Miyaura reaction followed by deprotection of the amine group. For **PROTAC 12**, the final step was the coupling reaction with 5-thalidomide-6C carboxylic acid. In the case of **SJF-0628**, after the Suzuki-Miyaura reaction, the linker was extended by substitution with carboxylic acid, followed by deprotection and coupling with VHL ligand. Interestingly, each of these vemurafenib-based PROTACs employed different conditions in the final coupling reactions. **CST905** used HATU, an uronium-based coupling reagent; **PROTAC 12** utilised EDCl, a carbodiimide-based reagent; and **SJF-0628** employed PyBOP, a phosphonium-based reagent.

#### Encorafenib-based PROTAC

##### Rational design and development of encorafenib-based PROTAC

**Figure 27.**
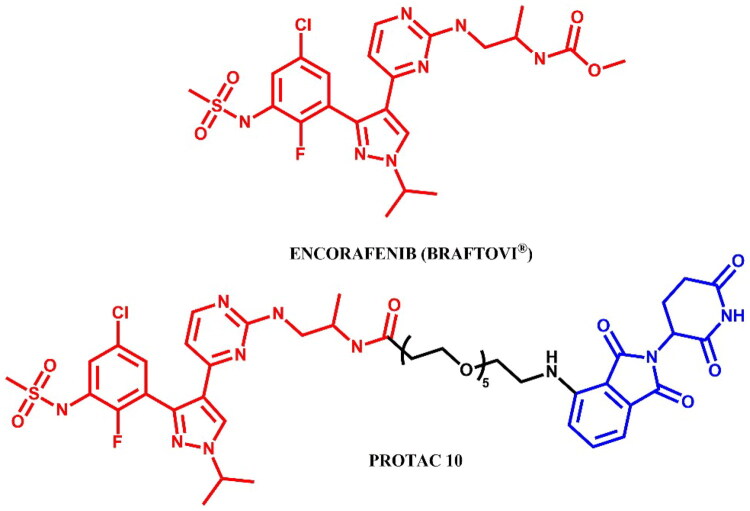
Structure of encorafenib and encorafenib-based PROTAC (the figure was drawn by the authors using Chemdraw software).

Marini et al.^49^ designed a series of compounds in which encorafenib was conjugated to pomalidomide via linkers of varying lengths and flexibilities. The resulting compounds were able to inhibit the activity of BRAF^V600E^ in the nanomolar range, even better than encorafenib, and consequently reduce cell proliferation in the A375 and Colo205 tumour cell lines due to decreasing the phosphorylation of MEK and ERK. The most potent molecule was **PROTAC 10** (Figure 27) (in A375 cell line: IC_50_ = 194 nM; in Colo205 cell line: IC_50_ = 31 nM), but it was unable to induce target protein degradation. The authors hypothesised, according to *in silico* research, that the orientation of the linker attached to the pomalidomide structure was different compared to other documented active degraders of BRAF^V600E^, which had an unfavourable effect on the protein-protein interface and the formation of the ternary complex. The aforementioned PROTACs were only able to inhibit the activity of BRAF^V600E^ kinase but without any degradation activity.

The synthesis of encorafenib-based PROTAC was described in only two steps – a hydrolysis and a coupling reaction - making it analogous to other methods already detailed in this paper (e.g. Figures 15, 17, and 22). Consequently, we have chosen to omit this synthesis route from the graphical representation.

### ATC L01ED – Anaplastic lymphoma kinase (ALK) inhibitors

**Figure 28.**
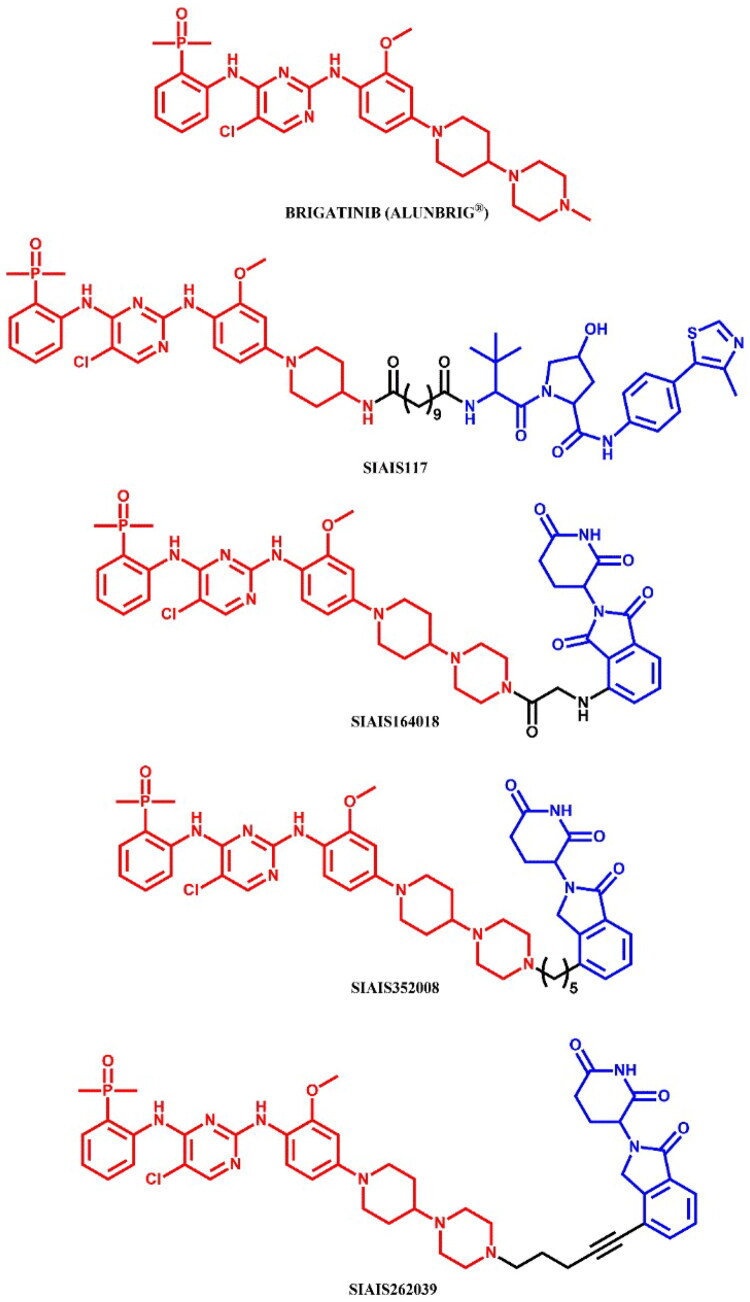
Structure of brigatinib and brigatinib-based PROTACs (the figure was drawn by the authors using Chemdraw software).

Anaplastic lymphoma kinase (ALK) is a receptor tyrosine kinase involved in the development and function of the nervous system, particularly during embryogenesis^96^. ALK is encoded by the *ALK* gene and consists of an extracellular ligand-binding domain, a transmembrane segment, and an intracellular kinase domain. In healthy adults, ALK expression is low and has a minimal effect on cellular activities. Upon ligand binding, ALK triggers dimerisation and autophosphorylation, activating downstream signalling pathways that control cell growth, differentiation, and survival. In cancer, alterations such as mutations, amplifications, and chromosomal rearrangements of the *ALK* gene can lead to its constitutive activation, resulting in oncogenic signalling. ALK rearrangements are frequently associated with anaplastic large cell lymphoma (ALCL) and non-small cell lung cancer (NSCLC)^97^. The first FDA-approved ALK inhibitor was crizotinib, a first-generation drug, followed by second-generation inhibitors like alectinib, ceritinib, and brigatinib, which offer enhanced specificity and effectiveness associated with earlier therapies^97^. All of these drugs have been used in the PROTAC technology (Figures 28, 30, and 32).

#### Brigatinib-based PROTACs

##### Rational design and development of brigatinib-based PROTACs

Sun et al.^50^ developed brigatinib-based PROTACs targeting ALK, identifying **SIAIS117** (Figure 28) as the lead degrader. **SIAIS117** not only inhibited growth in SR (IC_50_ = 1.7 nM; for brigatinib: IC_50_ = 2.7 nM) and H2228 (IC_50_ = 46 nM; for brigatinib: IC_50_ = 58.2 nM) cell lines, but also significantly degraded ALK protein (DC_50_ = 7.0 nM), demonstrating superior growth inhibition compared to brigatinib in 293 T cells expressing the ALK^G1202R^ mutant (IC_50_ = 165.7 nM; for brigatinib: IC_50_ = 535.7 nM; for crizotinib: IC_50_ = 2109 nM). The authors utilised the co-crystal structure of ALK and brigatinib (PDB ID: 6MX8) to determine the solvent-exposed piperidine moiety as an attachment point for linkers. Altering alkyl linker length affected growth inhibition - shorter linkers (C2-C5) weakened efficacy, while longer linkers (C6-C9) restored it, with optimal potency observed for the C9 linker used in **SIAIS117**. Proteomic analysis revealed significant downregulation of kinases UCK2 and GAK upon **SIAIS117** treatment, but showed no effect on other kinases, such as EGFR mutations, IGF-1R, FLT3, ROS1, or Aurora A. Interestingly, **SIAIS117** was also tested on several SCLC cell lines, which do not depend on ALK expression for proliferation, and showed notable cell-killing effects in these SCLC lines, indicating potential broader applicability.

Ren et al.^51^ designed and developed **SIAIS164018** (Figure 28), a PROTAC capable of degrading ALK^G1202R^ mutant and EGFR^L858R/T790M^ mutations, both key ALK fusion proteins in NSCLC. **SIAIS164018** potently inhibited cell migration and invasion in Calu-1 and MDA-MB-231 cell lines, and also degraded oncoproteins associated with metastasis, such as FAK, PYK2, and PTK6 (DC_50_ < 1 nM). This PROTAC showed strong oral bioavailability and good toleration *in vivo*. **SIAIS164018** exhibited potent inhibition of SR cell proliferation (IC_50_ = 2 nM; for brigatinib: IC_50_ = 3.3 nM), and induced the degradation of NPM-ALK and EML4-ALK proteins. **SIAIS164018** also showed greater cell proliferation inhibition in ALK^G1202R^-overexpressing 293 T and EGFR-expressing H1975 cell lines than brigatinib and induced a stronger G1 cell cycle arrest. Proteomic analysis revealed that **SIAIS164018** downregulated the key proteins - FAK, PYK2, FER, RSK1, and GAK - in both ALK-positive SR and ALK-negative Calu-1 cell lines. The degraded targets FAK, PYK2, and ALK exhibited different degradation rates and recovery patterns, indicating target-specific dynamics.

Zhang et al.^52^ developed two potent brigatinib-based PROTACs, **SIAIS352008** and **SIAIS262039** (Figure 28), targeting FER, a nonreceptor tyrosine kinase critical for cell proliferation, motility, intercellular adhesion, and signal transduction to the cytoskeleton. These PROTACs demonstrated superior efficacy over brigatinib in suppressing ovarian cancer cell motility. Notably, both compounds effectively degraded multiple oncogenic FER fusion proteins identified in human tumours (for both: DC_50_ < 1 nM). **SIAIS352008** and **SIAIS262039** showed improved FER degradation efficiency compared to the previously described **SIAIS164018**^51^. Both compounds effectively degraded endogenous and exogenous FER across nine ovarian carcinoma cell lines in a concentration-dependent manner, without a hook effect. Proteomic analysis revealed that, aside from FER, these PROTACs also targeted and degraded AAK1 and GAK kinases. Although **SIAIS352008** exhibited limited impact on cancer cell proliferation and survival, it effectively suppressed ovarian cancer cell motility and invasiveness *in vivo*.

##### Synthesis of brigatinib-based PROTACs

**Figure 29.**
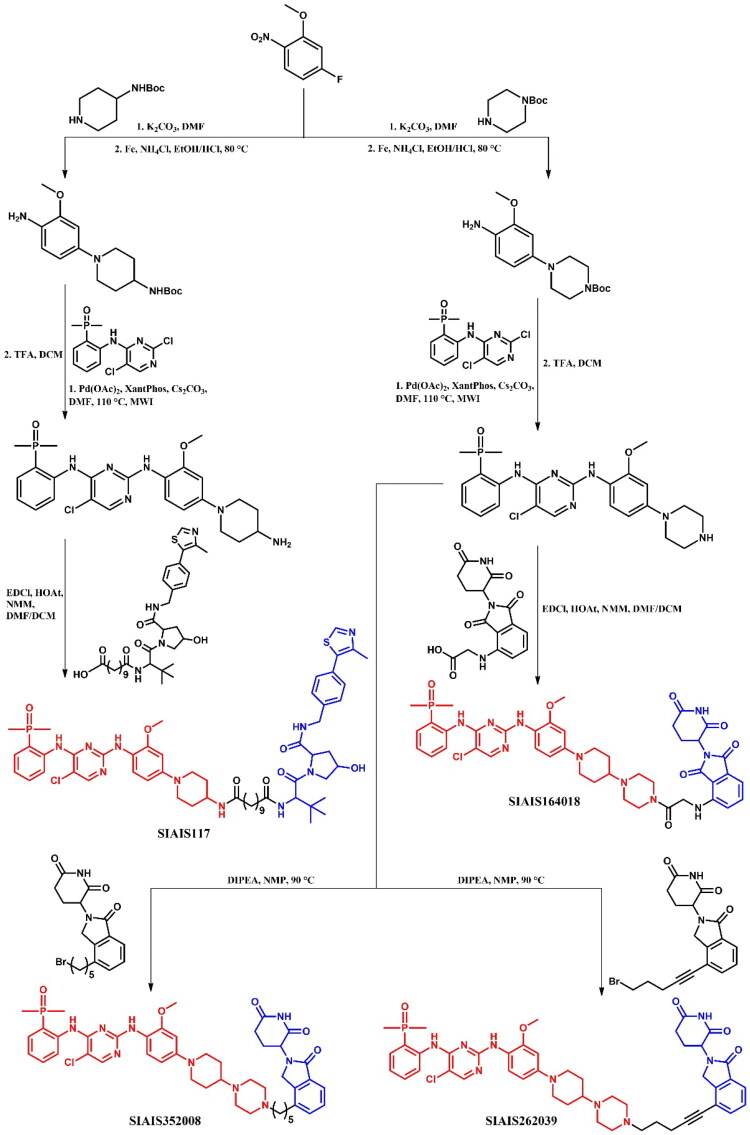
Routes of synthesis of brigatinib-based PROTACs (the figure was drawn by the authors using Chemdraw software).

The starting material was the same for all brigatinib-based PROTACs, but the first amine attached to this substrate varied depending on whether the piperazine moiety was kept or removed from brigatinib to attach the linker (Figure 29). For **SIAIS117** (a PROTAC where the piperazine was removed), the synthesis began with a S_N_Ar reaction between fluorinated nitroanisole and Boc-protected piperidine derivative. This was followed by the reduction of the nitro group to an amine, after which the material underwent a microwave-promoted Buchwald-Hartwig. The final step involved a coupling reaction with a carboxylic acid-substituted VHL ligand. For the other PROTACs, the synthetic route was almost identical, but instead of Boc-protected piperidin-4-amine, Boc-protected piperazine was used in the first step. For **SIAIS164018**, the final step involved coupling with a carboxylic acid-substituted pomalidomide instead of the VHL ligand. In contrast, for **SIAIS352008** and **SIAIS262039**, the final step involved substitution with the appropriate bromides of pomalidomide.

#### Alectinib-based PROTACs

##### Rational design and development of alectinib-based PROTACs

**Figure 30.**
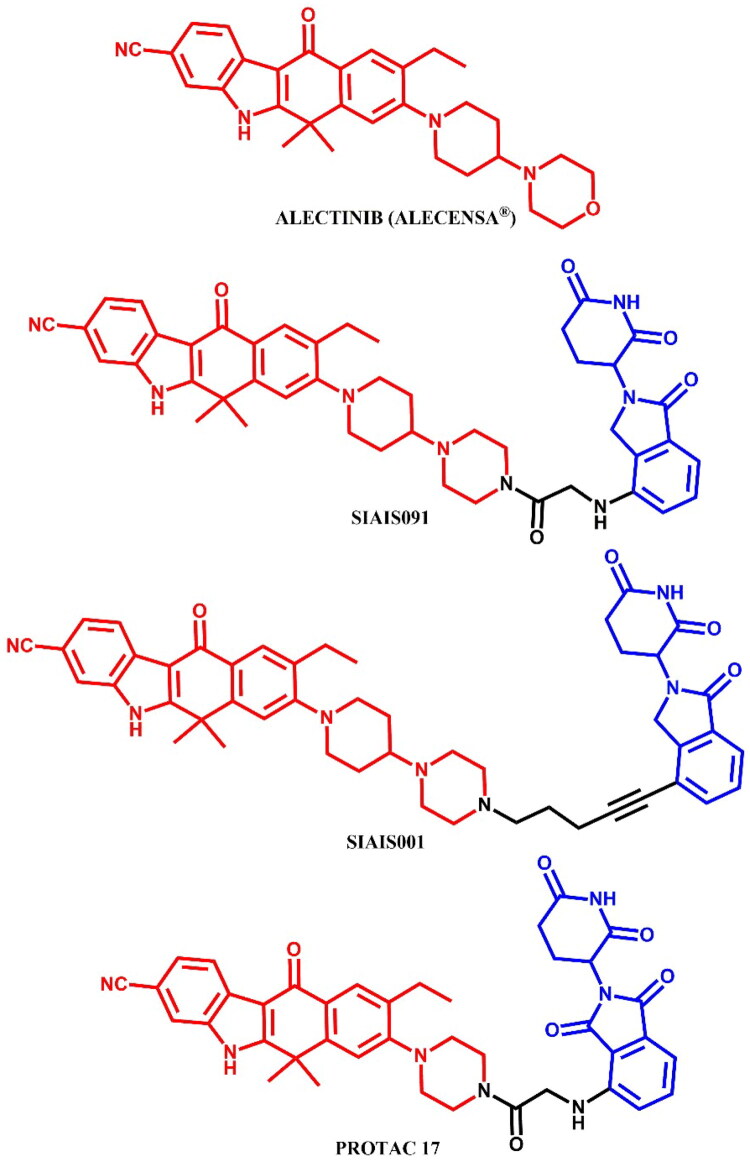
Structure of alectinib and alectinib-based PROTACs (the figure was drawn by the authors using Chemdraw software).

Ren et al.^53^ developed an orally bioavailable small-molecule degraders targeting ALK protein by combining an alectinib derivative with a CRBN ligand. **SIAIS001** and **SIAIS091** (Figure 30) emerged as the most potent compounds. In terms of antiproliferative effects on SR cells, **SIAIS091** was more than five times more potent than alectinib (IC_50_ = 0.5 nM; for alectinib: IC_50_ = 3.4 nM), while **SIAIS001** exhibited inhibition of downstream pathways. Both compounds were effectively bound to the ALK^G1202R^ (for **SIAIS001**: IC_50_ = 3.3 nM; for **SIAIS091**: IC_50_ = 4.7 nM), but only **SIAIS091** induced slight degradation at 200 nM. Both compounds promoted cell cycle arrest in G1/S phase, with **SIAIS001** showing better pharmacokinetic properties. For their design, the authors used the co-crystal structure of ALK and alectinib (PDB ID: 3AOX), targeting the solvent-exposed morpholine moiety as an attachment site for linkers. They investigated linker modifications, finding that a shorter carbon linker (specifically in **SIAIS001**) led to optimal antiproliferative effects. Through SAR studies, they observed that degraders with lenalidomide as E3 ligand exhibited better potency than those using pomalidomide.

Xie et al.^54^ synthesised alectinib-based PROTACs by linking two alectinib precursors with pomalidomide through varying linkers. Biological assays highlighted **PROTAC 17** (Figure 30) as the most promising compound, demonstrating strong ALK binding affinity (IC_50_ = 13.63 nM), efficient ALK fusion protein degradation (in H3122 cell line: DC_max_ = 83.6% (1 μM); in Karpas 299 cell line: DC_max_ = 85.0% (1 μM)), and antiproliferative effects in ALK-positive cell lines (in H3122 cell line: IC_50_ = 62.0; in Karpas 299 cell line: IC_50_ = 42.0). **PROTAC 17** had limited cytotoxicity in low ALK-expressing lines (A549 and HFL-1), underlining its selectivity. *In vivo*, **PROTAC 17** inhibited tumour growth by nearly 76% through intravenous administration (10 mg/kg/day) without body weight loss, outperforming alectinib in a Karpas 299 xenograft model. **PROTAC 17** also overcame ALK inhibitor-resistant mutations (L1152R, G1202R, and L1196M) in Ba/F3 cells, comparable to alectinib. Design modifications included the removal of the solvent-exposed morpholine group of alectinib and the replacement of the piperidine moiety with piperazine. **PROTAC 17** induced dose-dependent degradation of the ALK fusion protein, achieving complete degradation in H3122 and Karpas 299 cells at nanomolar DC_50_. This degradation correlated with increased inhibition of downstream signalling pathways and enhanced antiproliferative effects in ALK-positive cells, suggesting combined inhibition and degradation as a mechanism of action. Further proteomic analysis identified JUNB, SESN2, and SOCS3 as the only proteins significantly reduced by **PROTAC 17**, indicating high selectivity.

##### Synthesis of alectinib-based PROTACs

**Figure 31.**
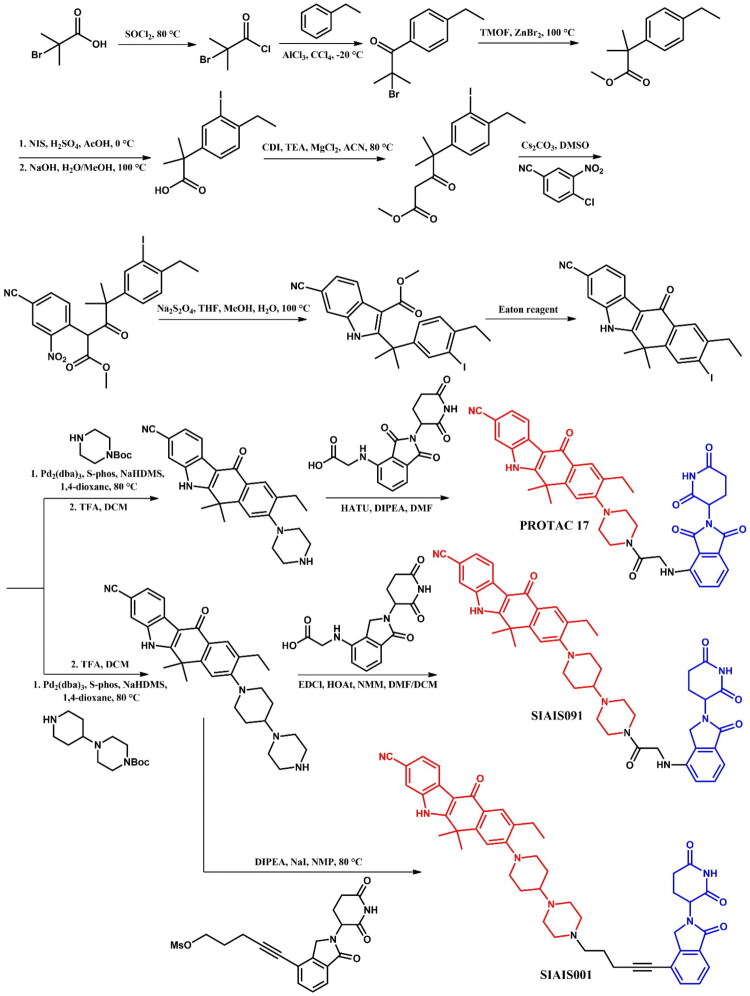
Routes of synthesis of alectinib-based PROTACs (the figure was drawn by the authors using Chemdraw software).

The synthesis of all alectinib-based PROTACs initiated from a common, multi-step route to construct the complex tetracyclic core (Figure 31). This lengthy early-stage preparation involved sequential reactions including a Friedel-Crafts acylation, a quasi-Favorskii rearrangement, regioselective iodination, followed by indole ring formation and final intramolecular cyclisation. From this point, the synthesis diverged depending on the specific PROTAC target. For **PROTAC 17**, Boc-protected piperazine was attached to the intermediate via a Buchwald-Hartwig coupling, followed by deprotection and coupling with carboxylic acid-substituted pomalidomide. For **SIAIS001** and **SIAIS091**, the intermediate was coupled with Boc-protected 1-(piperidin-4-yl)piperazine via the Hartwig-Buchwald reaction, then deprotected and attached to either a carboxylic acid-substituted lenalidomide or an alkyne derivative of lenalidomide.

#### Crizotinib-based PROTAC

##### Rational design and development of crizotinib-based PROTAC

**Figure 32.**
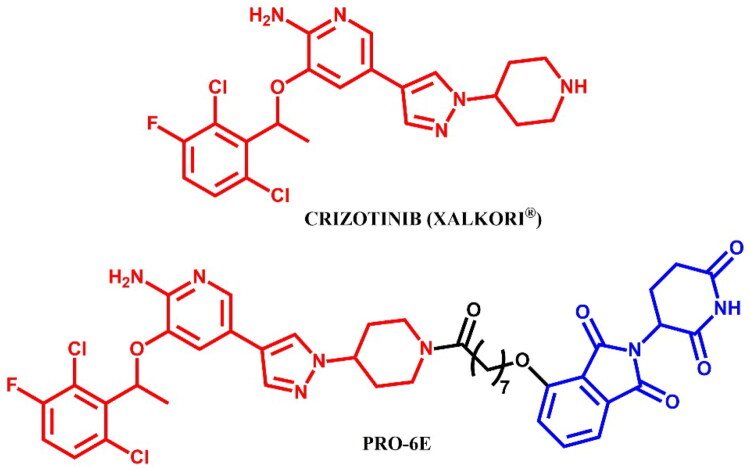
Structure of crizotinib and crizotinib-based PROTAC (the figure was drawn by the authors using Chemdraw software).

Chen et al.^55^ developed a MET-targeted crizotinib-based PROTAC, **PRO-6E** (Figure 32), which demonstrated high efficacy *in vitro* and *in vivo*, significantly inhibiting proliferation (IC_50_ = 0.347 μM) and motility in MET-positive gastric cancer (GC) cells and showing strong antitumor effects in the MKN-45 xenograft model. In designing **PRO-6E**, the MET/crizotinib complex X-ray crystal structure (PDB ID: 2WGJ) identified the nitrogen atom of piperidine moiety as the optimal linkage site. CRBN ligands were chosen over VHL-based ligands due to their stronger antiproliferative activities and favourable bioactivity profile in MKN-45 cells. In degradation studies, **PRO-6E** achieved a MET degradation maximum (D_max_) of 81.9% in MKN-45 cells. Molecular docking with the MET kinase domain showed two critical hydrogen bonds (Pro 1158 and Met 1160) and an H-π interaction with Ile 1084, which increased the binding affinity of **PRO-6E** over crizotinib (K_d_ = 5.38 μM; for crizotinib: K_d_ = 10.5 μM). While showing strong MET degradation, **PRO-6E** only weakly affected non-target proteins (IKZF1, IKZF3, GSPT1), highlighting its specificity. Notably, CRBN knockdown with siRNA - which is an approach uses a small, exogenous piece of RNA that base-pairs with a complementary target mRNA, leading to degradation of the mRNA - significantly reduced **PRO-6E** effectiveness, confirming the role of CRBN in MET degradation. Furthermore, **PRO-6E** increased the epithelial marker E-cadherin while decreasing mesenchymal markers (N-cadherin, vimentin), indicating epithelial-mesenchymal transition (EMT) suppression, with cells shifting from a dispersed to a compact morphology. **PRO-6E** effectively downregulated key downstream signalling proteins, including c-Myc and phosphorylated ERK, reinforcing its potential to inhibit tumour proliferation and metastasis through targeted MET degradation.

The synthesis of crizotinib was described only in its final step – a coupling reaction – and is therefore analogous to other methods already described in detail in the paper (e.g. Figure 26). The starting material was crizotinib without any modification due to the presence of a free amine group in its structure. Consequently, this route of synthesis has been omitted from the graphical representation.

#### Ceritinib-based PROTACs

##### Rational design and development of ceritinib-based PROTACs

**Figure 33.**
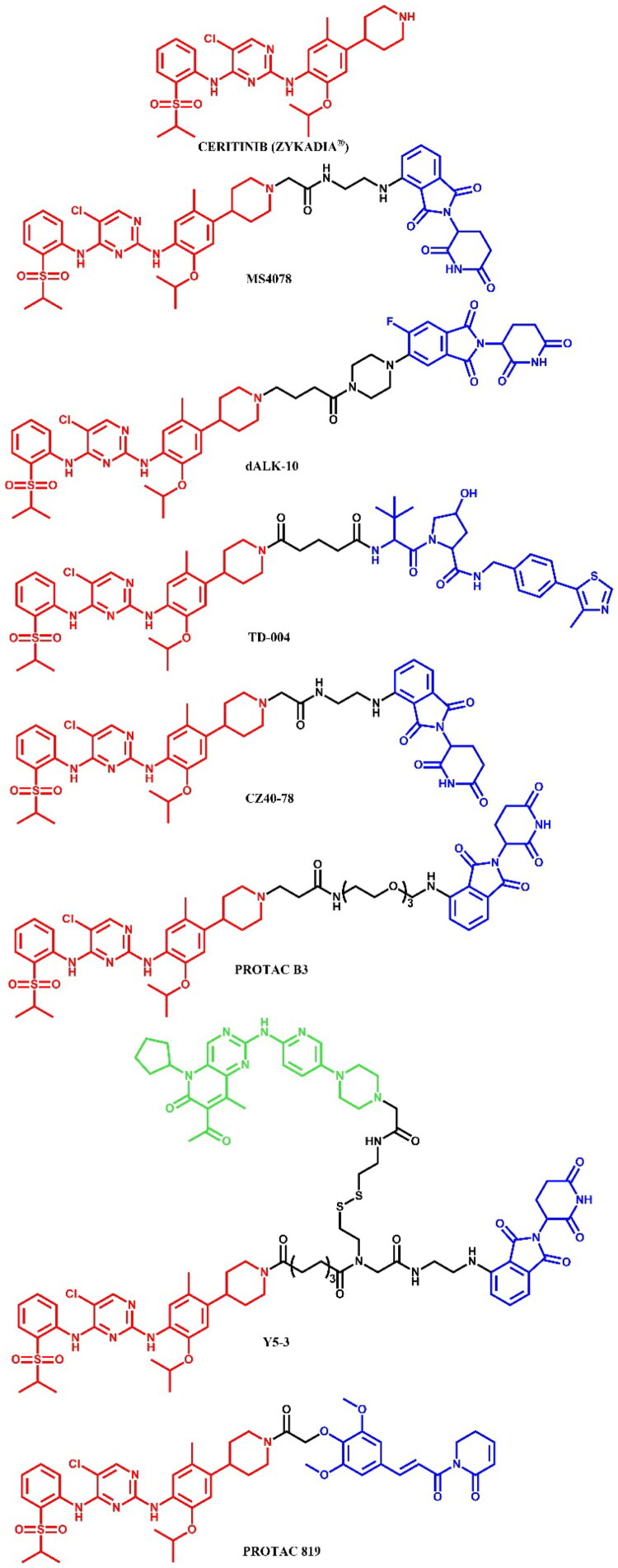
Structure of ceritinib and ceritinib-based PROTACs. Green colour indicates palbociclib (CDK4/6 inhibitor) (the figure was drawn by the authors using Chemdraw software).

Zhang et al.^56^ developed **MS4078** (Figure 33), a ceritinib-based PROTAC targeting ALK fusion proteins, which demonstrated significant concentration- and time-dependent ALK degradation in SU-DHL-1 lymphoma (DC_50_ = 11.0 nM) and NCI-H2228 lung cancer cells (DC_50_ = 59.0 nM). To design **MS4078**, the X-ray crystal structure of ALK with ceritinib (PDB ID: 4MKC) revealed the piperidinyl group as a solvent-exposed region. The ability to reduce ALK levels and inhibit ALK auto-phosphorylation and downstream STAT3 phosphorylation was confirmed in SU-DHL-1 and NCI-H2228 cells, expressing different ALK fusion proteins (NPM-ALK and EML4-ALK, respectively). Both long and short linkers were effective, with the long linker adopting a turn conformation to mimic the distance achieved by shorter linkers, forming productive ternary complexes for ALK degradation. While **MS4078** was only twice as potent as ceritinib inhibiting cell growth (IC_50_ = 15.0 nM), despite its lower binding affinity (K_d_ = 19.0 μM; for ceritinib: K_d_ = 1.3 μM), these results could be attributed to differences in the nature, localisation, and expression levels of ALK fusion proteins and/or cereblon dependency. However, Nguyen et al.^57^ later re-engineered **MS4078** to address off-target degradation, particularly affecting zinc-finger (ZF) proteins. Using a high-throughput platform to assess off-target effects, the team identified and synthesised 12 ALK-targeting PROTACs (dALK-1 to dALK-12) with variations such as an alkyne at the C4 and C5 positions, piperazine with different alkyl or acyl linkers, and diazaspiro[3.3]heptane groups. They also synthesised a C5 variant of **MS4078** [(5)-MS4078], that reduced off-target effects while retaining ALK degradation potency. In particular, the C5 alkyne exit vector in dALK-2 notably minimised off-target interactions with ZF proteins, including ZNF517 and ZNF653. The proteomic analysis confirmed that, while **MS4078** caused off-target degradation of SALL4 (associated with teratogenicity), **dALK-10** (Figure 33) did not, demonstrating the improvements achieved with the re-engineered compounds.

Kang et al.^58^ developed a series of ALK-targeting PROTACs by linking ceritinib with a ligand for VHL E3 ligase. Among these, **TD-004** (Figure 33) effectively induced ALK degradation and inhibited the growth of ALK fusion-positive SU-DHL-1 (IC_50_ = 58.0 nM) and H3122 cell lines (IC_50_ = 180.0 nM). In a xenograft model with H3122 cells, **TD-004** significantly reduced tumour growth. To assess the binding affinity of **TD-004** to the VHL E3 ubiquitin ligase, an AlphaScreen assay was conducted using the hydroxyproline residue of the HIF-1α peptide, known to interact with VHL. **TD-004** achieved over 90% degradation of the NPM-ALK protein in SU-DHL-1 cells at a concentration of 1 μM. Additionally, **TD-004** induced dose-dependent degradation of the EML4-ALK fusion protein in NCI-H3122, an ALK-positive NSCLC cell line.

The patent WO 2019/113071^59^ presented ceritinib-based PROTACs that effectively degraded or disrupted ALK, with the most potent compound, **CZ40-78** (Figure 33), achieving 97.9% inhibition of cell growth at 100 nM and over 80% ALK protein degradation in SU-DHL-1 cell lines at 30 nM. The degradation and inhibition effects of **CZ40-78** were both time- and concentration-dependent. Additionally, **CZ40-78** successfully blocked signal transduction pathways by inhibiting phosphorylation of ALK (p-ALK) and STAT3 (p-STAT3).

Yan et al.^60^ designed and synthesised a series of PROTACs targeting ALK by linking ceritinib to CRBN ligands with various linkers. Among these, **PROTAC B3** (Figure 33) demonstrated significant efficacy in degrading ALK in the H3122 cell line (>80% of degradation at 100 nM; IC_50_ = 1.6 nM; for ceritinib: IC_50_ = 0.81 nM), showing enhanced growth inhibition of H3122, H2228, and H1299 cells compared to ceritinib (for H3122: IC_50_ = 0.3 nM; for ceritinib: IC_50_ = 1.1 nM; for H2228: IC_50_ = 0.9 nM; for ceritinib: IC_50_ = 1.3 nM; for H1299: IC_50_ = 2.84 nM; for ceritinib: IC_50_ = 2.92 nM), with *in vivo* anticancer activity. Toxicity testing on the normal human liver cell line LO2 using an MTT assay revealed that **PROTAC B3** had reduced inhibitory effects compared to ceritinib, indicating that ALK degraders do not increase toxicity in normal cells. Bioassays confirmed that cells with ALK fusion proteins were more sensitive to ALK inhibitors and degraders than other cell types. **PROTAC B3** induced ALK degradation and downregulated of p-ALK and p-STAT3 in a concentration-dependent manner, with maximum degradation of EML4-ALK at 50 nM.

Wang et al.^61^ developed a novel glutathione (GSH)-responsive “Y-PROTAC”, specifically **Y5-3** (Figure 33), which effectively combines ALK degradation (DC_50_ = 2.83 μM) and CDK4/6 inhibition in a single multifunctional molecule. **Y5-3** features a disulphide (-S-S-) bond extended from the ALK-targeting PROTAC linker, conjugated to palbociclib (a CDK4/6 inhibitor), and cleaved by GSH to selectively release its functional moieties in tumour cells. Mechanistic studies demonstrated that **Y5-3** exhibited potent antiproliferative effects in H3122 cells (IC_50_ = 90.0 nM), achieving dual degradation of ALK and CDK4 under high GSH conditions. The selective GSH-sensitive design, enabled by cystine in the linker was critical in ensuring that **Y5-3** primarily targets cancer cells, sparing normal tissues with lower GSH levels. In several NSCLC cell lines, including H2228, H1299, and H3122, **Y5-3** showed superior antiproliferative activity compared to palbociclib (for H2228: IC_50_ = 1.76 μM; for ceritinib: IC_50_ = 0.59 μM; for palbociclib: IC_50_ = 1.42 μM; for H1299: IC_50_ = 4.73 μM; for ceritinib: IC_50_ = 1.62 μM; for palbociclib: IC_50_ = 0.27 μM; for H3122: IC_50_ = 0.09 μM; for ceritinib: IC_50_ = 0.06 μM; for palbociclib: IC_50_ = 0.26 μM). **Y5-3** selectively degraded CDK4 (DC_50_ = 3.69 μM), sparing CDK6, with mechanistic studies suggesting that disulphide linkage and positioning may influence selectivity. Control compounds Me-Y5-3 (with a methyl modification at the pomalidomide binding site) and Y5-3-C2 (with a -C-C- linkage in place of -S-S-) confirmed CRBN-dependent degradation, as Me-Y5-3 and Y5-3-C2 lacked dual degradability. **Y5-3**, however, effectively downregulated ALK and CDK4, blocking cell cycle progression at the G0/G1 phase, reducing colony formation, and significantly diminishing tumour volume and weight *in vivo*.

Pei et al.^62^ introduced a novel approach to targeted protein degradation by identifying piperlongumine (PL) as a covalent E3 ligase recruiter. The authors designed **PROTAC 819** (Figure 33), a conjugate of PL and ceritinib, specifically for degrading the EML4-ALK fusion protein in NCI-H2228 NSCLC cells. The degradation of EML4-ALK by **PROTAC 819** was concentration-dependent and could be blocked by proteasome and ubiquitin-activating enzyme inhibitors (MG132, MLN4924) as well as by dimethyl fumarate (DMF), indicating its reliance on the UPS. These results also suggested that **PROTAC 819** may recruit the KEAP1 E3 ligase complex to mediate targeted degradation in a proteasome-dependent manner. The study not only highlighted PL potential as a covalent E3 ligase ligand for targeted protein degradation but also pointed to broader applications of PL in covalent recruitment for the development of other degraders targeting specific oncoproteins.

##### Synthesis of ceritinib-based PROTACs

**Figure 34.**
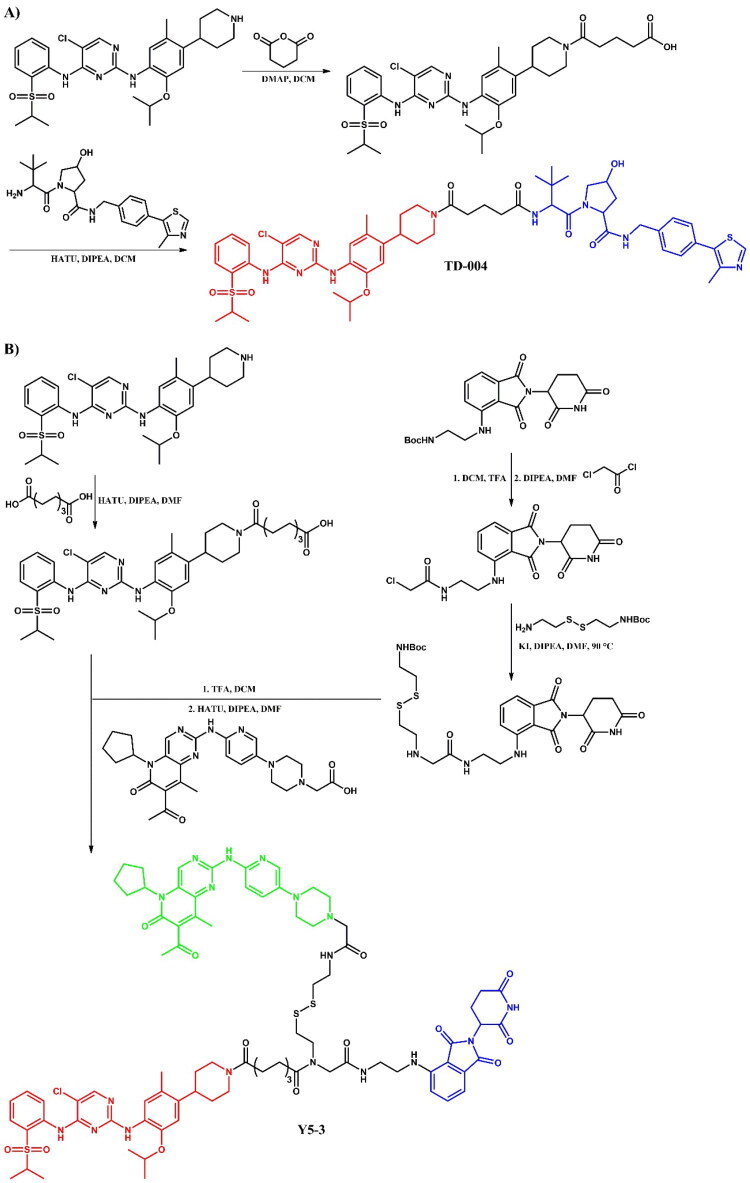
(a, b) Routes of synthesis of ceritinib-based PROTACs (the figure was drawn by the authors using Chemdraw software).

The synthesis of all ceritinib-based PROTACs, except **Y5-3**, followed by a similar scheme due to the presence of a reactive amino group in the ceritinib, that binds easily to the linker and is readily available as a commercial starting material. Due to the similarity of the synthetic routes for these six PROTACs, only one representative example - **TD-004** - is shown in the graphical representation, apart from mentioned **Y5-3**. The synthesis scheme for **TD-004** involved the attachment of the linker to ceritinib, followed by coupling with the VHL ligand (Figure 34a). In contrast, the synthesis of **Y5-3** required a different approach involving the derivatisation of three ligands: carboxylic acid-substituted ceritinib, pomalidomide with a disulphide bond, and carboxylic acid-substituted palbociclib (Figure 34b). These ligands were sequentially conjugated into a single molecule, first by coupling the ceritinib and pomalidomide derivatives, followed by the addition of the palbociclib derivative.

### ATC L01EF – cyclin-dependent kinases (CDKs) inhibitors

**Figure 35.**
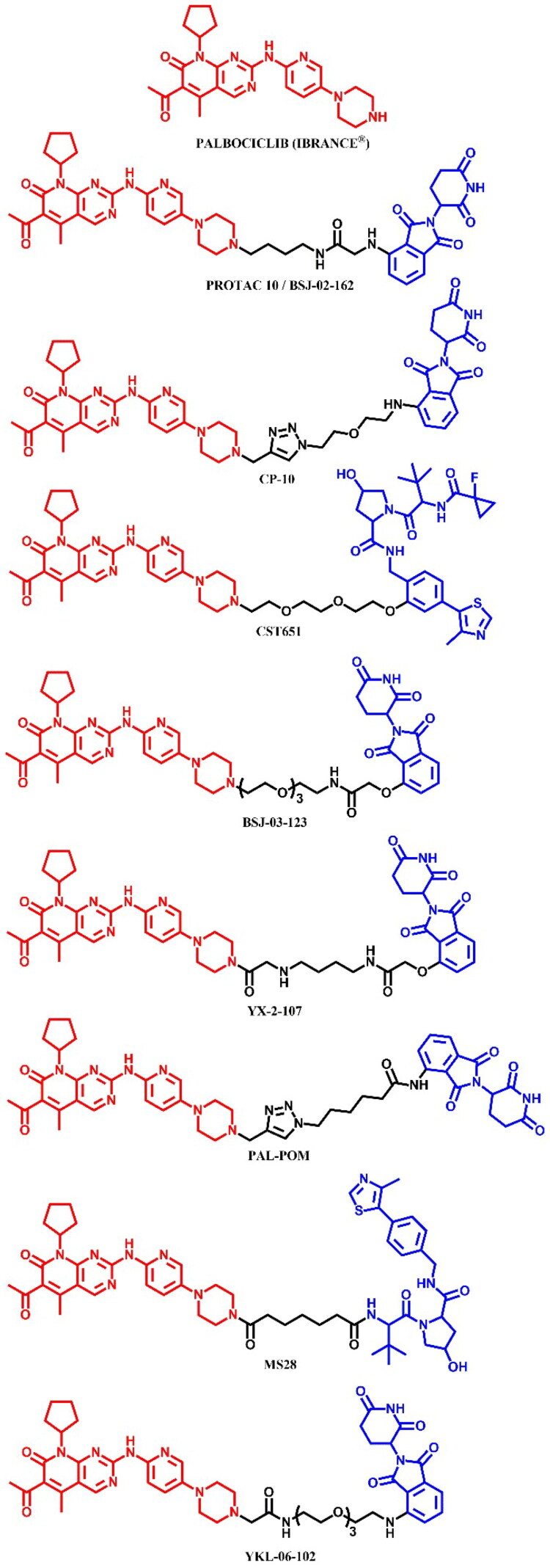
Structure of palbociclib and palbociclib-based PROTACs (the figure was drawn by the authors using Chemdraw software).

Cyclin-dependent kinases (CDKs) are a family of serine/threonine protein kinases that play a critical role in the regulation of the cell cycle, transcription, and other key cellular processes^98^. CDKs function in complex with regulatory proteins called cyclins, which activate the kinase activity of CDKs and ensure proper progression through various phases of the cell cycle, including G1, S, G2, and M phases. By phosphorylating target proteins, CDKs and cyclins together control essential events such as DNA replication, mitosis, and cell division. Dysregulation of CDK activity, often due to overexpression, mutation, or loss of regulatory control, is commonly associated with several types of cancer. Increased CDK4/6 activity leads to uncontrolled progression through the G1 phase and promotes tumourigenesis. As a result, CDK inhibitors, such as palbociclib, ribociclib, and abemaciclib, have been developed as targeted cancer therapies to block CDK activity and restore cell cycle control in cancer cells^98^. These inhibitors have also been extensively used in the PROTAC technology (Figures 35, 39, and 40).

#### Palbociclib-based PROTACs

##### Rational design and development of palbociclib-based PROTACs

In the 2019, Zhao and Burgess^63^, in the first CDK4/6 PROTACs study, designed PROTACs based on palbociclib and ribociclib molecules which efficiently degrade CDK4/6 proteins and inhibit retinoblastoma (RB) phosphorylation, leading to cell cycle arrest. Both **PAL-POM** (Figure 35) and **RIB-POM** (Figure 39) PROTACs were tested in MDA-MB-231 triple-negative breast cancer cells, where **PAL-POM** dose-dependently depleted CDK4 and CDK6, although **RIB-POM** was less efficient. **PAL-POM** was found to degrade CDK4 more efficiently than CDK6 (DC_50_ = 12.9 nM and 34.1 nM, respectively), and showed better degradation efficiency than **RIB-POM** (DC_50_ = 97.0 nM and >300 nM, respectively). The use of the neddylation inhibitor MLN4924 further confirmed the involvement of cullin-RING ligases in PROTAC-mediated degradation. Moreover, after washout of the PROTACs, CDK4/6 levels returned to their original state, indicating that the degradation process was reversible.

Jiang et al.^64^ described imide-based degraders that were based on palbociclib, ribociclib, or abemaciclib that degraded CDK4/6 or selectively degraded either CDK4 or CDK6. These PROTACs also targeted Ikaros (IKZF1) and Aiolos (IKZF3) and showed enhanced antiproliferative effects in mantle cell lymphoma (MCL) cell lines when both CDK4/6 and IKZF1/3 were degraded simultaneously. Structural studies revealed that the piperazine ring of palbociclib, ribociclib, and abemaciclib was solvent-exposed, making it suitable for attachment of linkers without affecting affinity for CDK4/6. PROTACs such as **BSJ-02–162** (palbociclib-based) (Figure 35), BSJ-01–152 (ribociclib-based), and **BSJ-01–184** (abemaciclib-based) (Figure 40) successfully degraded CDK4/6 in immunoblotting assays. Additionally, **BSJ-01–187** (Figure 39), a ribociclib-based degrader with a 4-carbon alkyl linker, selectively degraded CDK4, while the extended PEG-3 linker of palbociclib-derived **YKL-06–102** (Figure 35) selectively degraded CDK6. The degraders also targeted IKZF1/3, and **BSJ-02–162** caused G1 cell cycle arrest in wild-type Jurkat cells, with no effect in CRISPR/Cas9-modified Jurkat cells lacking cereblon. Treatment of MCL Granta-519 cells with **BSJ-02–162** resulted in the loss of CDK4/6 and IKZF1/3 proteins and showed increased antiproliferative effects compared to palbociclib or lenalidomide alone, suggesting that co-targeting CDK4/6 and IKZF1/3 may be an effective strategy for treating MCL.

Anderson et al.^65^ developed palbociclib-based PROTACs incorporating E3 ligase binders for CRBN, VHL, and IAP. All compounds demonstrated selective degradation of CDK6 over CDK4 in Jurkat cells, regardless of linker composition. Among them, the cereblon-recruiting **PROTAC 10** (**BSJ-02–162**) (Figure 35) was the most potent, with pDC_50_ values of 9.1 for CDK6 and 8.0 for CDK4. The observed CDK6 selectivity was consistent across the series. Proteasome dependency of degradation was confirmed using the inhibitor epoxomicin, which blocked CDK4/6 degradation.

Su et al.^66^ reported the design and synthesis of palbociclib-based PROTACs targeting CDK6 which induced the degradation of mutant CDK6 and effectively inhibited the proliferation of multiple myeloma (MM), leukaemia, and MCL cells. A computational study revealed that shorter linkers demonstrated higher degradation efficiency, suggesting more favourable spatial positions for CRBN recruitment towards CDK6. In addition, while different linker attachment points at the palbociclib end (amide, triazole, or methylene) produced PROTACs with similar degradation potential, the degradation potency was reduced eightfold when a flexible imino group on the linker at the pomalidomide side was replaced by a rigid alkyne. This finding suggested that CDK6 is insensitive to the rotational changes near its active kinase pocket, favouring flexible linkers for CRBN recruitment due to its narrow binding pocket. The most potent degrader, **CP-10** (Figure 35) induced nearly 72% degradation of CDK6 at 10 nM and 89% at 100 nM in human glioblastoma U251 cells, while CDK4 degradation was much weaker (DC_50_ = 2.1 nM and >100 nM, respectively). Carfilzomib, a proteasome inhibitor, completely blocked the PROTAC effect, confirming that the degradation was CRBN- and UPS-dependent. **CP-10** was tested in several cancer cell lines, including leukaemia (THP-1, HL-60), MCL (Mino, JeKo-1), and MM (MM.1S, RPMI8226), showing differential responses suggesting cell context specificity and differential dependence on CDK4/6. **CP-10** showed superior potency in the MM.1S cell line compared to pomalidomide, an approved treatment for MM. In addition, **CP-10** was tested in two independent palbociclib-resistant breast cancer cell lines with CDK6 copy amplification (MCF-7 CDK6N2) and FAT1 loss (MCF-7 FAT1 CR), where it successfully induced CDK6 degradation and inhibited proliferation. **CP-10** also degraded mutant forms of CDK6, such as D163G (which weakens palbociclib binding) and S178P (which mimics CDK4 activation and results in CDK6 hyperactivation), with the same efficiency as wild-type CDK6.

Steinbach et al.^67^ explored the CDK4/6 PROTACs by addressing different E3 ligases and connecting their respective ligands via various linkers to palbociclib. They extended the range of CDK6-specific PROTACs to include VHL and cIAP1 ligands. Among the VHL-based PROTACs, the authors replaced the valine-isoindolinone moiety of the VHL ligand with tert-leucine, acylated with either cyanocyclopropanecarbonyl or fluorocyclopropanecarbonyl groups. These modifications enhanced both potency and selectivity for CDK6 over CDK4. The most potent compound, **CST651** (Figure 35), exhibited over 95% CDK6 degradation at 100 nM and was stable under acidic conditions, in contrast to cereblon-based PROTACs, which degraded at physiological pH. **CST651** was effective in various cancer cell lines, including breast cancer, MM (MM.1S, LP-1, AMO-1), acute myeloid leukaemia (AML) (MOLM_12, HEL, KG-1, K562), and ALL (Nalm-6), and it was able to inhibit CDK’s kinase activity and other functions simultaneously. Conversely, IAP-based PROTACs degraded both CDK4/6 and IAP proteins, resulting in synergistic effects on cancer cell growth. These results showed that selectivity and activity can be modulated by tailored modifications in the target ligand and linker but also by choosing different E3-ligase binders.

Brand et al.^68^ developed a CDK6-selective PROTAC, **BSJ-03–123** (Figure 35), designed to exploit the dependence of AML cells on CDK6. **BSJ-03–123** achieved selective CDK6 degradation by distinguishing structural differences between CDK4 and CDK6 in the formation of ternary complexes. Remarkably, **BSJ-03–123** revealed signalling and transcriptional hubs associated with CDK6, such as BCL11A and NCOR2, highlighting PROTAC’s potential not only as a therapeutic agent but also as a tool to uncover previously unknown biochemical pathways and connections.

Dominici et al.^69^ developed CDK4/6-targeting PROTACs that preferentially degrade CDK6 over CDK4 in Philadelphia-positive (Ph^+^) ALL cells, significantly suppressing S phase cell proliferation and inhibiting CDK6-regulated phospho-retinoblastoma (RB) protein and FOXM1 expression. These effects were not observed in CD34^+^ normal haematopoietic progenitors, even though CDK6 was efficiently degraded. The palbociclib-based PROTAC **YX-2–107** (Figure 35), demonstrated potent CDK4/6 kinase inhibition *in vitro* (IC_50_ = 0.69 nM and 4.4 nM, respectively; for palbociclib: IC_50_ = 11.0 nM and 9.5 nM, respectively) and significantly reduced leukaemia burden *in vivo* in mice injected with Ph^+^ ALL cells, showing comparable or superior effects to palbociclib. The PROTAC **YX-2–107** induced rapid CDK6 degradation in BV173 cells, detectable within 1 h and significantly reduced CDK6 levels by 12 h. Proteomic analysis revealed that **YX-2–107** selectively degraded CDK6 without affecting other proteins, such as IKZF1 and IKZF3. In Ph^+^ BV173 and SUP-B15 cells, **YX-2–107** inhibited cell proliferation and FOXM1 expression; in the Jurkat T-ALL line, despite CDK6 degradation, S phase was not inhibited, suggesting that CDK4 compensates for CDK6 loss. Although **YX-2–107** showed less activity in normal CD34^+^ cells compared to Ph^+^ ALL cells, it did not affect the S phase or reduce phospho-RB levels in the same way.

Xiong et al.^70^ introduced an innovative approach known as a “bridged PROTAC”, designed to degrade the undruggable protein cyclin D1. Instead of binding directly to cyclin D1, this PROTAC engages CDK4/6, a binding partner of cyclin D1, to form a “bridge” that brings cyclin D1 into proximity with the VHL E3 ligase, triggering its degradation. As a result, the authors developed **MS28** (Figure 35), the first palbociclib-based PROTAC that effectively degrades cyclin D1 (DC_50_ = 0.95 μM, D_max_ = 90%). **MS28** demonstrated a faster and more effective degradation of cyclin D1 compared to CDK4/6, with notable effects on cancer cell proliferation, outperforming traditional CDK4/6 inhibitors. The mechanism was confirmed through various analyses. Isothermal titration calorimetry (ITC) showed that **MS28** does not directly bind cyclin D1 but rather degrades it through a quaternary complex formation with CDK4/6 and VHL. Proteomic profiling studies of 7500 proteins further confirmed that cyclin D1 and CDK4/6 were among the few proteins significantly reduced after **MS28** treatment, and the compound exhibited greater efficacy against NSCLC cells than standard CDK4/6 inhibitors (GI_50_ = 0.75 μM; for palbociclib: GI_50_ >10 μM; for BSJ-03–123: GI_50_ > 10 μM). This study demonstrated that PROTACs can target previously undruggable proteins by using a binding partner as a bridge, potentially expanding the range of druggable targets across the human proteome.

Bricelj et al.^71^ investigated the effect of linker attachment points on the stability and activity of CDK4/6-targeting PROTACs, given the inherent instability of certain cereblon ligands. The authors created a library of palbociclib-based PROTACs with thalidomide, pomalidomide, and lenalidomide linked by PEG3 chains. They found that **YKL-06–102** (Figure 35), a PROTAC with a pomalidomide moiety attached at position 4, was the most biologically active and stable. This configuration resulted in a slower recovery of CDK4/6 protein levels compared to other, less stable PROTACs, such as those with thalidomide carboxylic acid or alkyne derivatives at positions 4 and 5.

##### Synthesis of palbociclib-based PROTACs

**Figure 36.**
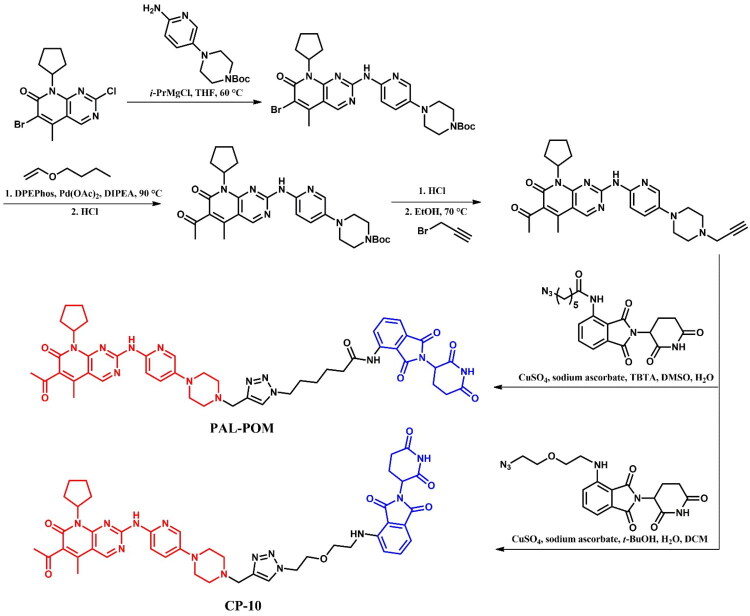
Routes of synthesis of palbociclib-based PROTACs. Part I (the figure was drawn by the authors using Chemdraw software).

**Figure 37.**
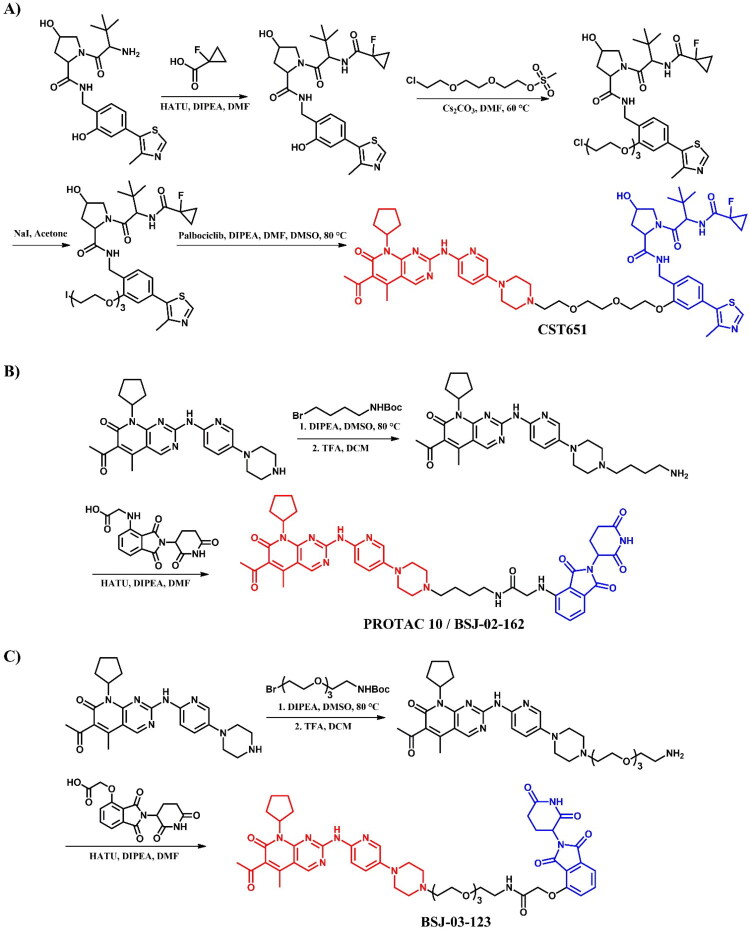
(a-c) Routes of synthesis of palbociclib-based PROTACs. Part II (the figure was drawn by the authors using Chemdraw software).

**Figure 38.**
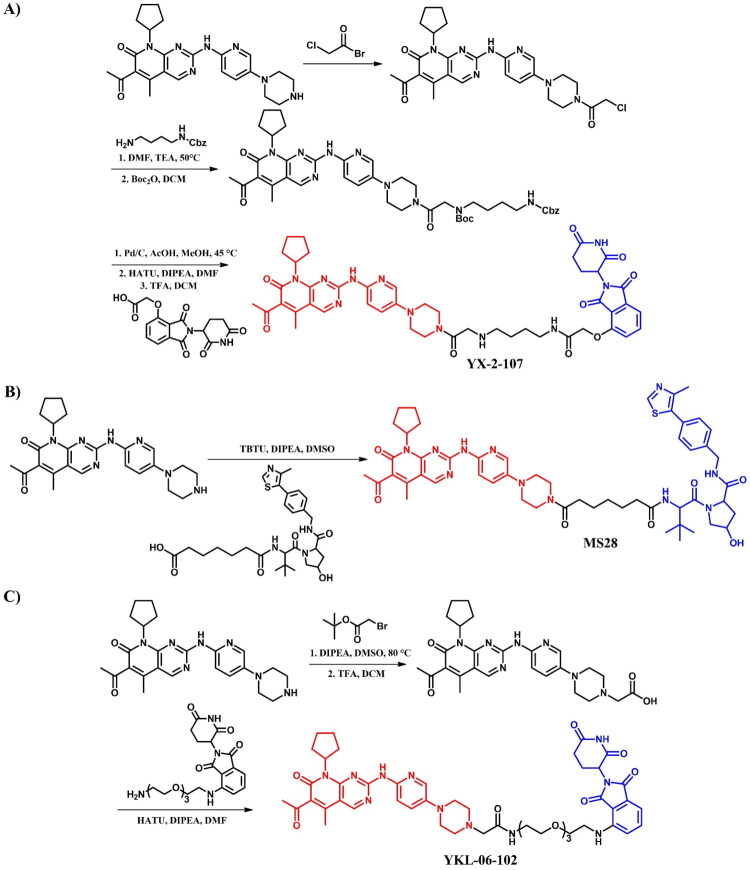
(a-c) Routes of synthesis of palbociclib-based PROTACs. Part III (the figure was drawn by the authors using Chemdraw software).

The synthesis of various palbociclib-based PROTACs presents diverse strategies for linker attachment and E3 ligase conjugation. For **PAL-POM** and **CP-10** (Figure 36), the synthesis involved introducing an alkyne moiety into the palbociclib core through a multi-step process that included an initial S_N_Ar, followed by Heck reaction and nucleophilic substitution. This alkyne was then utilised in a click reaction with an azide-modified pomalidomide derivative, forming a triazole ring and connecting it to the CRBN ligand. **CST651** (Figure 37a) involved the preparation of VHL ligand derivative enabling a substitution reaction to attach a palbociclib molecule. For **PROTAC 10/BSJ-02–162** (Figure 37b) and **BSJ-03–123** (Figure 37c), the strategy involved substituting palbociclib with an aminoalkyl bromide or PEG3 bromide linker, respectively, followed by coupling with a pomalidomide carboxylic acid derivative. For **YX-2–107** (Figure 38a), the piperazine moiety of palbociclib was functionalised through a sequence of substitution, protection/deprotection steps, and subsequent coupling to a pomalidomide derivative. Meanwhile, **MS28** (Figure 38b) utilised a straightforward coupling between palbociclib and conjugated VHL ligand with linker, emphasising a simpler synthetic strategy. Finally, **YKL-06–102** (Figure 38c) began with the substitution of palbociclib’s piperazine ring with a protected bromoacetic acid, followed by deprotection and coupling to an amine-PEG3 pomalidomide derivative.

#### Ribociclib-based PROTACs

##### Rational design and development of ribociclib-based PROTACs

Ribociclib-based PROTACs were explored alongside other CDK inhibitors, including palbociclib and abemaciclib. Therefore, this section will focus on additional information related to the development and biological activity of ribociclib-based PROTACs, omitting synthesis details as they closely mirror those for palbociclib-based compounds.

**Figure 39.**
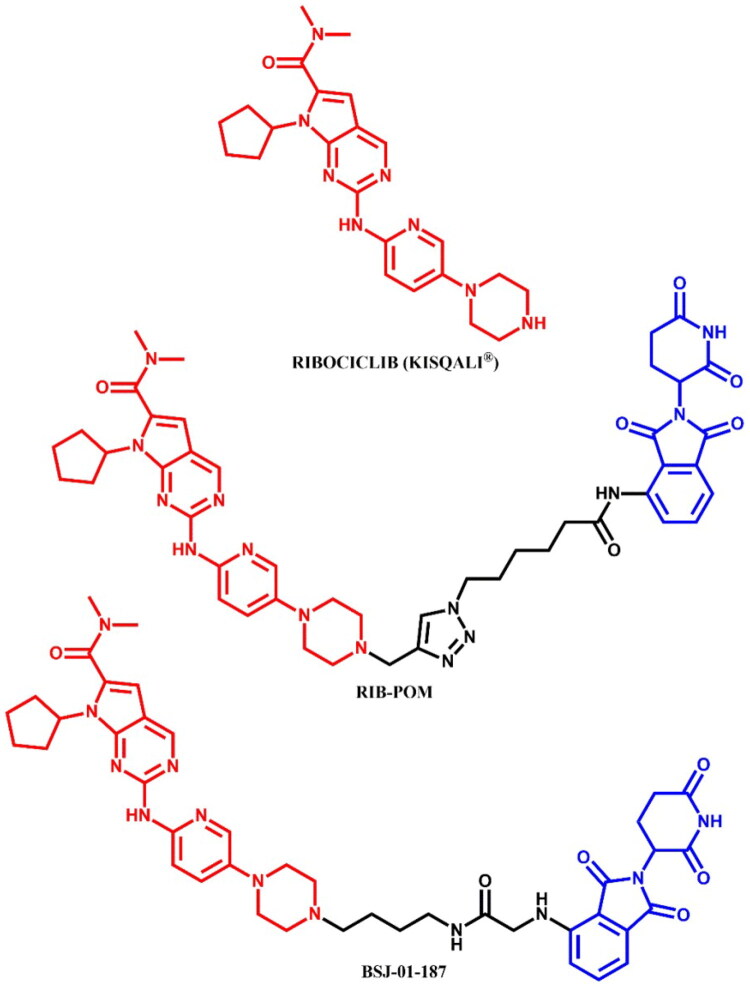
Structure of ribociclib and ribociclib-based PROTACs (the figure was drawn by the authors using Chemdraw software).

Zhao and Burgess^63^ developed **RIB-POM** (Figure 39), a PROTAC consisting of pomalidomide, an alkyl-triazole linker, and ribociclib as the CDK4/6 ligand. **RIB-POM** was less efficient than **PAL-POM** in inducing CDK4/6 degradation but still achieved significant CDK4 depletion. Cytotoxicity assays in MDA-MB-231 cells indicated modest effects for **RIB-POM**, although solubility limitations prevented accurate quantification.

Su et al.^66^ found that ribociclib-based PROTACs had minimal effect on CDK6 degradation compared to palbociclib- and abemaciclib-based PROTACs. The authors attributed this to the specific binding mode of ribociclib - unlike palbociclib and abemaciclib, the terminal piperazine ring of ribociclib is situated further away from the solvent region, folding towards the C-terminal α-helix rather than extending into the solvent zone, with only a slight deviation towards the N-terminal β-sheet of CDK6. Due to the lower potency of ribociclib-based PROTACs, identifying the most potent variant was challenging.

Jiang et al.^64^ identified BSJ-01–152 and **BSJ-01–187** (Figure 39) as potent ribociclib-based CDK4 degraders with similar IC_50_ values. The primary structural difference between these compounds was the linker type - BSJ-01–152 utilised a PEG2 linker, while **BSJ-01–187** employed an alkyl linker. **BSJ-01–187** also induced the degradation of IKZF1/3. The study further demonstrated that modifying the thalidomide aryl amine nitrogen to an oxylamide moiety prevented IKZF1/3 from being recruited for degradation.

#### Abemaciclib-based PROTACs

##### Rational design and development of abemaciclib-based PROTACs

As with ribociclib, abemaciclib-based PROTACs were described in studies alongside palbociclib-based compounds. This section will, therefore, highlight only additional insights into the development and biological activity of abemaciclib-based PROTACs, omitting synthesis details, which largely mirror those for palbociclib-based PROTACs.

**Figure 40.**
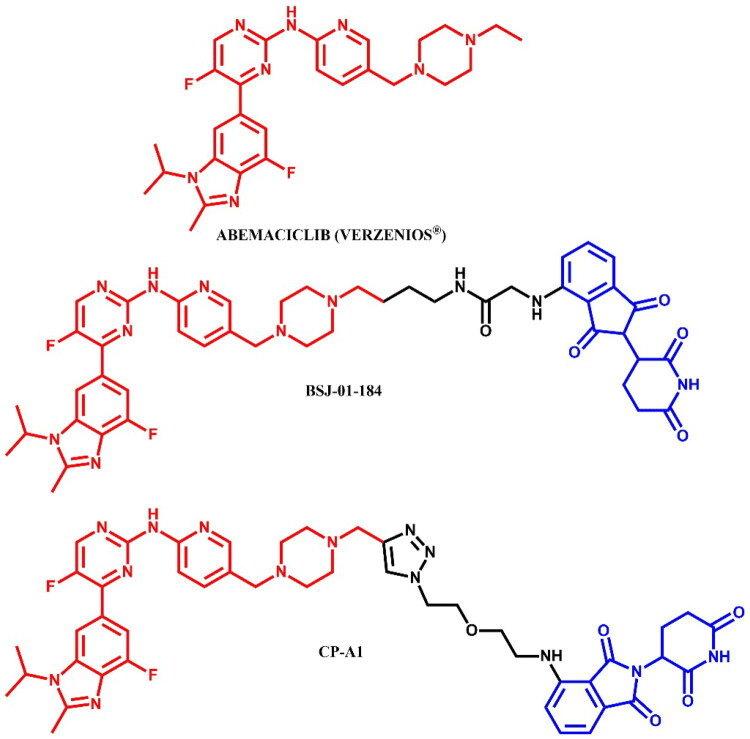
Structure of abemaciclib and abemaciclib-based PROTACs (the figure was drawn by the authors using Chemdraw software).

Jiang et al.^64^ tested only two abemaciclib-based PROTACs, both with a similar alkyl linker and pomalidomide moiety. Of all the PROTACs tested, the abemaciclib-based variants achieved the lowest IC_50_ values (for **BSJ-01–184**: IC_50_ = 8.61 nM (CDK4) and 12.5 nM (CDK6); for abemaciclib: IC_50_ = 4.77 nM (CDK4) and 8.5 nM (CDK6); for **YKL-06–102**: IC_50_ = 137.0 nM (CDK4) and 39.0 nM (CDK6); for **BSJ-02–162**: IC_50_ = 25.7 nM (CDK4) and 7.57 nM (CDK6)). In particular, **BSJ-01–184** (Figure 40) uniquely induced the degradation of CDK9, a known off-target.

In the study by Su et al.^66^, **CP-A1** (Figure 40) emerged as the most potent abemaciclib-based PROTAC, displaying degradation activity comparable to some palbociclib-based PROTACs, though still falling short of the most potent among them. This study limited the linker type to triazole-PEG, which may have restricted the full exploration of abemaciclib-based PROTAC potential. **CP-A1** exhibited selectivity for CDK6.

### ATC L01EJ and ATC L04AF – janus-associated kinase inhibitors (JAKi)

**Figure 41.**
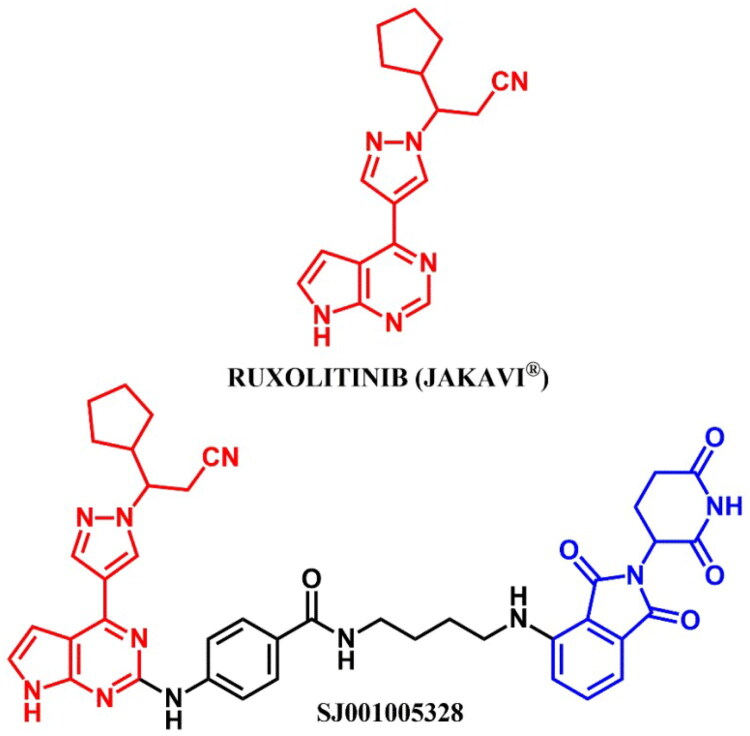
Structure of ruxolitinib and ruxolitinib-based PROTAC (the figure was drawn by the authors using Chemdraw software).

Janus kinase (JAK) inhibitors constitute a class of pharmaceuticals agents employed in the treatment of both cancer and immunological disorders^25^^,^^99^. In accordance with the ATC classification system, these drugs are grouped under the ATC L01EJ and ATC L04AF categories^20^. The L01EJ group includes JAK inhibitors that are predominantly used in the oncology category. These include momelotinib, pacritinib, fedratinib, and ruxolitinib^99^. It is worthy of note that ruxolitinib is also classified under ATC D11AH, a category covering non-steroidal treatments for dermatitis. In contrast, the L04AF group comprises JAK inhibitors used for immunological disorders, such as rheumatoid arthritis. This group encompasses a number of FDA-approved pharmaceuticals, including baricitinib, ritlecitinib, deucravacitinib, filgotinib, upadacitinib, and tofacitinib^99^. The mechanism of action of JAK inhibitors involves the inhibition of JAK kinases, which prevents the transmission of signals associated with cytokines and growth factors that are essential for cancer cell survival and inflammatory processes^99^. JAKi inhibit the tyrosine phosphorylation of JAK and subsequently inactivate the dimerisation of STAT-3. In another pathway, JAKi can inhibit the Ras/Raf pathway, thereby preventing the hyperphosphorylation of MAPK. This makes JAK inhibitors efficacious in the treatment of cancer and the management of autoimmune diseases. There are four distinct subtypes of JAK kinases – JAK1, JAK2, JAK3, and TYK2 - which are expressed by a multitude of cells. Among JAKi, ruxolitinib and baricitinib have been employed in the context of PROTAC technology (Figures 41 and 43).

#### Ruxolitinib-based PROTAC

##### Rational design and development of ruxolitinib-based PROTAC

The development of ruxolitinib-based PROTACs, as described^72^ in the patent WO 2021/022076, illustrated a significant step in targeting JAK2 kinases for degradation. In their research, the authors examined several aspects of these PROTACs, including protein degradation efficiency, cell permeability, SAR, immunoblot analysis, and cytotoxicity, as well as effects on the JAK-STAT signalling pathway in MHH-CALL-4 cells and preclinical *in vivo* studies. The most potent ruxolitinib-based PROTAC was **SJ001005328** (EC_50_ = 3.9 nM) (Figure 41). This PROTAC was a combination of ruxolitinib and an amino benzoic acid-based linker, featuring a four-carbon alkyl chain bound via an amide bond with pomalidomide. The design of **SJ001005328** was informed by the crystal structure of ruxolitinib bound to the JAK2 JH1 protein (PDB: 6VGL), which revealed crucial insights into the pyrimidine core’s conformation, solute-exposed regions, and potential linker attachment points. **SJ001005328** demonstrated robust degradation of JAK1, JAK2, GSPT1, and IKZF1, with moderate effects observed on JAK3 and TYK2.

##### Synthesis of ruxolitinib-based PROTAC

**Figure 42.**
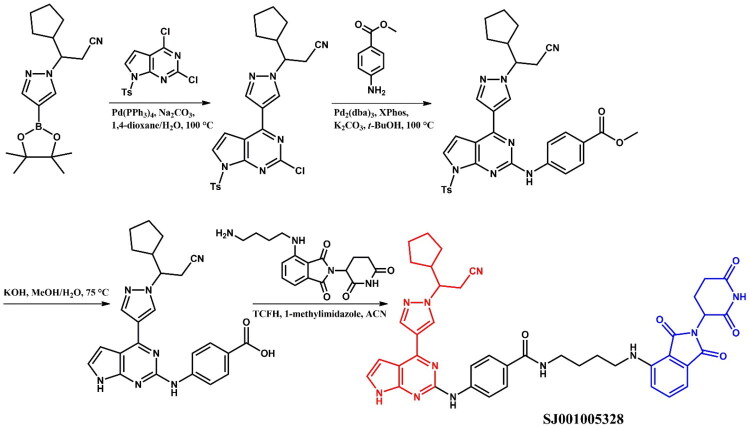
Routes of synthesis of ruxolitinib-based PROTAC (the figure was drawn by the authors using Chemdraw software).

The synthesis of **SJ001005328** was started with a Suzuki-Miyaura coupling, where tosylated 7H-pyrrolo[2,3-d]pyrimidine was introduced to an appropriate boronic acid pinacol ester pyrazole derivative (Figure 42). The resulting product then underwent a Buchwald-Hartwig reaction with methyl 4-aminobenzoate moiety. The tosyl group and the ester in the resulting material were hydrolysed under strongly basic conditions. In the final step, the product was coupled with a modified pomalidomide, which included an alkylamine group.

#### Baricitinib-based PROTACs

##### Rational design and development of baricitinib-based PROTACs

**Figure 43.**
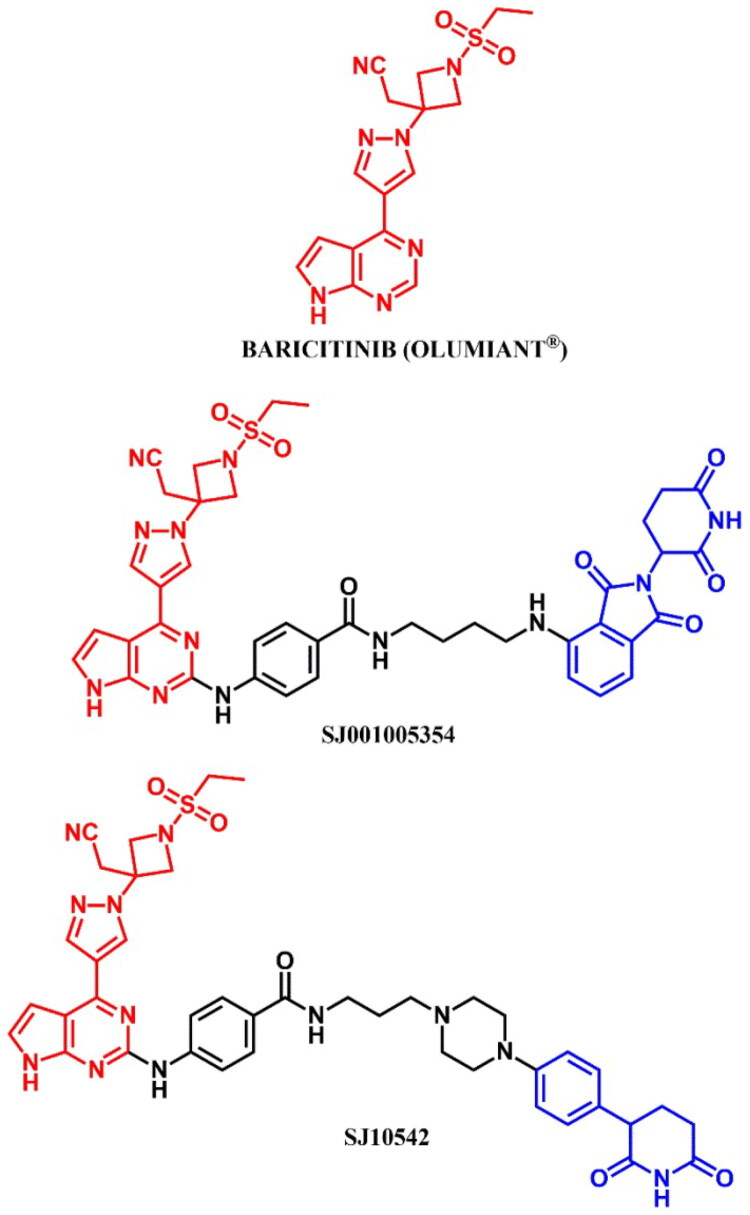
Structure of baricitinib and baricitinib-based PROTACs (the figure was drawn by the authors using Chemdraw software).

The same patent (WO 2021/022076)^72^ also described the development of baricitinib-based PROTACs. Similar studies to those conducted with ruxolitinib-based PROTACs were performed. The most potent baricitinib-based PROTAC identified was **SJ001005354** (EC_50_ = 0.54 nM) (Figure 43). **SJ001005354** demonstrated the same degradation efficiency as **SJ001005328**. Both PROTACs used the same linker and E3 ligase ligand.

Alcock et al.^73^ also constructed baricitinib-based PROTACs utilising a phenyl glutarimide (PG) ligand instead of the conventional IMiD-based ligands to minimise off-target degradation, particularly GSPT1. The most potent compound, **SJ10542** (Figure 43), employed baricitinib as the kinase-binding molecule with a phenyl-alkyl-piperazine linker and PG as the CRBN ligand. **SJ10542** was demonstrated to be efficacious in patient-derived ALL cells that exhibited JAK2 fusions (for JAK2: DC_50_ = 14 nM and D_max_ = 90%: for JAK3: DC_50_ = 11 nM and D_max_ = 92%) and CRLF2 rearrangements. This PROTAC demonstrated superior efficacy compared to its parental JAK2 inhibitors in the MHH-CALL-4 and KOPN49 cell lines, although complete efficacy was not achieved due to the redundancy of JAK2 in the JAK-STAT pathway. However, **SJ10542** showed potent activity in a number of patient-derived xenografts (PDX*) in vitro* models, with IC_50_ values less than 120 nM.

##### Synthesis of baricitinib-based PROTACs

**Figure 44.**
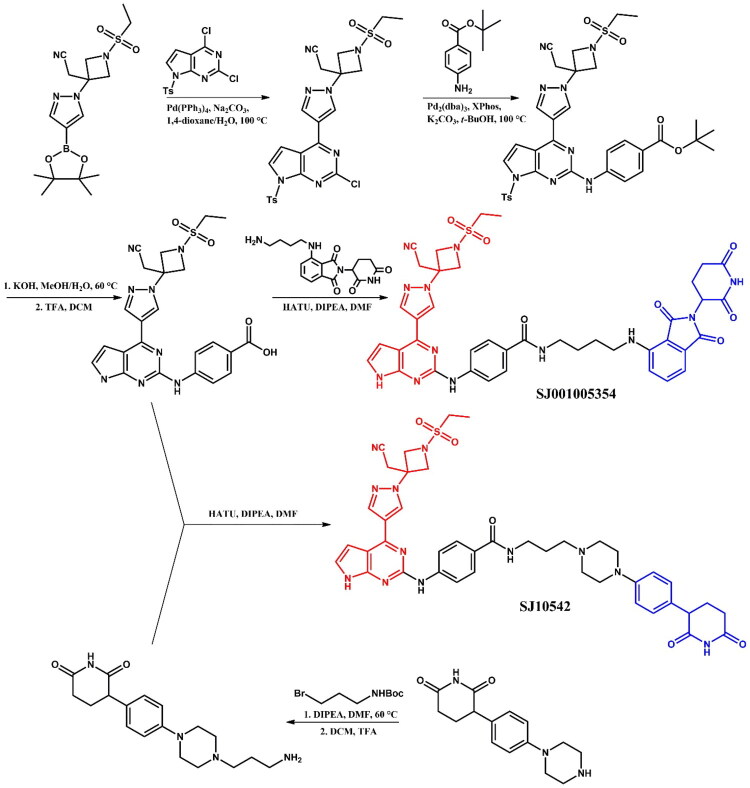
Routes of synthesis of baricitinib-based PROTACs (the figure was drawn by the authors using Chemdraw software).

The synthesis of **SJ001005354** followed a similar route to **SJ001005328**, with a key modification in the second step, where tert-butyl amino benzoate was used instead of amino benzoate (Figure 44). Additionally, the final coupling step differed, utilising HATU as the coupling reagent and DIPEA as the base. In the case of **SJ10542**, the synthesis followed an identical pathway until the final coupling reaction, which employed a different intermediate. This intermediate, a phenyl glutarimide derivative, was prepared through a separate route involving the substitution of the piperazine ring in a piperidine-2,6-dione derivative - featuring a para-substituted phenyl group - with a propane-1-amine moiety.

### ATC L01EL – Bruton’s tyrosine kinase inhibitors (BTKi)

**Figure 45.**
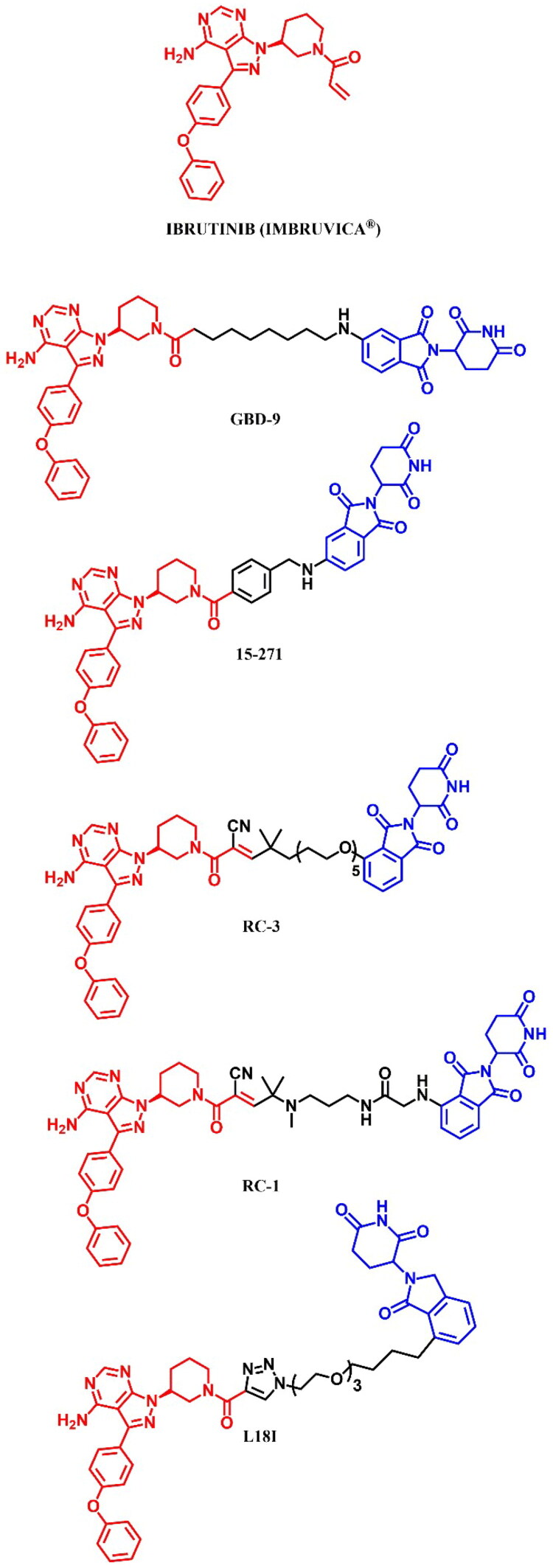
Structure of ibrutinib and ibrutinib-based PROTACs (the figure was drawn by the authors using Chemdraw software).

Bruton’s tyrosine kinase (BTK) is a non-receptor tyrosine kinase that plays a crucial role in the signal transduction of the B-cell antigen receptor in both normal and malignant B lymphocytes^100^. Activation of the BTK signalling pathway results in malignant B-cell proliferation and activation, contributing to the development of chronic lymphocytic leukaemia (CLL), MCL, diffuse large B-cell lymphoma (DLBCL), or follicular lymphoma (FL). Resistance to BTK inhibitors (BTKi) often arises from mutations in the BTK gene, the most clinically significant being C481S, which involves the substitution of cysteine with serine at position 481^101^. This mutation disrupts the covalent binding mechanism employed by first-generation inhibitors like ibrutinib, leading to treatment resistance. Four BTKi have been approved by the FDA for the treatment of these diseases: ibrutinib, zanubrutinib, acalabrutinib, and pirtobrutinib. However, only the first two have been utilised in PROTAC technology (Figures 45 and 48).

#### Ibrutinib-based PROTACs

##### Rational design and development of ibrutinib-based PROTACs

Yang et al.^74^ have developed **GBD-9** (Figure 45), a dual-mechanism degrader, which merges PROTAC and molecular glue properties for degrading BTK and GSPT1 (G1 to S phase transition 1 protein) concurrently. GSPT1 is a translational termination factor that plays a central role in mRNA translation and regulation of the G1/S checkpoint of the cell cycle, which contributes to tumour cell survival. **GBD-9** efficiently degraded BTK and GSPT1 proteins with high efficiency (BTK (90%) and GSPT1 (80%) at 50 nM concentration) and inhibited cancer cell growth by recruiting CRBN in a range of DLBCL and AML cell lines. Its anticancer activity was demonstrated to be greater than that of ibrutinib. Importantly, **GBD-9** retained robust activity against clinically relevant BTK mutants, such as C481S and C481T, which are known to confer resistance to covalent BTK inhibitors. The authors also emphasised the importance of linker optimisation in achieving productive ternary complex formation. The study showed that a shorter linker limited the molecular conformation, while 5-substituted pomalidomide had a more stretched conformation, which positively affected the efficiency of degradation.

Sun et al.^75^ designed, synthesised, and evaluated a novel BTK degrader **15–271** (Figure 45) with optimised bioavailability *in vitro* and *in vivo*. The study demonstrated that **15–271** exhibited superior solubility compared to ibrutinib and other reported BTK PROTACs^76^. Assessment of *in vitro* intrinsic clearance through microsomal stability assay indicated a high level of microsome stability. This information was translated into *in vivo* studies, wherein it was proven that **15–271** has a prolonged half-life.

Gabizon et al.^77^ focused on reversible and irreversible covalent mechanisms of action of PROTACs. Irreversible covalent binding may reduce potency by eliminating the catalytic nature of PROTACs, whereas reversible covalent binding may enhance potency and selectivity and extend the duration of action. In this study, a series of cyanoacrylamide-based reversible covalent PROTACs were developed by introducing an electrophile group into the structure and tested in comparison with their irreversible covalent and non-covalent PROTAC analogues. The result yielded the **RC-3** PROTAC (Figure 45), which was identified as a highly potent and selective reversible covalent PROTAC. However, despite its greater potency than non-covalent analogues to the wild-type BTK, **RC-3** was highly sensitive to the mutant BTK^C481S^, with a > 1000 fold reduction in potency. This was due to the inability to form a covalent bond with the -SH group in cysteine, which indicated that this approach would be ineffective for the potential treatment of mutant C481S DLBCL.

Similarly to the approach taken by Gabizon et al.^77^, Guo et al.^78^ developed also cyanoacrylamide-based reversible covalent BTK PROTAC **RC-1** (Figure 45). This molecule was observed to significantly enhance the intracellular accumulation of PROTAC, and was found to act as dual functional inhibitor and degrader. The authors suggested that the enhanced intracellular accumulation is attributable to the rapid and reversible reaction of **RC-1** with intracellular glutathione, as well as a reversible reaction between the cyanoacrylamide moiety and the thiol group in glutathione. This approach offered a potential solution to address intracellular accumulation issues and can be applied to other small molecules with poor permeability. However, in contrast to the findings of Gabizon et al.^77^, the study showed that **RC-1** was capable of degrading both the wild-type BTK and the BTK^C418S^ mutant with comparable potency, whereas **RC-3** was not. This suggested that the linker lengths are a crucial factor in this process. A further advantage of **RC-1** was its dual mechanism of action, which provides BTK inhibition in the event of incomplete BTK degradation within cells.

Sun et al.^76^ discovered PROTAC **L18I** (Figure 45) as potential treatment of ibrutinib-resistant non-Hodgkin lymphomas. The study showed that **L18I** efficiently degraded five single-point mutant BTKs at the 481 position related to cysteine substitution with tryptophan, serine (DC_50_ = 29.0 nM), alanine, threonine, or glycine. Moreover, **L18I** potently inhibited the proliferation of BTK^C481S^ mutant-expressing DLBCL and MCL cells, whereas ibrutinib exhibited very weak efficacy. But more importantly, **L18I** resulted in an antitumor effect in mouse inoculated with BTK^C481S^ HBL-1 cells, what was consistent with the results from the *in vitro* studies. In developing the process of **L18I**, aqueous solubility was improved by optimisation of both ligand and linker. In this case, ibrutinib and lenalidomide as ligands connected by a PEG linker with five-membered ring triazole were proved to be the most favourable for both *in vivo* and *in vitro* evaluations.

##### Synthesis of ibrutinib-based PROTACs

**Figure 46.**
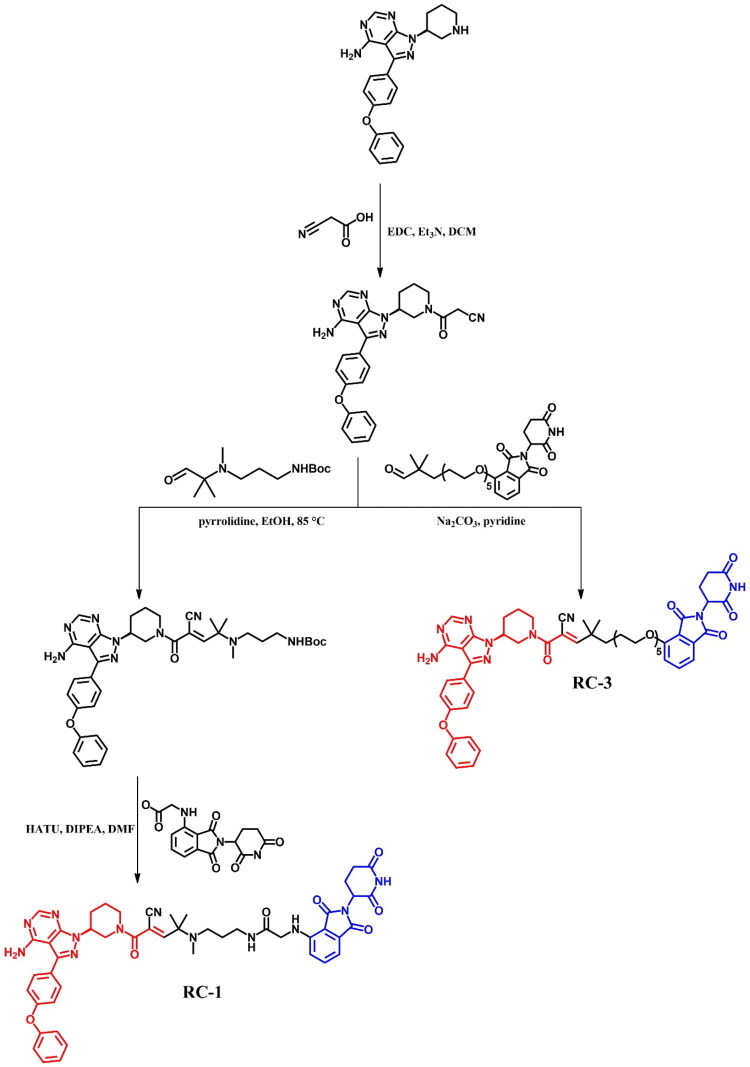
Routes of synthesis of ibrutinib-based PROTACs. Part I (the figure was drawn by the authors using Chemdraw software).

**Figure 47.**
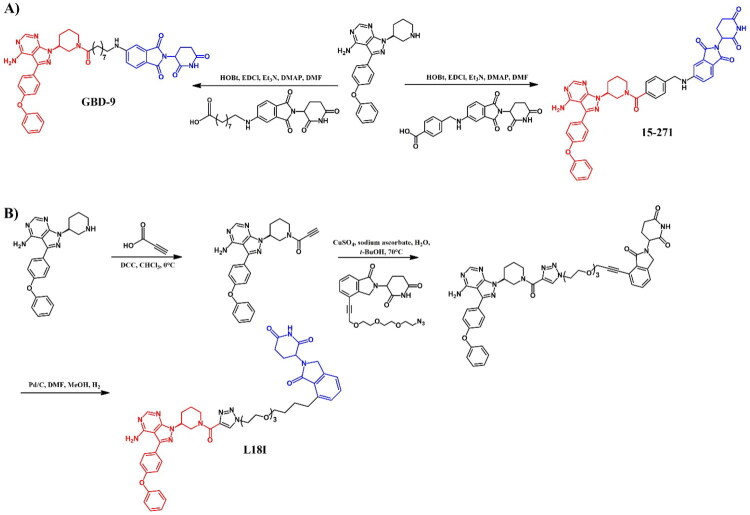
(a, b) Routes of synthesis of ibrutinib-based PROTACs. Part II (the figure was drawn by the authors using Chemdraw software).

The synthesis of ibrutinib-based PROTACs starting from the same initial substrate – the ibrutinib precursor containing a free amine moiety - followed a similar pattern, involving key functionalisation and coupling reactions (Figures 46 and 47). In the case of **RC-3** (Figure 46), ibrutinib precursor was functionalised by the introduction of an electrophilic group via coupling with cyanoacetic acid, followed by condensation with a thalidomide-PEG5 aldehyde. The synthesis of **RC-1** (Figure 46) mirrored that of **RC-3**, but with a condensation step involving respective N-protected aldehyde and coupling reaction with pomalidomide derivative containing a carboxylic group. In the synthesis of **GBD-9** (Figure 47a), 5-fluoropomalidomide underwent a (S_N_Ar) fluoride reaction with an amino acid containing 8- long alkyl chain, followed by coupling with ibrutinib precursor to form an amide bond. Similarly, for **15–271** (Figure 47a), the synthesis begun with an S_N_Ar reaction of 5-fluoropomalidomide with an aromatic amino acid, and the resulting pomalidomide derivative was coupled with ibrutinib precursor. Lastly, the synthesis of **L18I** (Figure 47b) involved the functionalisation of ibrutinib precursor through a coupling reaction with propiolic acid to introduce an alkyne group, followed by copper-catalysed azide-alkyne cycloaddition with lenalidomide-PEG3-azide. The triple bond in the resulting intermediate was then hydrogenated using palladium on carbon.

#### Zanubrutinib-based PROTAC

##### Rational design and development of zanubrutinib-based PROTAC

**Figure 48.**
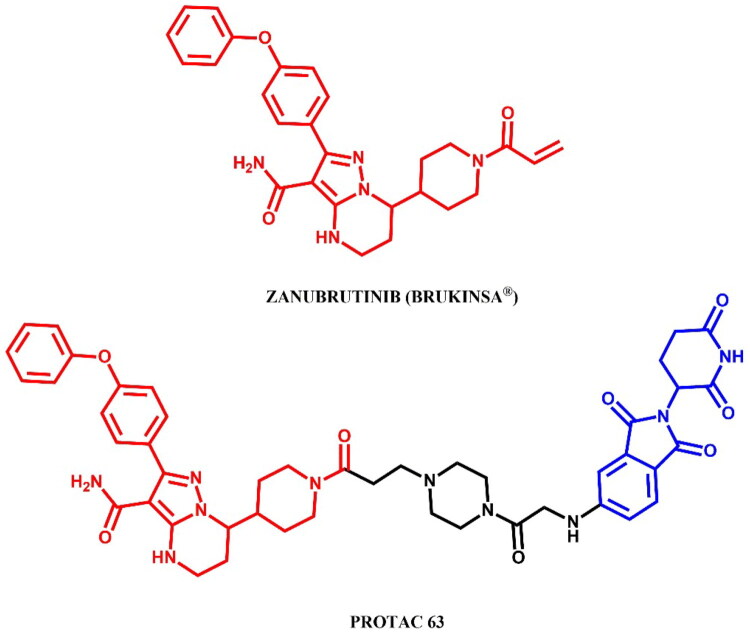
Structure of zanubrutinib and zanubrutinib-based PROTAC (the figure was drawn by the authors using Chemdraw software).

The patent WO 2021/018018^79^ described a library of 159 zanubrutinib-based PROTACs designed to recruit CRBN ligase for the degradation of wild-type BTK. The study employed a wide variety of linkers, including PEGs, alkyl chains, triazole moieties, aromatic and non-aromatic rings, heterocycles, spiro compounds, tertiary amines, and various combinations of them. Some linkers also featured additional amide bonds, which increased hydrogen bond acceptor (HBA) and hydrogen bond donor (HBD) parameters, influencing the physicochemical properties of this PROTACs. The most potent compound identified was **PROTAC 63** (Figure 48), with nanomolar activity (DC_50_ = 2.9 nM) in the BTK ELISA kit, which featured a piperazine moiety linked to the CRBN ligand at the position 5 C. Other promising PROTACs contained structural motifs such as cyclobutane, cyclohexane, tertiary amines, or triazole rings.

##### Synthesis of zanubrutinib-based PROTAC

**Figure 49.**
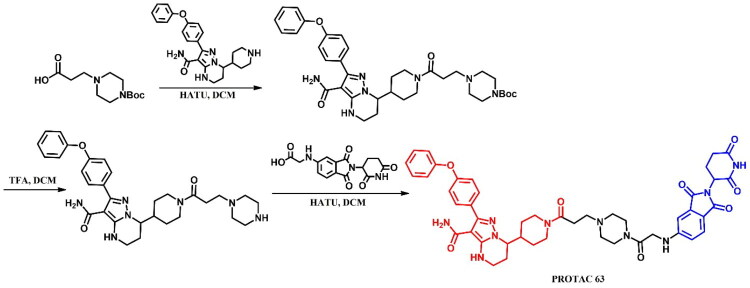
Route of synthesis of zanubrutinib-based PROTAC (the figure was drawn by the authors using Chemdraw software).

The synthesis of zanubrutinib-based **PROTAC 63** was realised by the coupling of Boc-protected piperazine derivative of propanoic acid and zanubrutinib precursor, which contained a free amine moiety in place of the amide group with a short alkene chain (Figure 49). Then, the resulting intermediate was deprotected and coupled with a thalidomide carboxylic acid derivative to complete the synthesis.

### ATC L01EX – other protein kinase inhibitors

**Figure 50.**
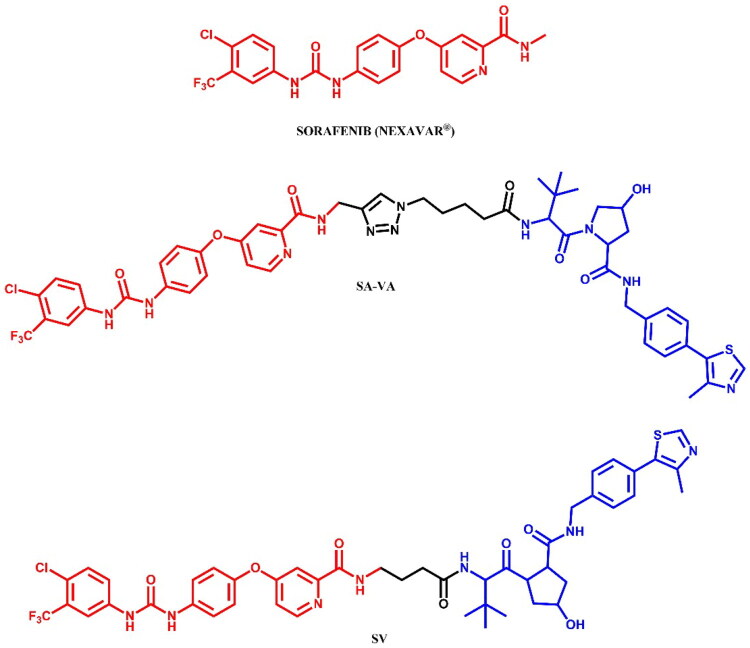
Structure of sorafenib and sorafenib-based PROTACs (the figure was drawn by the authors using Chemdraw software).

The ATC group L01EX is the largest and most diverse category of kinase inhibitors, with a primary focus on oncology. This group includes drugs that are multi-targeted without a clear main target and are not classified under more specific subgroups within the L01E category. A total of 22 FDA-approved drugs are included in this group: sorafenib, tepotinib, quizartinib, gilteritinib, entrectinib, capivasertib, midostaurin, nintedanib, lenvatinib, cabozantinib, regorafenib, vandetanib, pazopanib, sunitinib, umbralisib (withdrawn), pralsetinib, selpercatinib, ripretinib, avapritinib, capmatinib, pexidartinib, and larotrectinib. Nevertheless, only the first five of them have been utilised in PROTAC technology (Figures 50, 52, 54, 56, and 58).

Sorafenib is a multi-kinase inhibitor which decreases cancer cells proliferation by targeting various seronine-treonine kinases, including members of the RAF family and receptor tyrosine kinases such as c-KIT, FLT-3, VEGFR-2, VEGFR-3, and PDGFR-β^102^. Sorafenib is indicated for the treatment of unresectable hepatocellular carcinoma, advanced renal cell carcinoma, and differentiated thyroid carcinoma that is refractory to radioactive iodine treatment.

Tepotinib is a kinase inhibitor directed against MET (mesenchymal-epithelial transition factor), a receptor tyrosine kinase overexpressed or mutated in a variety of tumour types^103^. It plays a crucial role in the proliferation, survival, invasion, and mobilisation of tumour cells. Tepotinib is indicated for the treatment of NSCLC with alterations leading to MET factor gene exon 14 skipping.

Quizartinib is a fms-like tyrosine kinase 3 (FLT3) inhibitor that prevents autophosphorylation of the receptor, thereby inhibiting downstream FLT3 receptor signalling and blocking FLT3-ITD-dependent cell proliferation^104^. Quizartinib is indicated for the treatment of newly diagnosed AML in adults.

Gilteritinib is a selective inhibitor of both of the internal tandem duplication (ITD) and tyrosine kinase domain (TKD) mutations, of the FLT3 receptor, as well as AXL and ALK (anaplastic lymphoma kinase) tyrosine kinases^105^. The agent exerts its antineoplastic effect by inhibiting the phosphorylation of FLT3 and its downstream targets, such as STAT5, ERK, and AKT. Gilteritinib is indicated for the treatment of AML with a FLT3 mutation.

Entrectinib is an orally available, central nervous system (CNS) active, potent, and selective tyrosine kinase inhibitor which acts on several receptors – TRKA, TRKB, and TRKC – as well as proto-oncogene tyrosine-protein kinase ROS1 and ALK^106^. Entrectinib is indicated for the treatment of metastatic ROS1-positive NSCLC and neurotrophic tyrosine receptor kinase (NTRK) gene fusion-positive solid tumours.

#### Sorafenib-based PROTACs

##### Rational design and development of sorafenib-based PROTACs

Si et al.^80^ devised a methodology for the development of sorafenib-based PROTACs through an intracellular self-assembly strategy. In their study, the authors synthesised two types of precursors, each containing a biorthogonal group, specifically an azide and an alkyne. The core concept was that these precursors would undergo a reaction catalysed by endogenous copper ions, which are present in higher concentrations in tumour tissues. This reaction generated novel PROTAC molecules directly within cancer cells, potentially enhancing membrane permeability by reducing molecular mass and minimising side effects. The reaction between the azide and alkyne precursors was based on 1,3-dipolar cycloaddition, a click-chemistry ring formation reaction. To implement this strategy, the researchers modified the structure of sorafenib to create an alkyne derivative and modified the E3 ligase ligands into azide derivatives. The study demonstrated that this approach effectively led to the intracellular generation of PROTACs. The resulting PROTAC **SA-VA** (Figure 50) was observed to induce the degradation of VEGFR-2 and EphB4 in U87 cells in a concentration-dependent manner, thereby demonstrating the effectiveness of this innovative method for targeted protein degradation.

In addition, the same research group^81^ discovered three sorafenib-based PROTACs that selectively target and degrade PDGFR-β in a dose- and time-dependent manner. Among them, PROTAC **SV** (Figure 50) emerged as the most potent (in U87 cell line: IC_50_ = 12.34 μM; for sorafenib: IC_50_ = 12.67 μM), showing a significant reduction in PDGFR- β levels after 72 h of treatment, while sparing VEGFR-2 and other off-target proteins, though it had only a mild impact on cell cycle and apoptosis.

##### Synthesis of sorafenib-based PROTACs

**Figure 51.**
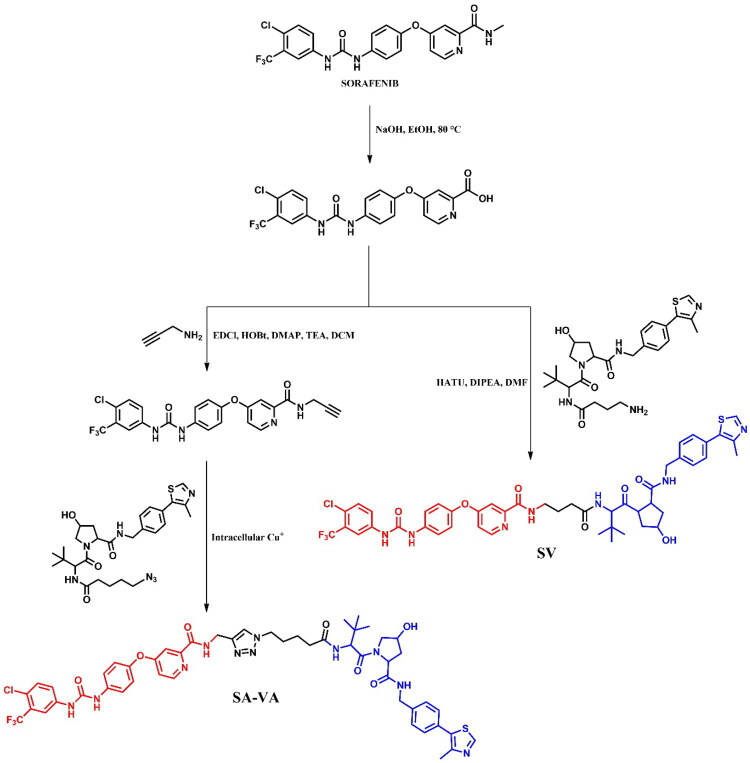
Routes of synthesis of sorafenib-based PROTACs (the figure was drawn by the authors using Chemdraw software).

The synthesis of both **SV** and **SA-VA** (Figure 51) began with the basic hydrolysis of the methylamide group in sorafenib, yielding a carboxylic acid, which subsequently underwent a coupling reaction with the corresponding amine. In the case of **SV**, this was a VHL ligand derivative linked with γ-aminobutyric acid, which resulted in the formation of the final product. In the synthesis of **SA-VA**, the carboxylic acid was coupled with a short alkyne amine. The resulting alkyne intermediate then underwent triazole ring formation via click chemistry reaction with an azide-functionalised VHL ligand in the presence of intracellular copper ions.

#### Tepotinib-based PROTAC

##### Rational design and development of tepotinib-based PROTAC

**Figure 52.**
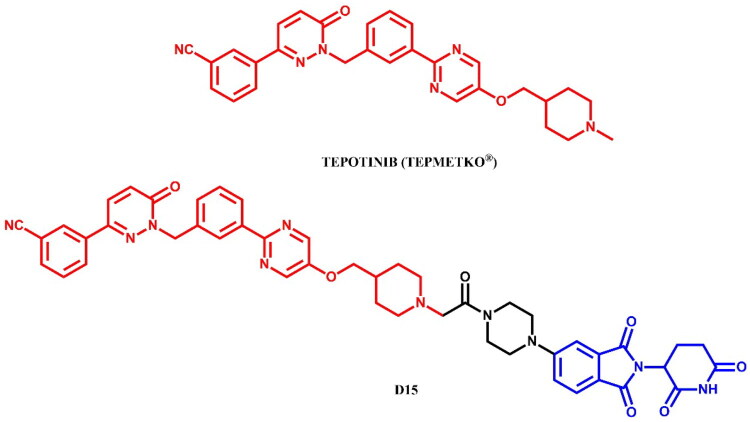
Structure of tepotinib and tepotinib-based PROTAC (the figure was drawn by the authors using Chemdraw software).

Li et al.^82^ designed, synthesised, and evaluated a series of highly potent and orally active tepotinib-based c-MET degraders with antitumour activity in cells with resistant to tepotinib c-MET^Y1230H^ and c-MET^D1228N^. The exceptionally potent molecule, **D15** (Figure 52), demonstrated inhibition of cell growth (in EBC-1 and Hs746T cell lines: IC_50_ = 2.11 nM and IC_50_ = 3.5 nM, respectively; for tepotinib: IC_50_ = 1.32 nM and IC_50_ = 2.63 nM, respectively) and achieved excellent picomolar DC_50_ values with >99% of maximum degradation (D_max_) in EBC-1 and Hs746T cells. Furthermore, **D15** also induced cell apoptosis, caused G1 cell cycle arrest, and inhibited cell migration and invasion *in vitro. In vivo*, oral administration of **D15** resulted in the near-complete suppression of tumour growth in the Hs746T xenograft model (tumour growth inhibition; TGI% = 99.2%). The rational design of **D15** was guided by the co-crystal structure of tepotinib with c-MET (PDB: 4RIV), in which tepotinib binds c-MET in a U-shaped conformation, with both the benzonitrile and methylpiperidine moieties exposed to the solvent. As the methylpiperidine group demonstrated no interaction with c-MET, it was identified as the optimal site for linker attachment. A variety of linkers, including PEGs, alkyl, and alkyl-piperazine, were evaluated for their degradation potency, substrate selectivity, and kinetics. **D15** exhibited a 10-fold greater antiproliferative effect than tepotinib in c-MET^Y1230H^ and c-MET^D1228N^ mutated cells (in EBC-1 cell line: IC_50_ = 80.98 nM and IC_50_ = 108.0 nM, respectively; for tepotinib: IC_50_ = 1.18 μM and IC_50_ = 0.95 μM, respectively), while demonstrating no cytotoxicity in normal cell lines (LO2, 293 T, HMEC, and BEAS-2B).

##### Synthesis of tepotinib-based PROTAC

**Figure 53.**
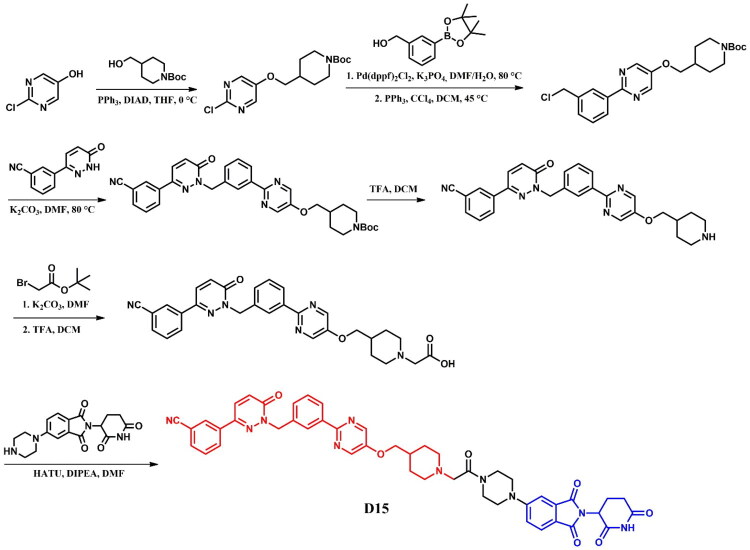
Routes of synthesis of tepotinib-based PROTAC (the figure was drawn by the authors using Chemdraw software).

The synthesis route of **D15** was started with the preparation of a respective tepotinib derivative, which was then coupled with a functionalised CRBN ligand (Figure 53). The synthesis was initiated with a Mitsunobu reaction between 2-chloropyrimidin-5-ol and an aliphatic alcohol, a group connected to piperidine ring. The forming ether was then subjected to a Suzuki-Miyaura reaction with an appropriate boronic acid pinacol ester alcohol derivative under standard conditions. Subsequently, the hydroxyl group in the obtained material underwent substitution by chloride in an Appel reaction. This substitution prepared the compound for further reactions, specifically the alkylation of the nitrogen atom in the heterocyclic ring of 2,3-dihydropyridazin-3-one. The resultant material was then deprotected and substituted with a tert-butyl 2-bromoacetatein order to extend the linker chain, followed by another deprotection step. In the final stage, the carboxylic acid reacted with the functionalised CRBN ligand, resulting in the formation of **D15**.

#### Quizartinib-based PROTACs

##### Rational design and development of quizartinib-based PROTACs

**Figure 54.**
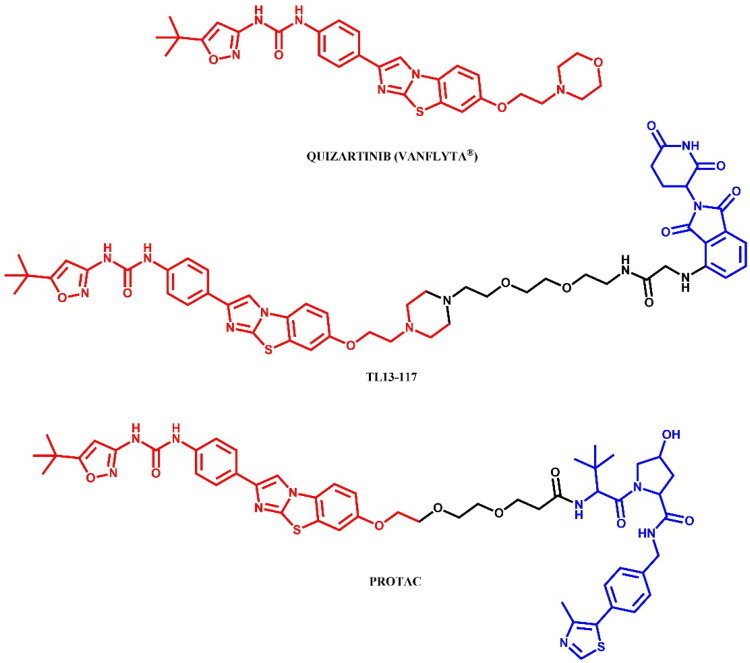
Structure of quizartinib and quizartinib-based PROTACs (the figure was drawn by the authors using Chemdraw software).

Huang et al.^83^ employed a chemoproteomic approach to explore the degradable kinome using a multi-kinase degrader and developed a selective FLT3 degrader, **TL13-117** (Figure 54), based on the structure of quizartinib. In the design, the morpholine ring of quizartinib was replaced with a piperazine ring, thereby enabling its conjugation to pomalidomide via a PEG linker. The study demonstrated that **TL13-117** selectively degraded FLT3 without affecting other kinases such as AURKA, BTK, or PTK2B, indicating a high degree of specificity, *In vitro* studies revealed that the CRBN-dependent pharmacology of **TL13-117** exhibited minimal improvement in antiproliferative effects when wild-type FLT3 cells were compared to CRBN^-/-^ cell lines, which lack cereblon. Notably, in both MOLM-14 and MV4-11 cell lines, quizartinib outperformed **TL13-117**, showing 5-fold lower IC_50_ values, indicative of enhanced antiproliferative potency. This finding suggested that despite FLT3 degradation, **TL13-117** did not significantly enhance the antiproliferative effect in comparison to quizartinib alone. The authors concluded that further optimisation is required to develop a PROTAC capable of more profound FLT3 degradation, with the potential to achieve a more effective therapeutic response by reducing FLT3 levels significantly below their endogenous baseline.

Burslem et al.^84^ successfully transformed quizartinib into a PROTAC capable of selectively inducing the degradation of the FLT-3 ITD mutant at low nanomolar concentrations. Remarkably, **PROTAC** (Figure 54) demonstrated enhanced inhibitory effect on cell growth compared to quizartinib alone (in MOLM-14 cell line: IC_50_ = 0.6 nM; for quizartinib: IC_50_ = 1.87 nM), while simultaneously affecting a reduced number of off-target kinases. Despite a slight reduction in the PROTAC’s kinase inhibitory activity, the enhanced antiproliferative effect was achieved through increased apoptosis induction, which suggested that FLT3-ITD may have non-kinase functions that contribute to its pathogenicity. Additionally, the **PROTAC** was observed to induce the degradation of FLT3-ITD *in vivo*.

##### Synthesis of quizartinib-based PROTACs

**Figure 55.**
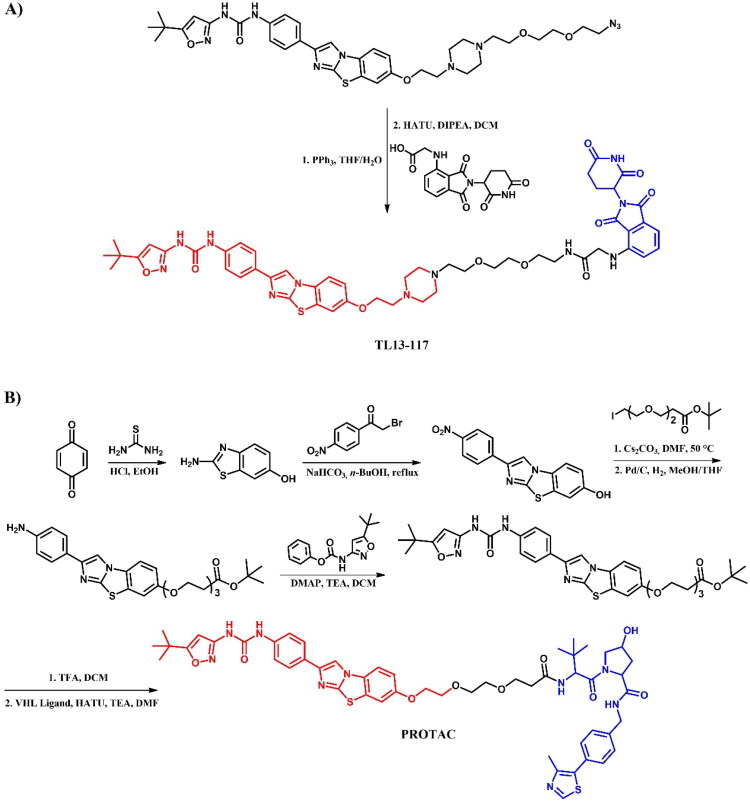
(a, b) Routes of synthesis of quizartinib-based PROTACs (the figure was drawn by the authors using Chemdraw software).

The synthesis of **TL13-117** started from the azide derivative of quizartinib (Figure 55a). The first step involved a Staudinger reduction. The obtained amine then underwent a coupling reaction with a carboxylic acid derivative of a CRBN ligand. In contrast, the synthesis of **PROTAC** (in this example, **PROTAC** is the name of PROTAC) was started with the reaction of 1,4-benzoquinonewith thiourea under acidic conditions to yield a benzothiazole derivative (Figure 55b). The resulting amino product then underwent cyclisation with an *p*-nitrophenacyl bromide, leading to the formation of a tricyclic derivative featuring one sulphur atom and two nitrogen atoms. The presence of a phenol group on one side of the obtained material enabled a substitution reaction with a protected iodo-PEG2-carboxylic acid. Subsequently, the nitro group on the opposing side was reduced via palladium on carbon to form an aromatic amine. This amine reacted with a carbamate derivative to yield a urea derivative. Following this, the obtained material was deprotected, and the resulting carboxylic acid was coupled with a VHL ligand.

#### Entrectinib-based PROTAC

##### Rational design and development of entrectinib-based PROTAC

**Figure 56.**
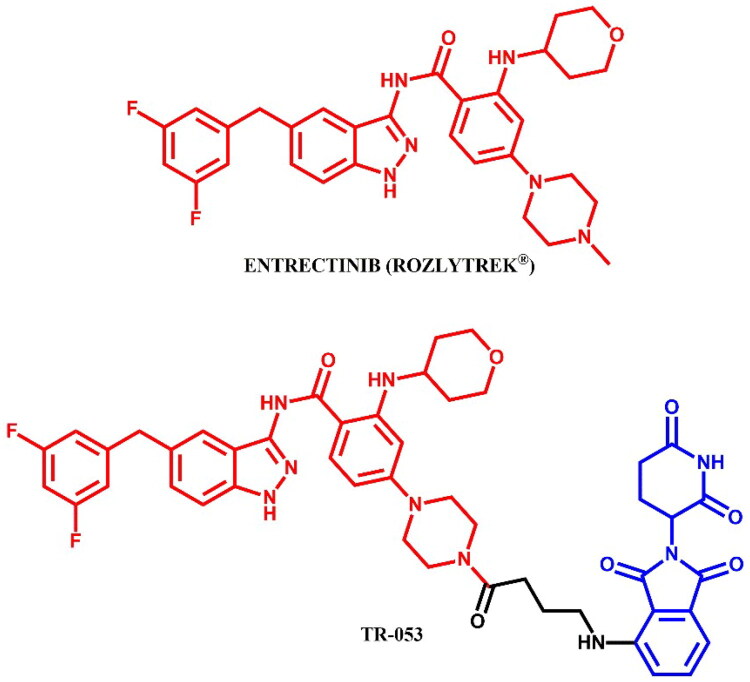
Structure of entrectinib and entrectinib-based PROTAC (the figure was drawn by the authors using Chemdraw software).

The patent WO 2020/038415^85^ described development a library of 516 PROTACs utilising either entrectinib or GNF-8625 scaffold as the POI ligand. The study evaluated the IC_50_ values for each compound and assessed their capacity to degrade TRK proteins. Additionally, *in vivo* xenograft mouse models were employed to determine the antitumour activity of selected PROTACs, and pharmacokinetic studies were conducted on four PROTACs (TR-123, TR-231, TR-275, and TR-198) to assess their behaviour following *i.p*., *i.v*., and *p.o.* administration. The study also explored the analgesic properties of one compound, TR-181, using monoiodoacetate (MIA)-induced pain models, which are frequently used to simulate osteoarthritis-related pain. The most potent entrectinib-based PROTAC, **TR-053** (Figure 56), exhibited superior activity in terms of both on IC_50_ and degradation capacity (IC_50_ = 1.5 nM and D_max_ > 80%). However, it was difficult to summarise the entire study due to the extensive range of PROTACs that were tested, each of which demonstrated varying outcomes across a multitude of experiments, including degradation potency, pharmacokinetics, and therapeutic efficacy.

##### Synthesis of entrectinib-based PROTAC

**Figure 57.**
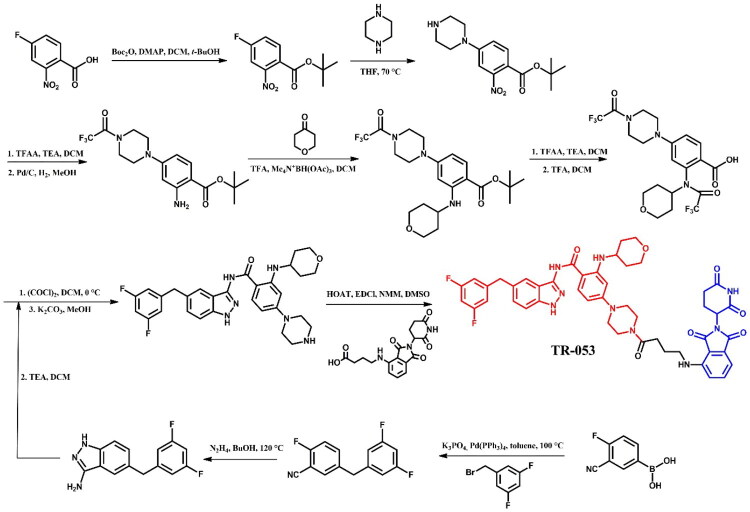
Routes of synthesis of entrectinib-based PROTAC (the figure was drawn by the authors using Chemdraw software).

The synthesis of **TR-053** (Figure 57) began from 4-fluoro-2-nitrobenzoic acid. This multi-step route involved initial carboxylic acid protection, followed by S_N_Ar with piperazine and subsequent selective protection of the free amine. The nitro group was then reduced, and the resulting amine underwent reductive amination to introduce a tetrahydropyran moiety. After a series of selective deprotections, the carboxylic acid was activated and coupled with a pre-synthesised benzimidazole derivative. The final step involved the removal of protecting groups and a coupling reaction with a pomalidomide carboxylic acid derivative to yield **TR-053**.

#### Gilteritinib-based PROTACs

##### Rational design and development of gilteritinib-based PROTACs

**Figure 58.**
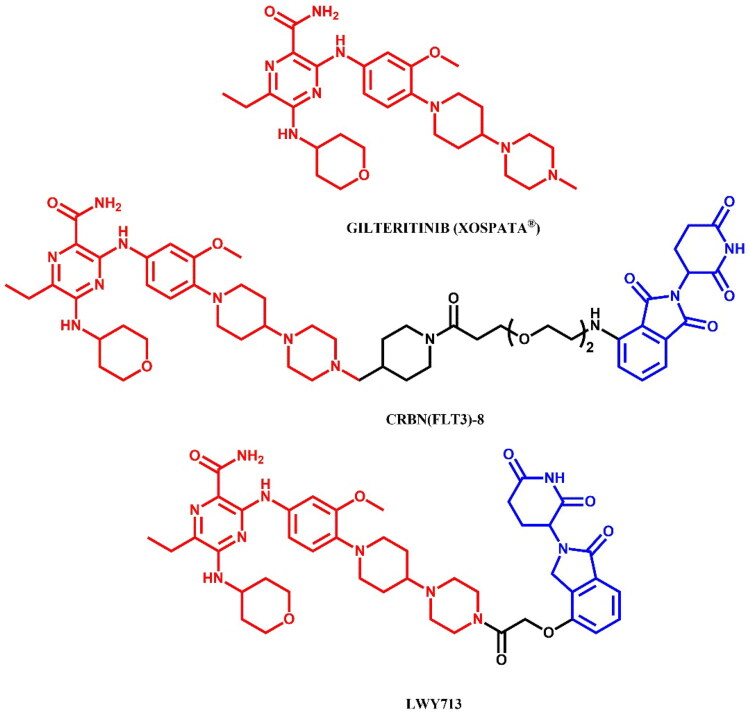
Structure of gilteritinib and gilteritinib-based PROTACs (the figure was drawn by the authors using Chemdraw software).

Ohoka et al.^86^ developed a novel FLT3 gilteritinib-based degrader, **CRBN(FLT3)-8** (Figure 58), which effectively degraded FLT3-ITD – a internal tandem duplication mutation in the FLT3 gene - and inhibited the proliferation of FLT3-ITD mutant AML cells more effectively than gilteritinib. Conversely, in cancer cells lines without the FLT3-ITD mutation (HeLa, MCF-7, and HT1080 cells), **CRBN(FLT3)-8** exhibited minimal proliferation inhibition, even at micromolar concentrations, and was significantly less effective than gilteritinib. This suggested that **CRBN(FLT3)-8** selectively targeted and inhibited the growth of cancer cells expressing the FLT3-ITD mutation. The authors also noted the occurrence of the hook effect. As gilteritinib is a dual inhibitor of FTL3 and AXL, the authors investigated the effect of **CRBN(FLT3)-8** on AXL levels. They found that **CRBN(FLT3)-8** weakly induced AXL degradation. They also confirmed that the phosphorylation level of FLT3 and its downstream effectors were effectively inhibited by **CRBN(FLT3)-8**, in a similar manner to the inhibition produced by FLT3 inhibitors.

In a related study, Liu at al.^87^ reported a series of potent and selective FLT3 gilteritinib-based degraders. The authors synthesised a series of derivatives with varying alkyl chain lengths and two CRBN ligands, thalidomide and lenalidomide, linked via *N*-alkyl or *O*-alkyl bonds. The most potent compound, **LWY713** (Figure 58), potently induced FLT3-ITD degradation (in MV4-11 cells: DC_50_ = 0.64 nM and D_max_ = 94.8%). The design of **LWY713** was guided by the co-crystal structure of the FLT3 protein with gilteritinib (PDB: 6JQR), which showed that the 1-methyl-4-(piperidin-4-yl)-piperazine group was solvent-exposed and could be used as a linker attachment site without affecting FLT3 binding affinity. Mechanistic studies revealed that **LWY713** selectively induced dose- and time-dependent FLT3 degradation in a cereblon- and proteasome-dependent manner, while also inhibiting FLT3 signalling, suppressing cell proliferation, and inducing cell G0/G1-phase cycle arrest and apoptosis in MV4-11 cells. Furthermore, **LWY713** effectively inhibited downstream FLT3 signalling pathways, including JAK/STAT5, RAS/MAPK, and PI3K/AKT, thereby exhibiting significant antitumour activity in MV4-11 xenograft models. **LWY713** did not degrade off-target kinases such as AXL, ALK, and LTK, thus further demonstrating its selectivity.

##### Synthesis of gilteritinib-based PROTACs

**Figure 59.**
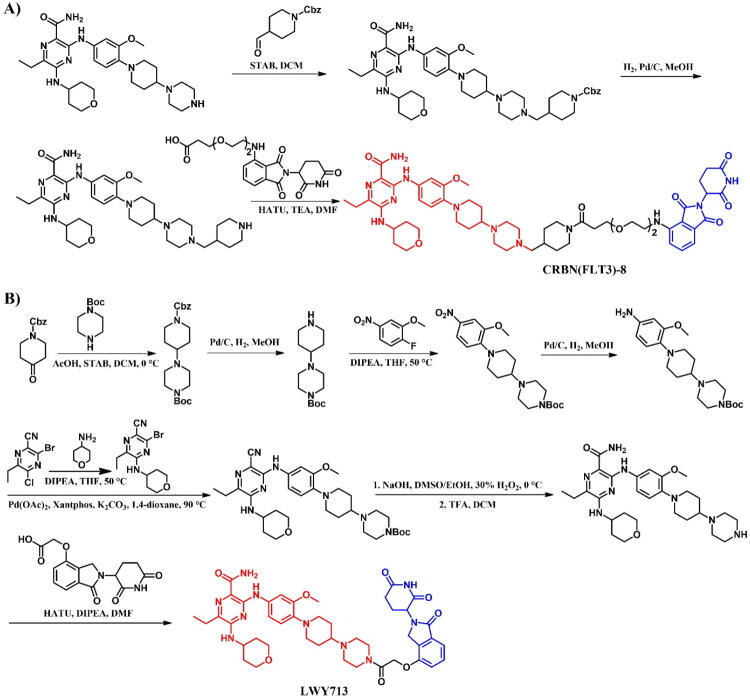
(a, b) Routes of synthesis of gilteritinib-based PROTACs (the figure was drawn by the authors using Chemdraw software).

**CRBN(FLT3)-8** synthesis was conducted starting from a gilteritinib precursor that differed from gilteritinib due to the removal of a methyl group from the piperazine moiety (Figure 59a). Its preparation involved a straightforward sequence of reductive amination and Cbz deprotection, followed by direct coupling with a pomalidomide-PEG2-carboxylic acid to yield the final PROTAC. In contrast, the synthesis of **LWY713** began with a reductive amination between Cbz-protected piperidin-4-one and Boc-protected piperazine (Figure 59b). Following reductive amination, the compound was selectively deprotected from its Cbz group via hydrogenation. Next, a S_N_Ar reaction was conducted with 1-fluoro-2-methoxy-4-nitrobenzene. The nitro group of the product was reduced to an amine, which then underwent Buchwald-Hartwig coupling with a previously synthesised intermediate derived from the reaction of oxan-4-amine with a pyrimidine halogen derivative via a S_N_Ar reaction. Subsequently, the nitrile group of the product was converted to an amide through a reaction and the product was deprotected and coupled with a lenalidomide carboxylic acid derivative to yield **LWY713**.

## Summary, conclusion, and future perspectives

FDA-approved kinase inhibitors represent a highly promising class of compounds for integration into PROTAC technology due to their well-established clinical efficacy, safety profiles, and known pharmacokinetics. These characteristics facilitate the rational design and optimisation of PROTACs, accelerating their preclinical and clinical development. This review highlights the significant progress made in employing FDA-approved kinase inhibitors as POI ligands in PROTACs, providing an overview of their application and associated challenges. A comparative summary of representative PROTACs is provided in Table 2.

**Table 2.** Comparative overview of PROTACs and their warheads targeting kinases, including key parameters (DC_50_, D_max_, IC_50_, EC_50_, K_d_) and tested cell lines.

Warhead	E3 ligase	PROTAC	DC_50_, D_max_, IC_50_, EC_50_, K_d_ (PROTAC)	IC_50_, EC_50_, K_d_ (Warhead)	Cell line
L01EA (BCR-ABL tyrosine kinase inhibitors)					
Dasatinib	CRBN	DAS-6-2-2-6-CRBN	D_max_ > 80% (at 2.5 μM)	–	K562
	IAP	SNIPER(ABL)-39	DC_50_ = 10.0 nM^i^ IC_50_ = 8.06 nM^i^ IC_50_ = 6.72 nM^ii^	–	KCL-22^i^ KU-812^ii^
	VHL	SIAIS178	DC_50_ = 8.5 nM IC_50_ = 24.0 nM	IC_50_ = 0.9 nM	K562
	RNF114	BT1	–	–	K562
	CRBN	Azo-PROTAC-4C	IC_50_ = 68.0 nM	–	K562
	CRBN	SIAIS056	DC_50_ = 0.18 nM IC_50_ = 0.49 nM	IC_50_ = 0.9 nM	K562
	CRBN	P22D1	IC_50_ = 0.57 μM	–	BaF3
	CRBN	DMP11	IC_50_ = 0.261 nM^i^ IC_50_ = 0.837 nM^ii^	IC_50_ = 0.027 nM^i^ IC_50_ = 6.36 nM^ii^	K562^i^ KA^ii^
	FEM1B	NJH-2-142	–	–	K562
	CRBN	SJ43489	DC_50_ = 0.8 nM IC_50_ = 217.3 nM	–	K562
	CRBN	DAS-5-oCRBN	D_max_ = 78% (at 100 nM)	–	CAL-148
Asciminib	VHL	GMB-805	DC_50_ = 30.0 nM IC_50_ = 169.0 nM	–	K562
	CRBN	P19As	DC_50_ = 200.0 nM	–	K562
Ponatinib	CRBN	P19P	DC_50_ = 20.0 nM^i^ IC_50_ = 13.1 nM^ii^	IC_50_ = 1.0 nM^ii^	K562^i^ BaF3^ii^
Bosutinib	CRBN	BOS-6-2-2-6-CRBN	D_max_ > 80% (at 2.5 μM)	–	K562
L01EB (EGFR tyrosine kinase inhibitors)					
Dacomitinib	VHL	PROTAC 13	DC_50_ = 3.57 nM^i^ IC_50_ = 6.0 nM^i^ IC_50_ > 20.0 μM^ii,iii,iv^	IC_50_ = 7.0 nM^i^ IC_50_ = 700.0 nM^ii^ IC_50_ = 1.79 μM^iii^ IC_50_ = 1.96 μM^iv^	HCC-827^i^ NCI-H1975^ii^ A549^iii^ A431^iv^
Rociletinib	CRBN	PROTAC 1Q	IC_50_ = 0.75 μM^i^ DC_50_ = 0.36 μM^i^ IC_50_ = 0.24 μM^ii^	IC_50_ = 0.27 μM^i^ IC_50_ = 0.13 μM^ii^	H1975^i^ PC-9^ii^
Osimertinib	CRBN	PROTAC 16 C	IC_50_ = 0.413 μM^i^ D_max_ = 78% (at 0.3 μM)^i^ IC_50_ = 1.34 μM^ii^ IC_50_ = 0.657 μM^iii^	–	PC9^i^ HCC827^ii^ H1975^iii^
Afatinib	VHL	PROTAC 4	D_max_ = 79.1%	–	H1975
Gefitinib	VHL	PROTAC 3	D_max_ = 98.9%^i^ D_max_ = 96.6% ^ii^	–	HCC827^i^ H3255^ii^
	VHL	MS39	K_d_ = 11.0 nM^i^ DC_50_ = 5.0 nM^i^ K_d_ = 12.0 nM^ii^ DC_50_ = 11.0 nM^ii^	K_d_ = 1.1 nM^i^ K_d_ = 0.8 nM^ii^	HCC-827^i^ H3255 ^ii^
	CRBN	MS154	K_d_ = 1.8 nM^i^ DC_50_ = 3.3 nM^i^ K_d_ = 3.8 nM^ii^ DC_50_ = 25 nM^ii^	K_d_ = 1.1 nM^i^ K_d_ = 0.8 nM^ii^	HCC-827^i^ H3255^ii^
	VHL	MS9449	K_d_ = 29.0 nM^i^ K_d_ = 6.4 nM^ii^	K_d_ = 1.1 nM^i^ K_d_ = 0.8 nM^ii^	HCC-827^i^ H3255^ii^
	CRBN	MS9427	K_d_ = 13.0 nM^i^ K_d_ = 2.9 nM^ii^	K_d_ = 1.1 nM^i^ K_d_ = 0.8 nM^ii^	HCC-827^i^ H3255^ii^
L01EC (BRAF serine-threonine kinase inhibitors)					
Dabrafenib	CRBN	PROTAC 19	IC_50_ = 0.413 μM^i^ D_max_ = 50%^i^ DC_50_ = 500.0 nM^i^ IC_50_ = 105.0 nM^ii^	IC_50_ = 13.0 nM^i^ IC_50_ = 619.0 nM^ii^	HEK293^i^ A375^ii^
	VHL	DD-03-156	–	–	–
Vemurafenib	VHL	SJF-0628	EC_50_ = 37.0 nM^i^ EC_50_ = 45.0 nM^ii^ EC_50_ = 218.0 nM^iii^	EC_50_ = 215.0 nM^i^ EC_50_ > 1 μM^ii^	SK-MEL-28^i^ SK-MEL-246^ii^ H1666^iii^
	VHL	CST905	IC_50_ = 61.0 nM DC_50_ = 1.0 μM	–	SK-MEL-28
	CRBN	PROTAC 12	IC_50_ = 500.0 nM^i^ IC_50_ = 124.0 nM^ii^	IC_50_ = 116.0 nM^i^ IC_50_ = 164.0 nM^ii^	A375^i^ HT-29^ii^
Encorafenib	CRBN	PROTAC 10	IC_50_ = 194.0 nM^i^ IC_50_ = 31.0 nM^ii^	–	A375^i^ Colo205^ii^
L01ED (ALK inhibitors)					
Brigatinib	VHL	SIAIS117	IC_50_ = 1.7 nM^i^ DC_50_ = 7.0 nM^i^ IC_50_ = 46.0 nM^ii^ IC_50_ = 165.7 nM^iii^	IC_50_ = 2.7 nM^i^ IC_50_ = 58.2 nM^ii^ IC_50_ = 535.7 nM^iii^	SR^i^ H2228^ii^ 293T^iii^
	CRBN	SIAIS164018	IC_50_ = 2.0 nM	IC_50_ = 3.3 nM	SR
	CRBN	SIAIS352008	DC_50_ = 0.288 nM^i^ DC_50_ = 0.784 nM^ii^	–	OVCAR-5^i^ CAOV4^ii^
	CRBN	SIAIS262039	DC_50_ = 0.411 nM^i^ DC_50_ = 0.436 nM^ii^	–	OVCAR-5^i^ CAOV4 ^ii^
Alectinib	CRBN	SIAIS091	IC_50_ = 0.5 nM^i^ IC_50_ = 3.3 nM^ii^	IC_50_ = 3.4 nM^i^	SR^i^ SR (ALK^G1202R^)^ii^
Crizotinib	CRBN	SIAIS001	IC_50_ = 4.7 nM	–	SR (ALK^G1202R^)
Ceritinib	CRBN	PROTAC 17	D_max_ = 83.6% (at 1.0 μM) ^i^ IC_50_ = 62.0 nM^i^ D_max_ = 85.0% (at 1.0 μM) ^ii^ IC_50_ = 42.0 nM^ii^	–	H3122^i^ Karpas 299^ii^
L01EF (CDK inhibitors)					
Palbociclib	CRBN	PROTAC 10/BSJ-02-162	DC_50_ = 9.1 nM^i^ DC_50_ = 8.0 nM^ii^	–	Jurkat (CDK4)^i^ Jurkat (CDK6)^ii^
	CRBN	CP-10	D_max_ = 89.0% (at 100 nM)^i^ DC_50_ = 2.1 nM^ii^	–	U251 (CDK6)^i^ U251 (CDK4)^ii^
	VHL	CST651	D_max_ = 95.0% (at 100 nM)	–	MM.1S
	CRBN	BSJ-03-123	–	–	MV4-11
	CRBN	YX-2-107	IC_50_ = 0.69 nM^i^ IC_50_ = 4.4 nM^ii^	IC_50_ = 11.0 nM^i^ IC_50_ = 9.5 nM^ii^	BV173 (CDK4) ^i^ BV173 (CDK6) ^ii^
	CRBN	PAL-POM	DC_50_ = 12.9 nM^i^ DC_50_ = 34.1 nM^ii^	–	MDA-MB-231 (CDK4) ^i^ MDA-MB-231 (CDK6) ^ii^
	VHL	MS28	DC_50_ = 0.95 μM D_max_ = 90.0%	–	Calu-1
	CRBN	YKL-06-102	IC_50_ = 137.0 nM^i^ IC_50_ = 39.0 nM^ii^	–	Jurkat (CDK4) ^i^ Jurkat (CDK6) ^ii^
Ribociclib	CRBN	RIB-POM	DC_50_ = 97.0 nM^i^ DC_50_ > 300.0 nM^ii^	–	MDA-MB-231 (CDK4) ^i^ MDA-MB-231 (CDK6) ^ii^
	CRBN	BSJ-01-187	–	–	Jurkat
Abemaciclib	CRBN	BSJ-01-184	IC_50_ = 8.61 nM^i^ IC_50_ = 12.5 nM^ii^	IC_50_ = 4.77 nM^i^ IC_50_ = 8.5 nM^ii^	Jurkat (CDK4)^i^ Jurkat (CDK6)^ii^
	CRBN	CP-A1	DC_50_ > 500.0 nM^i^ DC_50_ = 8.6 nM^ii^	–	U251 (CDK4)^i^ U251 (CDK6)^ii^
L01EJ/L04AF (JAK inhibitors)					
Ruxolitinib	CRBN	SJ001005328	EC_50_ = 3.9 nM	–	MHH-CALL-4
Baricitinib	CRBN	SJ001005354	EC_50_ = 0.54 nM	–	MHH-CALL-4
	CRBN	SJ10542	DC_50_ = 14.0 nM^i^ D_max_ = 90.0% ^i^ DC_50_ = 11.0 nM^ii^ D_max_ = 92.0%^ii^	–	MHH-CALL-4 (JAK2)^i^ MHH-CALL-4 (JAK3)^ii^
L01EL (BTK tyrosine kinase inhibitors)					
Ibrutinib	CRBN	GBD-9	D_max_ = 90.0% (at 50 nM)	–	Ramos
	CRBN	15-271	–	–	Ramos
	CRBN	RC-3	IC_50_ = 344.0 nM	–	MOLM-14
	CRBN	RC-1	IC_50_ = 309.0 nM^i^ IC_50_ = 82.0 nM^ii^	–	MOLM-14^i^ Mino^ii^
	CRBN	L18I	DC_50_ = 29.0 nM	–	HBL-1
Zanubrutinib	CRBN	PROTAC 63	DC_50_ = 2.9 nM	–	BTK ELISA Kit*
L01EX (other protein kinase inhibitors)					
Sorafenib	VHL	SA-VA	–	–	U87
	VHL	SV	IC_50_ = 12.34 μM	IC_50_ = 12.67 μM	U87
Tepotinib	CRBN	D15	IC_50_ = 2.11 nM^i^ IC_50_ = 3.5 nM^ii^ D_max_ > 99.0%^i, ii^ IC_50_ = 80.98 nM^iii^ IC_50_ = 108.0 nM^iv^	IC_50_ = 1.32 nM^i^ IC_50_ = 2.63 nM^ii^ IC_50_ = 1.18 μM^iii^ IC_50_ = 0.95 μM^iv^	EBC-1^i^ Hs746T^ii^ EBC-1 (c-MET^Y1230H^)^iii^ EBC-1 (c-MET^D1228N^)^iv^
Quizartinib	CRBN	TL13-117	–	–	MOLM-14
	VHL	PROTAC	IC_50_ = 0.6 nM	IC_50_ = 1.87 nM	MOLM-14
Entrectinib	CRBN	TR-053	IC_50_ = 1.5 nM D_max_ > 80.0%	–	CellTiter-Glo Kit*
Gilteritinib	CRBN	CRBN(FLT3)-8	–	–	MOLM-14
	CRBN	LWY713	DC_50_ = 0.64 nM D_max_ = 94.8%	–	MV4-11

^*^ Biological kits was used instead of cell lines

^i-iv^Indicate the corresponding cell lines for DC_50_, D_max_, IC_50_, EC_50_ and K_d_ for each kinase inhibitor. Each superscript refers to a specific cell line only in the context of a given kinase inhibitor, and the same superscript may correspond to different cell lines across different kinase inhibitors.

A key finding of this review is the pivotal role of rational design in addressing limitations inherent to kinase-based PROTACs. Studies consistently emphasise the necessity for precise optimisation of linkers, E3 ligase ligands, and the attachment points on the POI ligand, which must be carefully tailored for linker conjugation to maintain binding affinity, enhance degradation efficiency, and reduce off-target effects. Notably, variable PROTAC activity across different cell lines underscores the complexity of their biological effects, emphasising the need for extensive experimental validation and mechanistic studies to explain degradation patterns and cellular responses.

Crucially, converting already marketed kinase inhibitors into PROTACs holds significant potential to enhance their therapeutic efficacy compared to the original inhibitors. This enhancement stems from PROTAC mechanism of action, leading to sustained protein knockdown rather than transient inhibition. This distinct mode of action can translate into superior or more durable anti-proliferative effects in cancer potentially overcoming resistance mechanisms that limit the long-term effectiveness of conventional inhibitors. A significant advantage of this complete protein degradation is the ability of PROTACs to eliminate both the catalytic and non-catalytic (scaffolding, allosteric, protein-protein interaction) functions of kinases, functions often untouched by classical active-site inhibitors. While not universally achieved, PROTACs have demonstrated the ability to provide more sustained target engagement, leading to improved cellular responses and, in some cases, activity against resistant kinase mutations where the original inhibitor fails to eliminate the target protein.

Beyond rational design, synthetic efficiency is critical for accelerating PROTAC development. A well-planned synthetic strategy enables the rapid generation of diverse PROTAC molecules, allowing for high-throughput screening and faster identification of optimal degrader candidates. The widespread use of commercially available kinase inhibitors, E3 ligase ligands, and linker libraries reduces both the cost and time associated with PROTAC synthesis. FDA-approved kinase inhibitors possess functional groups (e.g. amines) that allow direct conjugation with linkers, while others require structural derivatisation to introduce a suitable attachment point. The predominant synthetic transformations employed in PROTAC synthesis include amide coupling reactions, as well as classical medicinal chemistry transformations such as aromatic nucleophilic substitutions, click-chemistry, and transition metal-catalysed Suzuki or Buchwald-Hartwig cross-coupling reactions. The use of robust and scalable synthetic methodologies facilitates the efficient production of PROTACs, ensuring reproducibility and feasibility for further preclinical and clinical development.

A critical consideration is whether modifying the same kinase inhibitor into various PROTACs leads to differences in efficacy, and the underlying reasons for these variations. Indeed, even subtle changes in linker chemistry (length, flexibility, hydrophobicity), E3 ligase ligand choice (e.g. cereblon vs. VHL), or the specific attachment point on the kinase inhibitor can profoundly impact PROTAC potency (IC_50_, DC_50_, D_max_), cooperativity of ternary complex formation, and cellular permeability. These differences arise from altered binding geometries within the ternary complex, variations in E3 ligase recruitment efficiency, and diverse pharmacokinetic properties. Consequently, extensive SAR studies for the PROTAC molecule as a whole, than just the warhead, are necessary.

Furthermore, the selectivity profile of kinase inhibitors can be significantly altered after their modification into PROTACs. While the original inhibitor might target a broad range of kinases, the PROTAC’s selectivity for degradation is dictated by the precise geometry and stability of the ternary complex formed with the E3 ligase. This can lead to either enhanced specificity, where only the intended kinase is efficiently degraded, or, conversely, to unexpected off-target degradation if other proteins form stable ternary complexes. Therefore, comprehensive selectivity profiling, extending beyond classical kinase inhibition panels to include proteome-wide degradation studies, is crucial for full characterisation.

Despite these advancements, significant challenges remain, including limited cellular permeability, metabolic instability, solubility constraints, kinase selectivity, and potential off-target effects. Emerging strategies such as incorporating photoswitchable elements^31^, designing dual-function molecules with molecular glue properties^74^, leveraging intracellular self-assembly^80^, and selective accumulation in tumour cells^78^ provide innovative solutions to overcome these barriers. Additionally, expanding the repertoire of E3 ligase ligands^30^^,^^35^^,^^36^^,^^62^^,^^73^ could enhance tissue specificity and broaden the scope of PROTAC applications.

Future research should focus on integrating unutilised FDA-approved kinase inhibitors into PROTAC technology to assess their degradation-based therapeutic potential in comparison to classical inhibition strategies. Moreover, newly developed kinase inhibitors, particularly those targeting previously undruggable kinases, should be systematically evaluated for PROTAC suitability. This evaluation should adhere to clear selection principles for kinase inhibitors that can be effectively modified into PROTACs. Ideal candidates should possess accessible functional groups for linker conjugation without compromising their binding affinity, exhibit favourable physiochemical properties (e.g. for cellular permeability), and ideally be suitable to diverse linker chemistries. Furthermore, their inherent selectivity for the target kinase is important, but it should be considered that binding affinity is not the sole determinant of successful degradation – crucial point is the ability to effective engagement of E3 ligase within ternary complex. Importantly, future publications concerning kinase inhibitor-based PROTACs should adopt a more systematic reporting standard. This includes consistently providing comprehensive degradation metrics such as IC_50_, DC_50_, D_max_, and where relevant, IC_50_ values of the parent inhibitor for direct comparison to the PROTAC. Furthermore, explicit comparisons to the activity of the bare warhead (kinase inhibitor alone) are crucial to ascertain whether its incorporation into a PROTAC leads to an advantage in terms of cellular outcome. Comparative analyses with other reported PROTACs targeting the same molecular objective or utilising the same warhead are also highly encouraged. Such rigorous data presentation across various cell models would significantly enhance the comparability of findings from different research groups, facilitating more robust evaluation and accelerating the translation of these promising compounds towards clinical development.

In summary, this review provides a comprehensive framework for understanding the integration of FDA-approved kinase inhibitors into PROTAC design. By bridging existing knowledge gaps and outlining critical experimental and conceptual advancements, it lays the foundation for future innovations, guiding the strategic utilisation of kinase inhibitors in targeted protein degradation.

## Data Availability

Data sharing is not applicable to this article as no new data were created or analysed in this study.
